# The value of biodiversity for the functioning of tropical forests: insurance effects during the first decade of the Sabah biodiversity experiment

**DOI:** 10.1098/rspb.2016.1451

**Published:** 2016-12-14

**Authors:** Sean L. Tuck, Michael J. O'Brien, Christopher D. Philipson, Philippe Saner, Matteo Tanadini, Dzaeman Dzulkifli, H. Charles J. Godfray, Elia Godoong, Reuben Nilus, Robert C. Ong, Bernhard Schmid, Waidi Sinun, Jake L. Snaddon, Martijn Snoep, Hamzah Tangki, John Tay, Philip Ulok, Yap Sau Wai, Maja Weilenmann, Glen Reynolds, Andy Hector

**Affiliations:** 1 Department of Plant Sciences, University of Oxford, South Parks Road, Oxford OX1 3RB, UK; 2 Consejo Superior de Investigaciones Científicas, Estación Experimental de Zonas Áridas, Carretera de Sacramento s/n, 04120 La Cañada, Almería, Spain; 3 Danum Valley Field Centre, The SE Asia Rainforest Research Partnership (SEARRP), PO Box 60282, 91112 Lahad Datu, Sabah, Malaysia; 4 Ecosystem Management, Institute of Terrestrial Ecosystems, ETH Zurich, Switzerland; 5 Department of Evolutionary Biology and Environmental Studies, University of Zurich, 8057 Zurich, Switzerland; 6 Tropical Rainforest Conservation and Research Centre, Lot 2900 and 2901, Jalan 7/71B Pinggiran Taman Tun, 60000 Kuala Lumpur, Malaysia; 7 Department of Zoology, University of Oxford, South Parks Road, Oxford OX1 3PS, UK; 8 Institute for Tropical Biology and Conservation, Universiti Malaysia Sabah, 88400 Sabah, Kota Kinabalu, Malaysia; 9 Sabah Forestry Department Forest Research Centre, Mile 14 Jalan Sepilok, 90000 Sandakan, Sabah, Malaysia; 10 Yayasan Sabah (Conservation and Environmental Management Division), 12th Floor, Menara Tun Mustapha, Yayasan Sabah, Likas Bay, PO Box 11622, 88813 Kota Kinabalu, Sabah; 11 Centre for Biological Sciences, University of Southampton, Southampton, UK; 12 Face the Future, Utrechtseweg 95, 3702 AA, Zeist, The Netherlands; 13 School of International Tropical Forestry, Universiti Malaysia Sabah, Kota Kinabalu, 88400 Sabah, Malaysia

**Keywords:** selective logging, tropical forest, forest restoration, biodiversity and ecosystem functioning, Sabah biodiversity experiment, Dipterocarpaceae

## Abstract

One of the main environmental threats in the tropics is selective logging, which has degraded large areas of forest. In southeast Asia, enrichment planting with seedlings of the dominant group of dipterocarp tree species aims to accelerate restoration of forest structure and functioning. The role of tree diversity in forest restoration is still unclear, but the ‘insurance hypothesis’ predicts that in temporally and spatially varying environments planting mixtures may stabilize functioning owing to differences in species traits and ecologies. To test for potential insurance effects, we analyse the patterns of seedling mortality and growth in monoculture and mixture plots over the first decade of the Sabah biodiversity experiment. Our results reveal the species differences required for potential insurance effects including a trade-off in which species with denser wood have lower growth rates but higher survival. This trade-off was consistent over time during the first decade, but growth and mortality varied spatially across our 500 ha experiment with species responding to changing conditions in different ways. Overall, average survival rates were extreme in monocultures than mixtures consistent with a potential insurance effect in which monocultures of poorly surviving species risk recruitment failure, whereas monocultures of species with high survival have rates of self-thinning that are potentially wasteful when seedling stocks are limited. Longer-term monitoring as species interactions strengthen will be needed to more comprehensively test to what degree mixtures of species spread risk and use limited seedling stocks more efficiently to increase diversity and restore ecosystem structure and functioning.

## 1. Introduction

After 20 years of debate, there is now broad consensus that biodiversity has a positive effect on the functioning and stability of ecosystems [1,2]. However, this consensus is founded on a first generation of research from grasslands and other easily manipulated systems, which are often short-term, small-scale and highly controlled experiments [3,4]. We need next-generation experiments to quantify how biodiversity affects ecosystem functioning in more natural and applied situations, including habitat restoration [3,5]. Experimental studies of the relationship between biodiversity and the functioning has only recently begun in a few locations in the tropics [6–10]. To help fill this knowledge gap for southeast (SE) Asian forests, we established the Sabah biodiversity experiment in Malaysian Borneo [11]. The project—a collaboration between ecologists, tropical foresters and a carbon offsetting scheme—tests the effects of tree diversity on the restoration of selectively logged forests which were enrichment planted with once-harvested species to return fully functioning ecosystems.

There are over 400 million hectares of logging estates and 350 million hectares of protection estates in the tropics—almost half the global tropical forest area when combined [12]. At least 20% of logging estates were selectively logged between 2000 and 2005 [12]. These recently logged forests now cover larger areas of land than primary forest in most regions [12,13]. In SE Asia, intact primary forest is largely restricted to highlands, after widespread logging and clearance for agriculture in lowlands [14,15]. In Sabah Malaysia, conversion to oil palm agriculture drove forest extent from 86% in 1953 to below 50% [16]. Premature harvesting of previously logged areas has been common [17]. This forest loss and degradation is threatening many SE Asian plants with population decline [14], including the dipterocarp trees that dominate these forests and which are a valuable timber source [18]. However, a growing body of evidence is showing that selectively logged forest harbours greater biodiversity than agricultural land and even fragmented primary forest within an agricultural landscape, so long as the logged forest is not further degraded by clearance, hunting and fires [12,19,20]. Some are calling to protect these vast areas from further land conversion, and maximize their conservation value by replanting with dipterocarps [21].

Enrichment planting is the practice of replanting seedlings into residual stands of selectively logged forest to restock target species, either permanently or for future harvests, while rehabilitating the degraded ecosystem. Tropical tree species are often naturally found at low density, so replanting logged species may help to supplement natural regeneration and overcome recruitment limitation. This might be particularly necessary for dipterocarps whose reproductive biology (irregular masting reproduction, low dispersal, no seed bank and vulnerable seedling banks) may jeopardize regeneration. Enrichment planting aims to overcome dispersal and recruitment limitation, speeding the return to tall, complex canopies. However, given the longevity of dipterocarps, evidence for the effectiveness of enrichment planting is incomplete despite its widespread implementation since the 1960s [22]. Success will depend on how many natural seedlings remain and whether enough planted seedlings survive to recreate the pre-logging canopy structure. Improvements in enrichment planting techniques since the 1960s have boosted the survival of planted trees, but our broader understanding of the role of enrichment planting for forest restoration is far from complete [22].

The effectiveness of enrichment planting can only be assessed once evidence gaps have been filled. One key issue is whether effectiveness is hampered by planting at low diversity; single species or mixtures of few species are typically planted over large areas. The survival and growth of commonly planted species and their environmental preferences are not well known, limiting the ability to match species with favourable planting sites. Whether species-site matching is at all feasible is unclear, because survival may vary over such fine spatial scales that its implementation is unrealistic and in most cases, records of pre-logging adult tree species distributions are absent. The role of tree diversity and how species combine in mixed-species plantings has received even less study.

The Sabah biodiversity experiment is part of a tree diversity experiment network and is currently, to our knowledge, the only representative in the palaeotropics [23]. The experiment manipulates the identity, composition and diversity of enrichment-planted dipterocarps to assess their impacts on the functioning and stability of selectively logged lowland rainforests during restoration [11]. Because planted seedlings were widely spaced (three per 10 m of planting line), and background vegetation after logging remains between planting lines, we did not expect to see biodiversity effects based on species interactions this early in the experiment. Even so, enrichment planting provides the potential for an insurance effect based on species differences in seedling mortality and growth. The usual practice in enrichment planting schemes is to stock large areas with low-diversity mixtures, often monocultures of seedlings available from nurseries. Monocultures run the risk of recruitment failure if the planted species turns out to be a poor survivor under the given circumstances; tree density may become so depleted that the planting does nothing to supplement natural regeneration [11,24]. Planting more diverse mixtures might provide an insurance against such recruitment failure. Diverse mixtures might also provide a more efficient use of seedlings by avoiding wasteful levels of self-thinning of species with high survival.

Here, we present the mortality and growth of the first cohort of enrichment-planted seedlings during the first decade of the project. We test a potential insurance effect of tree diversity in replanting schemes, in which mixtures avoid the potential twofold cost of monoculture planting: recruitment failure of the worst surviving species and wasteful self-thinning of the best.

## 2. Methods

### (a) Data collection

The Sabah biodiversity experiment [11] covers 500 ha in Malua Forest Reserve, a region of selectively logged forest bordering primary forest at Danum Valley conservation area located in the Malaysian state of Sabah, Borneo. Malua Forest Reserve, part of the Yayasan Sabah forest management area forest concession, was logged in the 1980s. Malua was logged between 1984 and 1986 and, except our experiment site, again in 2007. The anticipated harvest cycle is 50–60 years, the estimated time needed to achieve a species composition similar to unlogged forest [25]. The Yayasan Sabah (Sabah Foundation) concession also includes the 30 000 ha Innoprise-FACE Foundation Rainforest Rehabilitation Project (INFAPRO). The Sabah biodiversity experiment replicates INFAPRO's enrichment-planting techniques where possible to facilitate practical recommendations.

An advantage of the experiment's large spatial scale is that planted species are exposed to a range of environmental conditions, and we can explore the differences in species responses, a fundamental mechanism underlying the insurance hypothesis. Elevation at the site is less than 250 m, with 0–20° range in topography. Orthic acrisol soil on mudstone bedrock spans the entire site. The estimated pre-logging tree volume of Malua forest reserve was 193–221 m^3^ ha^−1^ [24]. The intensity and effect of logging was variable across the landscape; some areas have high bamboo cover, whereas others have mature trees remaining. Much variation in post-logging forest conditions occurs within 200 m (within plots, see below).

The experiment contains 124 four-hectare (200 × 200 m) plots, split between two blocks that are north and south of a logging road (see fig. 1 in [11] or electronic supplementary material, figure S1). There are 60 plots in the north block and 64 in the south block. Seedlings were planted 3 m apart along parallel planting lines in a stratified randomized design. Planting lines were kept clear of seedlings, shrubs, bamboo and lianas at a width of 2 m. Each plot contains 20 planting lines spaced 10 m apart. Post-logging forest conditions among plots were spatially independent (electronic supplementary material, Analysis), and plot treatments were randomly allocated within blocks. Ninety-six of these plots comprise a diversity gradient treatment. The remainder are comprised 12 unplanted controls, and another 16 of 16-species mixtures that were given enhanced climber cutting (electronic supplementary material, figure S1). Only the 96 diversity gradient plots are analysed here.

The diversity gradient manipulates species richness using a factorial design, including replicated species compositions within species richness levels (one, four and 16 species). Species compositions within the four-species mixtures provide a gradient of generic richness and are designed to produce a range of canopy structures once the planted seedlings mature (electronic supplementary material, table S2). Each species richness level has 32 plots. The one- and four-species richness levels contain 16 different compositions, each replicated twice (electronic supplementary material, table S3). Compositions were replicated evenly across blocks. In the enhanced climber-cutting treatment, climbers are cut throughout the whole plot, not just along the lines as in standard enrichment line planting—this is said to improve recovery time during restoration.

As with standard enrichment-planting practice, following early mortality, the initial planting cohort of seedlings (cohort 1 planted January 2002–September 2003) were supplemented with a replanting cohort (cohort 2 planted September 2008–August 2011). Across both cohorts, a total of 96 369 seedlings have been surveyed. Owing to the scale of the experiment, each full survey took 2 years to conduct (see the electronic supplementary material, Analysis, for histograms of seedling age); additionally, to complement this large-scale but time-consuming monitoring, a subset of plots have been more intensively sampled (six extra surveys to date [26]). Thus, in 10 years, there have been two surveys of all seedlings. The first survey (November 2003–May 2005) included only the first cohort of seedlings, whereas the second survey included both cohorts (November 2011–September 2013).

Here, we analyse survival and growth of the first cohort, using both full surveys (the second cohort can only be studied after repeated measurement at the next survey). We recorded survival and size for every seedling each time they were visited. We measured basal diameter (2 cm above ground) and, when they were tall enough, diameter at breast height (1.3 m).

### (b) Study species

The 267 species of dipterocarp known to occur in Malaysian Borneo belong to nine diverse genera—and roughly half of these species belong to one genus, *Shorea* [27]. The 16 species we planted are *Dipterocarpus conformis* Slooten, *Dryobalanops lanceolata* Burck, *Hopea ferruginea* Parij, *Hopea sangal* Korth., *Parashorea malaanonan* (Blanco) Merr., *Parashorea tomentella* (Blanco) Merr., *Shorea argentifolia* Sym., *Shorea beccariana* Bruck, *Shorea faguetiana* Heim., *Shorea gibbosa* Brandis., *Shorea johorensis* Foxw., *Shorea leprosula* Miq., *Shorea macrophylla* Ashton, *Shorea macroptera* King, *Shorea ovalis* Korth. and *Shorea parvifolia* Dyer (electronic supplementary material, table S1).

All species except *D. conformis* are members of the Shoreae tribe—though *Dipterocarpus* is sister to Shoreae and there is mixed support for the monophyly of Shoreae within this clade [28]. *Shorea, Parashorea* and *Hopea* form a polyphyletic group. Several sections within *Shorea*, covering multiple commercial timber types, are represented within our species [29]. Our species were selected because they: (i) represented those found in the surrounding forest [11], (ii) cover a range of traits and ecological strategies, and (iii) were sufficiently available as seedlings when first planted. The seedlings initially planted were sourced from INFAPRO; a dedicated project nursery was later set up to cultivate newly collected seedlings for the second cohort, other species that were too scarce for the main experiment have been studied in smaller associated experiments manipulating light and water [30–35], producing data on a total of 28 species.

SE Asian dipterocarps are mainly emergent, shade-tolerant trees concentrated in aseasonally wet evergreen lowland forest on well-drained soils. They are mostly found below 800 m altitude, and their abundance and diversity declines above 400 m. They produce seeds during mast fruiting events. If these seeds do not germinate quickly, then they die owing to heavy predation [36] or recalcitrance [33]. Surviving seeds produce a seedling bank. There is some evidence for a trade-off, particularly at the juvenile stage, between growth and survival [26,37–39]. Dipterocarps reach peak biomass, density and species richness on yellow or red lowland soils, where they comprise more than 50% above-ground tree biomass and more than 70% of emergent individuals [27]. It is the dipterocarps that give these forests their exceptionally high biomass for tropical forests [40]. In the 1980s, dipterocarps provided 25% of tropical hardwood supply worldwide, and 80% of this share came just from *Shorea* [29]. Juveniles are easily disturbed during logging, undermining their regeneration; they may not return for centuries in heavily degraded soils [27]. Palaeoecological work has shown that SE Asian tropical forests often take centuries to fully recover from disturbance—longer than any other tropical region [41].

### (c) Data analysis

Every seedling had its survival and size recorded in each survey (1 = alive, 0 = dead). For cohort 1, there are two surveys of all seedlings, with median age of 2.0 years (1.3–5.8) at survey 1 and 10.0 years (9.3–10.7) at survey 2. We chose to aggregate data on the survival and growth of each species within plots, i.e. the species-within-plot level (see the electronic supplementary material, Analysis). We could not analyse survival at the individual seedling level, because there were cases within the lowest grouping level where all members of a species either died or survived, pushing estimation to the parameter space boundary and causing model convergence failure. We chose to aggregate both the survival and growth data to make results of both models comparable, and to remove spatial correlation at the plot scale (within 200 m) that would leave spatial structure in the residuals. On average, these species-within-plot aggregations were based on 37 observations (16–1045) of individual tree survival (electronic supplementary material, Analysis). This left us with 1336 plot-level observations and a minimum of three replicates for any species within a species composition (across both surveys).

Survival and growth were modelled separately by fitting two linear mixed-effects models. Our response variable for the survival model was the proportion of planted seedlings remaining in a plot in a given survey. For the growth model, our response variable was the average log-transformed basal diameter of surviving seedlings in a given survey, and growth was assessed as daily change in diameter between a pair of survey dates (relative growth rate, RGR). We kept explanatory variables as consistent as possible to help compare survival and growth: species-within-plot was fitted as a random effect (one variance for a factor with 672 levels), and the fixed effects were a three-way interaction between species identity (16 levels), species composition (33 levels; 16 monocultures, 16 four-species mixtures, plus the 16-species mixture), plus a representation of survey time that differed between models as explained below (16 monocultures, 16 species within the 16-species mixture, plus four species within each of the 16 four-species mixtures gives 96 species-within-composition levels). For survival, survey time was a factor with two levels, giving the average proportional survival since planting for each survey (survey 1: 0–2 years since planting; survey 2: 0–10 years since planting). For growth, instead of treating surveys as a factor, survey time was continuous (number of days since planting). The slopes of change in log size between the two surveys gave our estimated growth RGR. Growth was therefore analysed using a subset of the survival data, using only seedlings alive at both surveys (1122 plot-level observations). Both models estimated 193 parameters: one additional variance component and 192 fixed effects. For each of the 96 species-within-composition levels, the survival model estimated two intercepts, whereas the growth estimated one intercept and one slope. These models were fitted using lme4 v1.1-7 [42] in R v3.2.1 [43]*.* Their model formulae were:
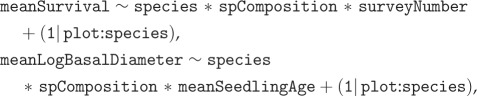
where each term is defined above.

To quantify each species’ overall performance, among species compositions, we took the average of their population-level predicted values from the models. These species-level estimates of growth and survival in each survey were used to assess how strongly species differ, whether their ranking in survival remains consistent over time, and whether they trade-off survival against growth. We correlated survival and growth with wood density and specific leaf area, which were estimated from previous experiments within our site (using the same seedling cohort) [32–34,44].

Spatial variation in species survival was quantified using predictions from the random effect—a plot-level deviation from the average survival for each species. By tracking the relative effect of every 16-species mixture plot upon each species, we could show whether species were responding differently to the same conditions. We tested whether species were responding differently to plot conditions by fitting two non-nested models: one allowing species-specific responses to plot conditions and another assuming species respond equally (species-specific responses, (1|plot:species) were compared with(1|plot)). We compared these models by seeing how much Akaike information criterion (AIC) improved when species-specific responses were allowed [45].

Finally, we summarized overall plot-level performance, averaging across species, as density of surviving seedlings; this was plotted against species richness, and then broken down to specific compositions, to assess whether a spatial insurance effect might confer an advantage to planting more diverse tree mixtures.

## 3. Results

**Figure 1.**
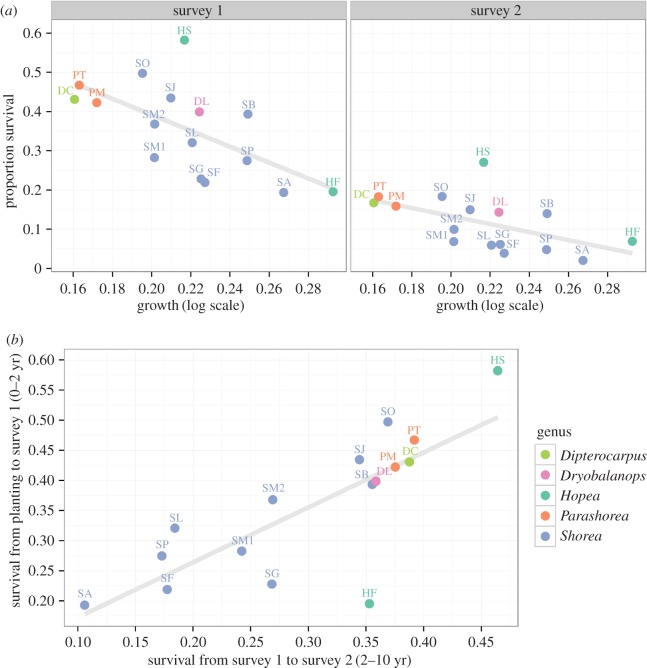
(*a*) Proportional survival (at approx. 2 and 10 years since planting) versus growth rate (change in log basal diameter between survey 1 and 2) for the 16 species, showing a negative trade-off. (*b*) The proportion of seedlings that survived 0–2 years versus 2–10 years since planting are positively correlated, showing consistent survival rates over the first decade. Species codes are shown in the electronic supplementary material, table S1. Grey regression lines show overall trends. (Online version in colour.)

Seedling survival and growth varied widely among species, after two and 10 years since they were planted (figure 1; for estimates see the electronic supplementary material, Analysis). The proportion of first-cohort seedlings that survived overall was low (0.36 after 2 years and 0.12 after 10 years). Species ranking in survival was consistent over the two surveys (figure 1; Pearson's *r* = 0.79). After 10 years, the seedlings had grown to an average apex height of 1.25 m (max. = 12 m) and average basal diameter of 1.6 cm (max. = 28 cm). There was a trade-off between survival and growth among species—though this fades over time as mortality mounts and proportional survival shrinks (0–2 years, *r* = −0.63; 2–10 years, *r* = −0.43).

We correlated survival and growth after 10 years with some traits that have been found to link to ecological strategies including in some of our previous work [26,37,46,47]. Wood densities for all species (excluding *H. ferruginea* whose high mortality prevented trait estimation) positively correlated with survival after 10 years (*r* = 0.78) and negatively correlated with growth (*r* = −0.50). Specific leaf area was weakly correlated with survival (*r* = 0.06) and growth (*r* = −0.14).

**Figure 2.**
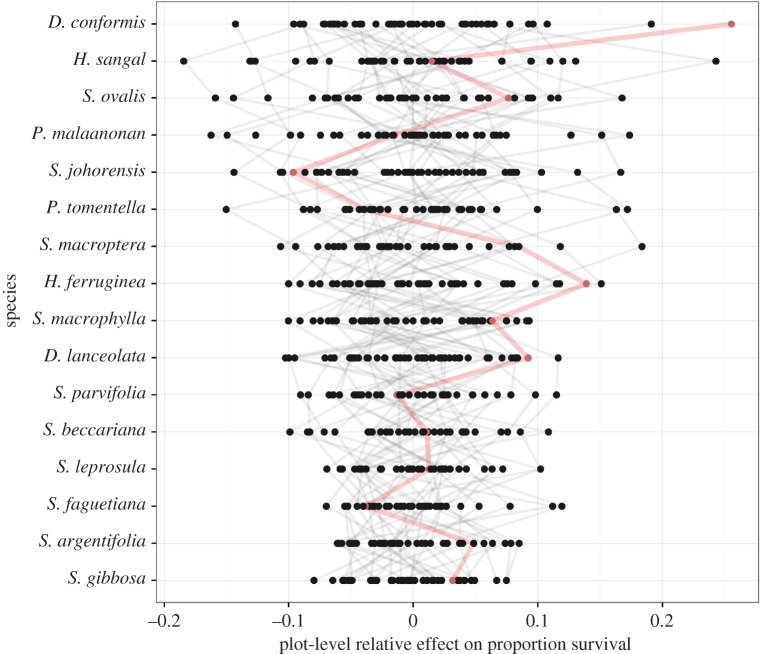
The species-by-environment interaction for seedling survival in the 16-species mixtures. Points represent the average survival of a species in a plot relative to the overall average of that species (from the plot:species random effect)—so positive values show plots with better-than-average survival. Grey lines join particular plots, illustrating the varying performance of different species in the same conditions. The thicker line coloured in red gives one example: while this plot shows above-average survival for some species (e.g. *D. conformis* shows its highest survival) other species experience below-average survival. (Online version in colour.)

A buffering effect of increased tree diversity may occur if species show varied responses to spatial variation and respond independently or asynchronously to one another. All species showed substantial spatial variation in survival and growth across the 500 ha experiment, though some more than others (figure 2). The species that showed the most variable survival across the experiment were not necessarily those that showed the most variable growth (see the electronic supplementary material to compare growth with the survival in figure 2).

Species survival also responded to plot-level conditions in different ways, so the most favourable location for one species could be one of the least favourable for another (follow the red line in figure 2). When species-specific responses to plot conditions were allowed, AIC and Bayesian information criterion both reduced by approximately 19, suggesting species truly respond differently to plot conditions.

**Figure 3.**
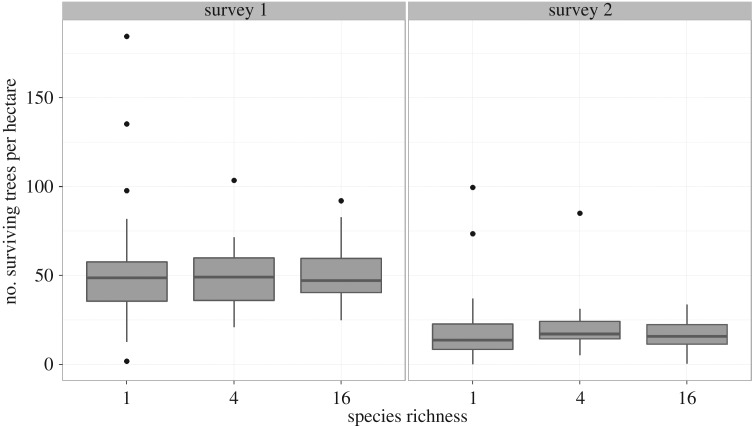
Density of surviving first-cohort seedlings as a function of plot species richness. The number of seedlings per ha within each plot, summarized with box and whiskers: boxes show the 25th, 50th and 75th percentile density, and whiskers extend to the most extreme density values within 1.5 times the interquartile range. While the median density remains constant, variation among plots decreases as species richness increases, particularly after two years (survey 1).

**Figure 4.**
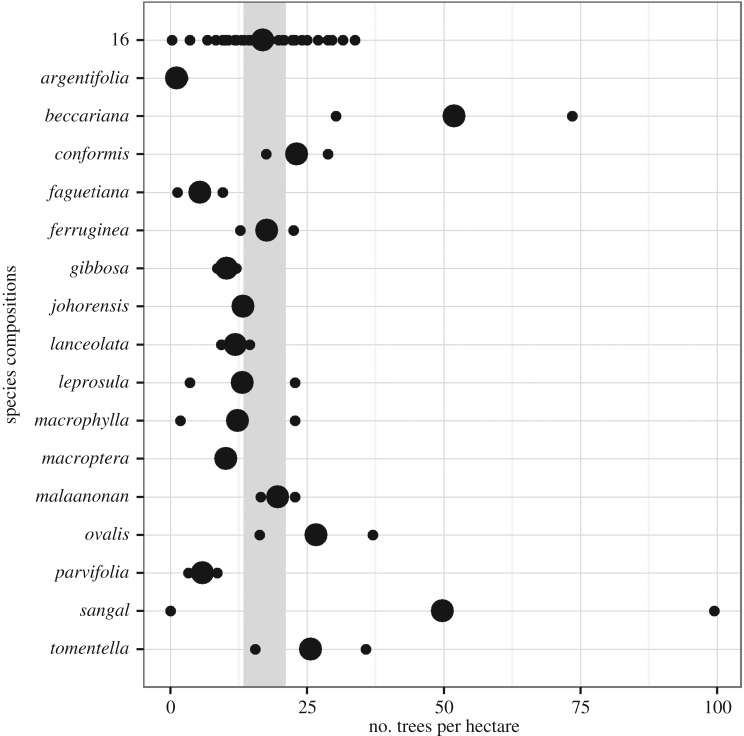
Density of surviving first-cohort seedlings after 10 years, in 16-species mixtures and monocultures. Small points show densities in each plot and large points the average. The grey band shows the 95% confidence interval of the 16-species mixture mean. The confidence interval for the probability of survival, *p*, was obtained using the Wilson method [48], then expressed as the number of surviving trees per ha, (*p*·*n*)/4. Many monocultures show extreme average densities compared with the 16-species mixture.

We cannot assess this insurance effect conclusively owing to the early stage of the experiment and the current lack of survival data for the second seedling cohort. However, at the first survey, the highest and lowest densities of surviving seedlings were seen in monocultures (figures 3 and 4). Out of 16 species, the average seedling density of only two species grown in monoculture fell within the 95% confidence interval (CI) for the average seedling density of the mixtures; the averages of nine monocultures fell below this 95% CI, and five monoculture averages fell above the 95% CI. The variability in density decreases as species richness increases, particularly after 2 years (figure 3). The replicated monocultures of a given species were often more variable than what we saw among the 16-species mixtures (figure 4). Planting more diverse mixtures did initially buffer the density of surviving seedlings after 2 years, but mortality continued over the following eight years and average density decreased within all species richness treatments. Whether there is a long-term insurance, effect of diversity on forest restoration will depend on the immediate and long-term survival of both seedling cohorts.

## 4. Discussion

Despite the early stage of the Sabah biodiversity experiment, several clear results emerge from our analysis of survival and growth during its initial decade. First, we found a clear life-history trade-off between survival and growth and consistent differences among our 16 dipterocarps in their positions along this trade-off during the two survey periods (figure 1). Second, not only did species differ on average, but they also responded differently to spatial variation, consistent with specialization on different conditions (figure 2). Third, as expected, given the wide spacing of the planted seedlings, there is no evidence of complementary species interactions in mixtures yet (figure 3). Finally, the most extreme high and low seedling densities are found in particular monocultures (figure 4). We discuss each of these points, in turn, before considering their relevance for enrichment planting schemes and the potential insurance effect of tree diversity in forest restoration.

### (a) The trade-off between survival and growth

The results of our more general analysis here support the conclusions of an earlier, more detailed analysis that identified a trade-off between growth and survival [26]. Our earlier work showed that these dipterocarps trade-off survival against growth generally, irrespective of the light conditions they are exposed to: all species were affected by light, but their ability to grow or survive relative to others remained unchanged. This follows work in other tree communities showing that the growth-survival trade-off is a major axis of life-history variation [37,49,50].

While species differences in survival rates were consistent over time in our study, species estimates of survival are not completely consistent with other studies. When comparing our survival estimates with those at the nearby INFAPRO enrichment-planting sites, the same species observed over a similar timescale experienced unrelated levels of mortality (E. Godoong *et al.* 2016, unpublished data). Among the species found in both our experiment and the INFAPRO site, those that have shown the best survival so far in our experiment have not been the best survivors at INFAPRO. For example, *D. lanceolata* was clearly the best survivor at INFAPRO after 13 years, with approximately 30% survival—twice the survival rate shown by any other species at the time. However, in our experiment, *S. ovalis*, also planted at INFAPRO, attained higher survival than *D. lanceolata*. These differences between our experiment and INFAPRO could be owing to numerous factors, including age of seedlings and site-specific conditions (see below).

### (b) Trait-mediated trade-offs

Various authors have hypothesized links between demographic rates and plant traits, in particular, wood density and specific leaf area [47,51,52], although some are more cautious [53–55]. Both the results of this analysis and of our earlier work support the link of the survival versus growth trade-off with wood density, such that species with denser wood have higher survival but lower growth rates. On the other hand, both our current analysis and earlier work found no association with specific leaf area. We did find that average survival of species were positively correlated with both total biomass and root mass ratio of the initial sample of harvested seedlings, as is often found [22]. Related experiments at the same site (with eight dipterocarps including seven of the species used here) have shown that individuals and species with higher levels of non-structural (soluble) carbohydrates survive longer under extreme drought—a major cause of tropical tree mortality that may be exacerbated by climate change [34,35]. Extending this work on non-structural carbohydrates and drought survival to the Sabah biodiversity experiment is one of the next priorities for the project.

### (c) Spatial heterogeneity and species-by-environment interactions

One strategy to improve enrichment planting survival rates may be to plant species in sites that will optimize their growth and survival based on their known ecology: species-site matching [21]. This strategy could be most easily implemented if species respond in differing ways to spatial heterogeneity at a relatively large-scale (coarse grain). It will require greater investment to implement if species respond to relatively fine-grained spatial heterogeneity. Spatial variation in mortality over the 500 ha Sabah biodiversity experiment site was substantial. Elevation is generally highest in the most northerly and southerly areas, decreasing towards the road separating the north and south blocks. There were no discernible effects of the road or the river (electronic supplementary material, figure S1). Within distances of 200 m or less (within plots), percentage survival commonly varied ±10% from average and more extreme fluctuations could be twice this in magnitude. Within plots, seedlings were planted with 3 m spacing along parallel lines 10 m apart. Survival tends to be more similar within lines than among them as would be expected given the shared conditions along lines (e.g. conditions when the line was planted; damage by elephants that use lines to move through the forest; canopy openness and light levels). These analyses show that species respond to site conditions differently and at a relatively fine scale which is supported by other studies in the region that relate seedling survival to microtopography and associated differences in soil moisture [56].

### (d) Lack of interactions between species in mixtures

As we expected, we found no evidence for an effect of plot species richness (or composition) on growth or survival. This is because there is limited scope for interactions between trees during the early stages of the experiment given their size relative to the planting density (pre-mortality) of 3 × 10 m. However, while the average seedling height to apical meristem in 2013 was only about 1 m (including the younger second seedling cohort, see the electronic supplementary material, Analysis) some of the larger survivors from the first cohort were already approaching sizes (12 m) where they may interact with neighbours, especially along planting lines. Regular measurement of survival and growth will allow us to detect when enrichment-planted seedlings start to strongly interact.

### (e) Enrichment planting

We found high mortality for the first seedling cohort, with only 35% remaining after 2 years and 12% after a decade. Rapid mortality is typical for enrichment planting—hence the replanting—but levels in our experiment are higher than some rates reported elsewhere [22] and for the nearby INFAPRO (approx. 50% at 3 years; Martijn Snoep and Yap Sau Wai) and Innoprise-IKEA Tropical Forest Rehabiliation Project (approx. 30–60% at 10 years). Intensive maintenance after planting improves survival rates [57] so it is possible some enrichment planting schemes may achieve better survival through this route. The state of the planted seedling stock also impacts survival and growth, so it will be interesting to compare the mortality reported here with the second cohort, which came from different stock. A new survey that includes measurement of the second cohort is therefore a priority for the project. One caveat when comparing our results with the wider literature is that our seedling densities are based strictly on the enrichment-planted seedlings, whereas other projects may inadvertently or deliberately also include naturally occurring seedlings.

### (f) Potential insurance effect of tree diversity in forest restoration

Owing to the small seedling size relative to the planting density, we knew there would be limited scope for interactions between species in mixtures during the initial stage of the experiment. However, we did anticipate that species differences in survival rates could provide the basis for an insurance effect of tree diversity, in which species mixtures avoid the potential recruitment failure of monocultures with low survival and relatively high rates of self-thinning in stands of species with the highest survival that could be potentially wasteful when seedling stocks are limited. Our results show how survival rates are variable and susceptible to spatial variation, which could generate such an insurance effect. After a decade, the lowest and highest seedling densities in the Sabah biodiversity experiment plots were observed in monocultures consistent with a potential insurance effect. However, it is too early to predict the eventual densities of different monocultures and mixtures, or what levels of self-thinning and recruitment failure will result. In comparison, the INFAPRO project's original goal was to reach a density of 15–30 mature harvestable (more than 60 cm dbh) dipterocarps per hectare to replace the trees that logging operations removed (the INFAPRO area has since been protected from commercial logging). The trade-off between survival and growth means that these two contributions to stem area tend to cancel out, producing some plots with a higher density of smaller trees and others with a lower density of larger trees. However, in the long term, we expect fast-growing species (lower survival and wood density) to be replaced by slow-growing species (that also tend to have higher wood density). Regular monitoring will be essential to identify the long-term role of tree diversity in the functioning of these ecosystems and its underlying biological mechanisms.

## 5. Conclusion

The fundamental mechanisms driving the biodiversity insurance hypothesis are the differing responses of species to environmental variation in space and time. These new results, and earlier findings from the Sabah biodiversity experiment, are consistent with the existence of such differences among the dipterocarps that dominate SE Asian tropical forests. These include a trade-off in which species with denser wood have lower growth rates but higher survival. Long-term monitoring as species interactions develop will reveal how important tree diversity is for restoring the structure and functioning of these forest ecosystems. Our expectation is that more species-rich replanting schemes will produce more consistent restorative effects throughout the forest landscape over the long term.

## Supplementary Material

Supplementary Figure 1 – Experiment map and history

## Supplementary Material

Supplementary Table 1 – Species descriptions

## Supplementary Material

Supplementary Table 2 – 4-species mixture compositions

## Supplementary Material

Supplementary Table 3 – Plot-by-plot composition treatments

## Supplementary Material

Supplementary Analysis Document

## Data accessibility

Data available from the Dryad Digital Repository: http://dx.doi.org/10.5061/dryad.1tc43 [58].
