# Supplementary material for: The value of biodiversity for the functioning of tropical forests: insurance effects during the first decade of the Sabah biodiversity experiment
Source: Proc Biol Sci. 2016 Dec 14;283(1844):20161451. doi: 10.1098/rspb.2016.1451 (PMC5204142; doi:10.1098/rspb.2016.1451)

Supplementary material Figure 1 – Experiment map and history.
(A) Layout of the Sabah Biodiversity Experiment showing the nearby river, the logging road, and the planting design. Assignment of experimental treatments to plots is not shown. (B) History of the experiment 2002-2013 showing the planting of the first seedling cohort (2002-3) and replanting with the second cohort (2008-11); the first (2003-5) and second census periods (2011-13); and extra surveys of subsets of plots (intensively sampled 16-species plots; plots with enhanced levels of climber cutting; and plots where seedlings and background forest were mapped using the FieldMap system).

A)

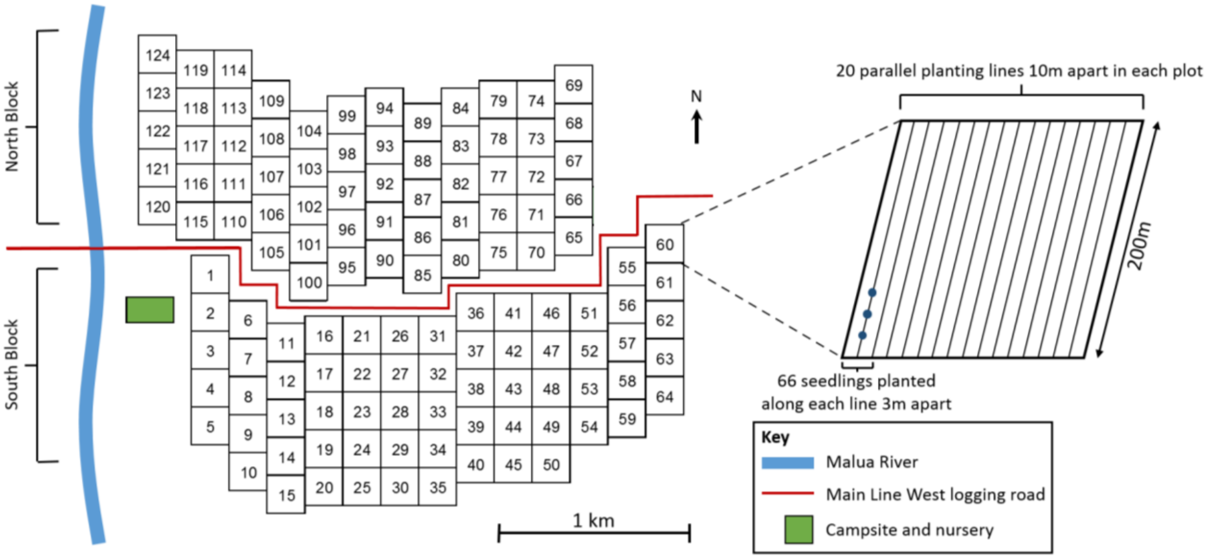

B)

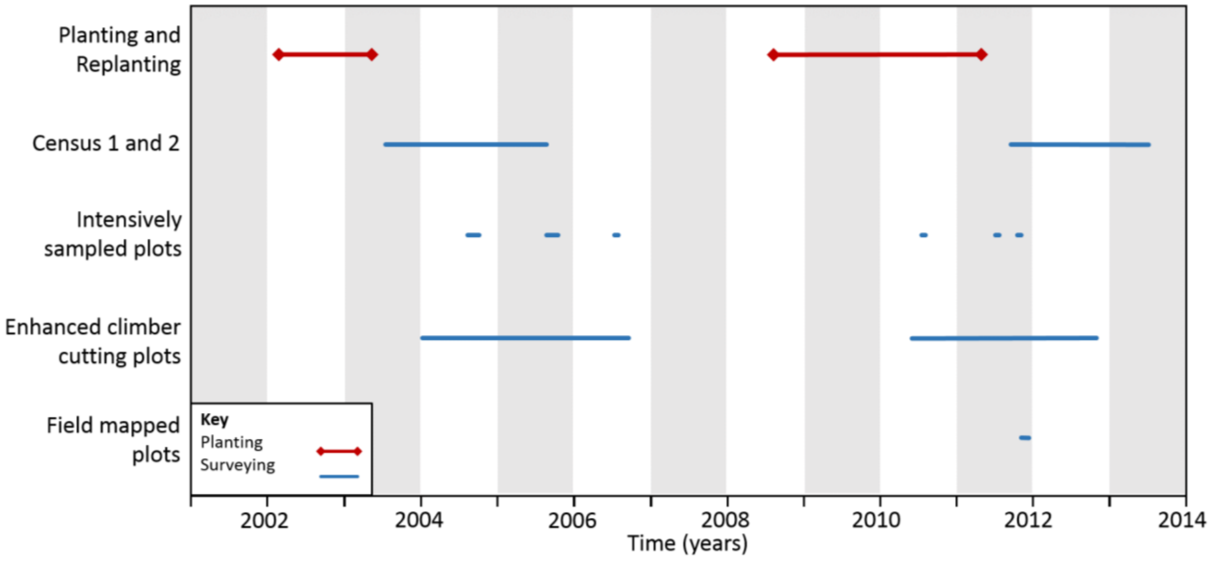

Supplement: Supplementary Figure 1 – Experiment map and history [file rspb20161451supp1.pdf]
