# Supplementary material for: The value of biodiversity for the functioning of tropical forests: insurance effects during the first decade of the Sabah biodiversity experiment
Source: Proc Biol Sci. 2016 Dec 14;283(1844):20161451. doi: 10.1098/rspb.2016.1451 (PMC5204142; doi:10.1098/rspb.2016.1451)
Supplement: Supplementary Table 1 – Species descriptions [file rspb20161451supp2.pdf]

**Supplementary material Table 1 – Species descriptions.**
Descriptions of the sixteen dipterocarp species planted in the Sabah Biodiversity Experiment: species names and authorities, taxonomic group (Ashton 1982; Kamiya et al. 2005), timber colour group (Symington 1943; Ashton 1982), ecology (Ashton 1982), and their IUCN Red List status (downloaded on 2nd December 2014 from www.iucnredlist.org).

| Species name | Species code | Taxonomic group | Timber group | Ecology | IUCN Red List |
|---|---|---|---|---|---|
| *Dipterocarpus conformis* Slooten | DC | Sister to Shoreae | – | Rare, hill dipterocarp forest, clay-rich soils, < 800 m | – |
| *Dryobalanops lanceolata* Burck | DL | Shoreae | – | Widespread on fertile soils, abundant on undulating land, to 700 m | Endangered |
| *Hopea ferruginea* Parij | HF | Shoreae | – | | Critically endangered |
| *Hopea sangal* Korth. | HS | Shoreae | – | Often on or near river banks in low country and to 500 m | Critically endangered |
| *Parashorea malaanonan* (Blanco) Merr. | PM | Shoreae | – | Local on clay-rich soil, rarely on riverbanks, on ridges in mountains to 1350 m | Critically endangered |
| *Parashorea tomentella* (Blanco) Merr. | PT | Shoreae | – | Common on flat to rolling hills < 200 m | – |
| *Shorea argentifolia* Sym. | SA | Shoreae > Shorea > Mutica sect. > Mutica subsect. | Red Meranti | Locally frequent on ridges, hillsides, and valleys, < 600 m | Endangered |
| *Shorea beccariana* Bruck | SB | Shoreae > Shorea > Pachycarpae sect. | Red Meranti | Common, lowlands, and dry ridges to 1350 m | – |
| *Shorea faguetiana* Heim. | SF | Shoreae > Shorea > Richetioides sect. > | Yellow Meranti | Low hills and particularly ridge tops at 150–700 m, occasionally to 1000 m | Endangered |

| | | | | | |
|---|---|---|---|---|---|
| | | Richetioides subsect. | | | |
| *Shorea gibbosa* Brandis. | SG | Shoreae > Shorea > Richetioides sect. | Yellow Meranti | Locally common on fertile clay-loam soils on undulating land and river banks to 650 m | Critically endangered |
| *Shorea johorensis* Foxw. | SJ | Shoreae > Shorea > Brachypterae sect. | Red Meranti | Very common on well-drained fertile soils < 600 m | Critically endangered |
| *Shorea leprosula* Miq. | SL | Shoreae > Shorea > Mutica sect. > Mutica subsect. | Red Meranti | Widespread, fast-growing emergent, common < 700 m | Endangered |
| *Shorea macrophylla* Ashton | SM1 | Shoreae > Shorea > Pachycarpae sect. | Red Meranti | Locally abundant on periodically flooded alluvium, rarer on hillsides, < 600 m | Vulnerable |
| *Shorea macroptera* King | SM2 | Shoreae > Shorea > Mutica sect. > Auriculatae subsect. | Red Meranti | Common, sandy clay soils on low hills to 600 m | – |
| *Shorea ovalis* Korth. | SO | Shoreae > Shorea > Ovalis sect. | Red Meranti | Scattered, usually in moist places in valleys and low-lying ground, occasionally ultrabasics, to 500 m | – |
| *Shorea parvifolia* Dyer | SP | Shoreae > Shorea > Mutica sect. > Mutica subsect. | Red Meranti | Perhaps the commonest dipterocarp, on clay soils on hills < 800 m | – |
