# Supplementary material for: The value of biodiversity for the functioning of tropical forests: insurance effects during the first decade of the Sabah biodiversity experiment
Source: Proc Biol Sci. 2016 Dec 14;283(1844):20161451. doi: 10.1098/rspb.2016.1451 (PMC5204142; doi:10.1098/rspb.2016.1451)
Supplement: Supplementary Table 2 – 4-species mixture compositions [file rspb20161451supp3.pdf]

Supplementary material Table 2 – 4-species mixture compositions

GENERIC DIVERSITY LOW (2 SPECIES)		THIN CANOPY		THICK CANOPY
	**4.1**	**Tall + medium I**	**4.5**	**Short + medium + tall I**
		Parashorea malaanonan (tall)		*Hopea sangal (short)*
		Parashorea tomentella (tall)		*Hopea ferruginea (short)*
		Shorea beccariana (medium)		*Shorea beccariana (medium)*
		Shorea leprosula (medium)		*Shorea johorensis (tall)*
	**4.2**	**Tall + medium II**	**4.6**	**Short + medium + tall II**
		Parashorea malaanonan (tall)		*Hopea sangal (short)*
		Parashorea tomentella (tall)		*Hopea ferruginea (short)*
		Shorea macroptera (medium)		*Shorea macroptera (medium)*
		Shorea ovalis (medium)		*Shorea gibbosa (tall)*
	**4.3**	**Short + medium I**	**4.7**	**Short + medium + tall III**
		Hopea sangal (short)		*Hopea sangal (short)*
		Hopea ferruginea (short)		*Hopea ferruginea (short)*
		Shorea macrophylla (medium)		*Shorea macrophylla (medium)*
		Shorea parvifolia (medium)		*Shorea faguetiana (tall)*
	**4.4**	**Short + medium II**	**4.8**	**Short + medium + tall IV**
		Hopea sangal (short)		*Hopea sangal (short)*
		Hopea ferruginea (short)		*Hopea ferruginea (short)*
		Shorea argentifolia (medium)		*Shorea argentifolia (medium)*
		Shorea parvifolia (medium)		*Shorea johorensis (tall)*

GENERIC DIVERSITY HIGH (4 SPECIES)		THIN CANOPY		THICK CANOPY
	**4.9**	**Tall I**	**4.13**	**Tall + medium + Short I**
		Dipterocarpus conformis (tall)		*Dipterocarpus conformis (tall)*
		Dryobalanops lanceolata (tall)		*Dryobalanops lanceolata (tall)*
		Parashorea malaanonan (tall)		*Shorea macrophylla (medium)*
		Shorea faguetiana (tall)		*Hopea sangal (short)*
	**4.10**	**Tall II**	**4.14**	**Tall + medium + Short II**
		Dipterocarpus conformis (tall)		*Dipterocarpus conformis (tall)*
		Dryobalanops lanceolata (tall)		*Dryobalanops lanceolata (tall)*
		Parashorea tomentella (tall)		*Shorea ovalis (medium)*
		Shorea johorensis (tall)		*Hopea ferruginea (short)*
	**4.11**	**Tall II**	**4.15**	**Tall + medium + Short III**
		Dipterocarpus conformis (tall)		*Dipterocarpus conformis (tall)*
		Dryobalanops lanceolata (tall)		*Dryobalanops lanceolata (tall)*
		Parashorea tomentella (tall)		*Shorea ovalis (medium)*
		Shorea gibbosa (tall)		*Hopea sangal (short)*
	**4.12**	**Tall + medium III**	**4.16**	**Tall + medium + Short IV**
		Dipterocarpus conformis (tall)		*Dipterocarpus conformis (tall)*
		Dryobalanops lanceolata (tall)		*Dryobalanops lanceolata (tall)*
		Parashorea malaanonan (tall)		*Shorea macrophylla (medium)*
		Shorea johorensis (tall)		*Hopea ferruginea (short)*
