# Supplementary material for: The value of biodiversity for the functioning of tropical forests: insurance effects during the first decade of the Sabah biodiversity experiment
Source: Proc Biol Sci. 2016 Dec 14;283(1844):20161451. doi: 10.1098/rspb.2016.1451 (PMC5204142; doi:10.1098/rspb.2016.1451)
Supplement: Supplementary Table 3 – Plot-by-plot composition treatments [file rspb20161451supp4.pdf]

Supplementary material Table 3 – Plot-by-plot composition treatments

Block	Plot	Species richness	Species composition
South	1	4	4.13
South	2	1	*Shorea beccariana*
South	3	16	Climber cutting
South	4	1	*Hopea sangal*
South	5	16	16
South	6	4	4.6
South	7	1	*Shorea leprosula*
South	8	16	16
South	9	4	4.3
South	10	1	*Dryobalanops lanceolata*
South	11	16	16
South	12	4	4.10
South	13	1	*Shorea gibbosa*
South	14	16	16
South	15	16	Climber cutting
South	16	4	4.11
South	17	16	16
South	18	1	*Parashorea tomentella*
South	19	4	4.5
South	20	NA	Control (unplanted)
South	21	16	16
South	22	16	Climber cutting
South	23	1	*Shorea parvifolia*
South	24	NA	Control (unplanted)
South	25	4	4.2
South	26	NA	Control (unplanted)
South	27	1	*Shorea macrophylla*
South	28	4	4.7
South	29	16	16
South	30	16	Climber cutting
South	31	16	Climber cutting
South	32	16	16
South	33	16	Climber cutting

South	34	1	*Shorea argentifolia*
South	35	4	4.12
South	36	NA	Control (unplanted)
South	37	1	*Shorea ovalis*
South	38	16	16
South	39	4	4.9
South	40	16	Climber cutting
South	41	4	4.14
South	42	16	Climber cutting
South	43	1	*Shorea faguetiana*
South	44	1	*Parashorea malaanonan*
South	45	16	16
South	46	16	16
South	47	NA	Control (unplanted)
South	48	4	4.16
South	49	16	16
South	50	1	*Shorea macroptera*
South	51	16	Climber cutting
South	52	1	*Dipterocarpus conformis*
South	53	16	16
South	54	4	4.8
South	55	16	16
South	56	4	4.4
South	57	NA	Control (unplanted)
South	58	4	4.1
South	59	1	*Shorea johorensis*
South	60	4	4.15
South	61	1	*Hopea ferruginea*
South	62	16	Climber cutting
South	63	16	16
South	64	16	16
North	65	1	*Shorea parvifolia*
North	66	16	16
North	67	4	4.2
North	68	NA	Control (unplanted)
North	69	1	*Hopea ferruginea*

North	70	1	*Parashorea malaanonan*
North	71	4	4.8
North	72	16	Climber cutting
North	73	16	16
North	74	4	4.9
North	75	16	Climber cutting
North	76	16	16
North	77	4	4.5
North	78	1	*Shorea faguetiana*
North	79	4	4.7
North	80	NA	Control (unplanted)
North	81	1	*Shorea macrophylla*
North	82	4	4.4
North	83	16	16
North	84	1	*Shorea beccariana*
North	85	16	16
North	86	1	*Dipterocarpus conformis*
North	87	16	16
North	88	4	4.6
North	89	NA	Control (unplanted)
North	90	1	*Shorea argentifolia*
North	91	16	16
North	92	16	Climber cutting
North	93	4	4.10
North	94	16	16
North	95	NA	Control (unplanted)
North	96	16	16
North	97	4	4.14
North	98	1	*Parashorea tomentella*
North	99	16	16
North	100	16	Climber cutting
North	101	1	*Shorea ovalis*
North	102	4	4.11
North	103	16	16
North	104	1	*Dryobalanops lanceolata*
North	105	4	4.13

North	106	16	16
North	107	1	*Shorea gibbosa*
North	108	NA	Control (unplanted)
North	109	4	4.1
North	110	1	*Shorea macroptera*
North	111	16	Climber cutting
North	112	4	4.16
North	113	16	16
North	114	1	*Shorea leprosula*
North	115	16	16
North	116	4	4.12
North	117	NA	Control (unplanted)
North	118	1	*Hopea sangal*
North	119	4	4.3
North	120	16	16
North	121	1	*Shorea johorensis*
North	122	4	4.15
North	123	16	Climber cutting
North	124	16	16
