# Supplementary material for: The value of biodiversity for the functioning of tropical forests: insurance effects during the first decade of the Sabah biodiversity experiment
Source: Proc Biol Sci. 2016 Dec 14;283(1844):20161451. doi: 10.1098/rspb.2016.1451 (PMC5204142; doi:10.1098/rspb.2016.1451)
Supplement: Supplementary Analysis Document [file rspb20161451supp5.pdf]

Insurance Effects Of Tree Diversity In Tropical Forest Restoration: Survival And Growth During The First Decade Of The Sabah Biodiversity Experiment
Supplementary Material - Analysis

Sean Tuck

September 13, 2016

Contents

1 Introduction **2**

1.1 Reproducible research with dynamic documents 3

1.2 Experimental design 3

1.3 Approach to the analysis 4

1.3.1 General statistical approach 4

1.3.2 Statistical considerations 5

1.3.3 Analysis plan 6

2 Read and check data **6**

2.1 Initial checks 10

3 Aggregate the data **12**

3.1 Aggregate survival data 12

3.2 Aggregate growth data 13

4 Exploratory plotting **15**

4.1 Explore survival data 17

4.1.1 Seedling ages 17

4.1.2 Survival response to richness 18

4.1.3 Survival response to composition 19

4.2 Explore growth data 22

4.2.1 Survey times 22

4.2.2 Growth response to composition 23

5 Modelling **24**

5.1 Fit the model for survival 24

5.2 Survival residual diagnostics . . . 34
5.3 Fit the model for growth . . . 39
5.3.1 Model fit plot . . . 45
5.4 Growth residual diagnostics . . . 45
5.5 Models inference . . . 50

6 Extract information from models **55**
6.1 Extract growth slope values . . . 55
6.2 Extract survival fixed effects . . . 57
6.3 Collate information into ideal data.frame for plots . . . 59

7 Further plotting **68**
7.1 Correlation between growth slopes and intercepts . . . 68
7.2 Species responses to composition treatments . . . 68

8 Manuscript figures **71**
8.1 Figure 2 . . . 71
8.2 Main text results: correlations with traits . . . 78
8.3 Figure 3 . . . 79
8.3.1 Figure 3a . . . 80
8.3.2 Figure 3b Supplementary . . . 82
8.4 Figure 4 . . . 83
8.4.1 Figure 4a . . . 84
8.4.2 Figure 4b . . . 86
8.5 Figure 5 . . . 87

9 Supplementary figures **89**

1 Introduction

This document accompanies a research article as supplementary material. We will begin by describing the utility of 'dynamic documents' such as this for making research open and reproducible. We will also explain the logic behind our analysis and the choices we made when analysing the data. In the rest of the document, we will present the entire analysis for this research project.

In our research article, we analysed the mortality and growth of dipterocarp tree seedlings that were planted in the Sabah Biodiversity Experiment in Sabah, Malaysia. We analysed mortality and growth over the first decade of this long-term experiment. We explored whether species differences in mortality and growth also depended on environmental variation. If so, mixtures of species may be less variable across the whole experimental site, because the performances of different species are averaged. In ecology, related hypotheses that more diverse mixtures of species are less variable over space or time due to differences between species are called the 'portfolio effect' and the 'insurance effect'. These effects have been demonstrated in previous experiments in grasslands, and more rarely in forest ecosystems.

We present the first experiment exploring these effects in palaeotropic forest ecosystems.

1.1 Reproducible research with dynamic documents

This document contains every step taken during our analysis so the reader can see exactly how we arrived at the results presented in our research article. It is a 'dynamic document', which contains the code used to handle, process and analyse the data. Displayed alongside each section of code is the output produced when that code is evaluated. As a result the document contains our entire workflow, from data to results, so that others can fully repeat our methodology and reproduce our results. The dataset is also openly accessible so that these results can be reproduced. The source code used to produce this PDF document is also available, so that the analysis code is directly accessible.

There are several advantages to this approach compared with, for example, released an R script instead. Firstly, we are able to embed text alongside each section of code and accompanying graphs, etc. This allows us to explain an details and reasoning in a clear and relevant way. By producing all results alongisde the code, the PDF also shows anybody who wishes to reproduce our analysis exactly what they should expect to see. A dynamic document approach also helps ensure that our work is reproducible in the long-term as well, because we can clearly show important details such as software and package versions that we used. This document was produced using the knitr R package for dynamic report generation. For more information on the utility of this approach, see the knitr website or book.

1.2 Experimental design

Our dataset has a complex spatial and temporal structure. The experiment is sited within 500 hectares of selectively logged tropical forest. Our experimental treatments (planted trees) are imposed into the background forest. Spatially, the basic experimental units (individual trees) are planted along lines, within 125 four-hectare (200 x 200 m) plots, that are grouped into two replicate blocks. A map of the experiment is separately provided as supplementary material for the research article. The lines that the experimental trees are planted along run north-south through the experiment (either side of a west-east road). The lines were cut into the background forest when the experiment was set up. They were cut continuously through the landscape, then the plot treatments were introduced along these lines as shown on the map. Treatments were randomly assigned to plots within each block (hence the design is a randomised block design). The planting lines are spaced 10 metres apart, giving room for 20 planting lines within each plot. Individual trees are planted 3 metres apart along each planting line.

Plots have either 1, 4 or 16 species planted in them (each diversity level having 16 x 2 replicates), producing a dataset of X length. The 16-species mixtures all contain the same 16 species, and these species are all represented in the 16 monoculture plots. The 16 different 4-species mixtures are combinations that together include all species, in combinations that produce varying levels of generic diversity and expected mature-tree canopy structures. The species compositions of these 4-species mixtures are shown below and in a separate supplementary material for the research article. As explained above, there are 32 (16 x 2 replicates) plots within each of the three diversity levels. These 96 plots comprise the diversity gradient plots within the experiment. Twelves other plots are control plots, where the experimental design was imposed without any planting treatment. Another 16 plots were used for an enhanced climber cutting treatment: 16-species mixtures were planted again, but with climber/liana cutting maintenance throughout the entire plot (including between lines), instead of only cutting climbers along the planting lines. One final plot was used for training field surveyors, bringing the total number of plots to 125. The 96 diversity gradient plots are the only ones analysed in the research article.

At the tree level our primary response variables are survival (binary) and growth (continuous, log-

transformed size) but for the analyses here we aggregated the data to produce average survival and size values for each species within each plot (see subsection 1.3).

Trees were planted in the experiment firstly between 2002 and 2003. A second cohort of trees were planted between 2009 and 2011, but these have not yet been sufficiently surveyed to be analysed in the research article. The first cohort of trees have been fully surveyed twice: firstly from November 2003 to May 2005, and then from November 2011 to September 2013. Six of the 16-species mixture plots were sampled more intensively to supplement the whole experiments more rigorous but less frequent sampling (plots 5, 8, 11, 14 and 17, plus plot 3 which received enhanced climber cutting treatment). This intensive sampling was analysed in a previous publication, by Philipson et al. (2014). In this research article we analysed the two full surveys of the diversity gradient plots. During sampling, plots were surveyed sequentially from west to east across the site, firstly in the south block and then the north block - this follows the order in which trees were planted. There is no problem with the ordering of plot sampling because the treatments were randomly assigned to plots within each block. Because it takes roughly two years to survey the entire experiment, the ages of trees when surveyed do vary (trees at the first survey are mostly between 1.6 and 2.2 years old, with a minority of trees revisited later - see subsubsection 4.1.1). Despite the variation in age, observations clearly group into two different surveys (see subsubsection 4.2.1). Coupled with the fact that plots were assigned randomly, this means that differences in survey times will not add much noise to our analysis. To find out how we dealt with time and repeated measures of trees in our analysis, see subsection 1.3 below.

The experiment site is situated in Malua Forest Reserve, which was logged between 1984 and 1986. After the experiment was set up, the surrounding forest in the reserve was logged again in 2007. The logging in the 1980s introduced greater heterogeneity in the landscape than there would otherwise have been. The initial conditions are therefore highly variable across the whole site. Some areas were highly degraded by logging activity, leaving high bamboo coverage and relatively little canopy cover. Other areas are relatively untouched, with some large trees remaining. We therefore expect spatial variation to have a strong impact upon our observed effects. As a result, it is possible that species-specific responses to this variation may only be detectable if they are strong effects (relative to the overall effect of spatial variation). This variation can occur within 200 metres - i.e. at within-plot scale - whereas the effect of initial conditions at more distant locations are not likely to be as closely related. For details of how we deal with this spatial correlation, see subsection 1.3.

1.3 Approach to the analysis

1.3.1 General statistical approach

There are an ever growing number of critiques of statistical analysis and inference used within scientific research. It is widely considered among statisticians that scientists focus too much on p-values without paying enough attention to effect sizes. The challenges to the view that all results should be formalised with null hypothesis significance tests take several points. The common interpretation of p-values has been challenged, especially when multiple comparisons have been made on a post hoc basis. Instead, a rule of thumb could be to consider p-values only when presenting a test of a meaningful hypothesis that was devised prior to data collection and analysis. It is best that the data also come from a controlled experiment. Researcher degrees of freedom - the garden of forking paths associated with all decisions made after seeing the data, including aggregating data and model building - represents even more complicated challenges to the interpretation of null hypothesis significance tests.

In an effort to tackle these issues, a more graphical and estimation-based approach to statistics is often recommended nowadays. This approach allows researchers to focus on the effect sizes of interest and present insights from data in a clear, relevant way. The evidence can be presented without making extra assumptions and concluding the strength of evidence based on arbitrary decision thresholds. Much controversy still exists on these issues, but many statisticians agree that traditional use of p-values and null hypothesis significance testing is problematic - the most high-profile demonstration of

this was perhaps the report published by the American Statistical Association. We aim to follow the advice of this report and prominent statisticians such as Andrew Gelman. We also take every effort to follow the advice of those who wrote the statistical software that we use, such as Doug Bates.

This graphical analysis approach is particularly well suited to our project for various reasons. As explained above, the experimental design and context is complicated. Part of this context is that some elements of the experiment are deliberately not controlled, as this experiment aims to connect evidence in controlled experiments with real-world ecosystems at the landscape level. Furthermore, the analyses presented in our manuscript were not the result of hypotheses devised prior to a decade's worth of data collection. There were also several statistical considerations that led us to average groups and change the model we fitted to the data. Where absolutely necessary we have utilised the 'duality of testing', and formalised graphical analyses with confidence intervals rather than p-values. But the approach was always to fit the most appropriate models given our questions and situation, to use models that made comparing survival and growth results easy, and then to visualise the results in the most informative way possible.

1.3.2 Statistical considerations

As the experiment is so large and conditions vary across the landscape (in terms of logging effects and environmental conditions), some locations will be more similar than others, particularly if they are nearby locations. This means that the growth and survival of otherwise similar seedlings are likely to be correlated when closer to each other, as the effects on growth and survival that spatially vary will be more similar. As a result, observations are spatially correlated, violating the assumption that observations are identical and independent draws from the same distribution. Spatial correlation, autocorrelation and non-independence are synonyms - I have referred to spatial correlation here as this may be the most familiar terminology to most readers.

The scale of spatial correlation is within 200 metres or less. This was identified by looking at variograms, as is most often suggested in modern statistical books (e.g. Pinheiro and Bates 2000, Mixed Effects Models in S and S-PLUS). We dealt with this spatial correlation as explained below, and following variograms show the issue to be solved (see subsection 5.2 and subsection 5.4). There are several ways to deal with spatial correlation. If you have information about the variables that spatially vary at the correct scale, you can add those variables in your model and thereby remove that spatial structure from the residuals. We do not currently have any such variables that vary at the seedling level, which can explain within-plot variation. Another option is to specify a correlation structure in the error distribution of the model, like a generalised least squares model. Modelling correlated errors substantially increases the complexity of the model and therefore increases the difficulty in fitting and interpreting the model. Unless specifically interested in this correlation structure, this approach is not the best. Another option available is to aggregate non-independent observations, thereby removing the spatial correlation at the original scale of observation. As long as you have no predictors that vary at the original scale (i.e. the seedling level), you can aggregate grouped data without losing any information and get the same results. This was the case for us, so we chose to aggregate data for each species within the same plot. So, in 16-species mixture plots, we had an average survival and size measurement for each of the 16 species. In a monoculture plot, there is just one average survival and size measurement for that species.

Aggregating the data reduces our sample size, but it also increases the precision of our data. The result is that we do not lose any statistical power. Aggregating also dealt with a fundamental problem we had with fitting models at the seedling level. The survival model would not converge because there were cases when all survival observations within some groups (at the planting-line-within-plot level) were either dead or alive; all 0 or 1 observations pushed the estimation process to the boundary of the parameter space ($\pm$infinity), causing model convergence failure. There were no such cases among all individuals for one species within a single plot, so aggregating survival data to this level solved the problem. We aggregated tree size data to the same level so that the two models were comparable; this

did not cost us anything, because we had no predictors that varied at the seedling level.

Aggregating the data also allowed us to simplify the models in several ways. Firstly, the random effects structure was simplified because we had averaged away the planting line design level. Secondly, because of the central limit theorem, our average survival data were approximately normally distributed. We could therefore fit a gaussian linear model to the survival data without causing any distributional assumption problems. We could fit a Binomial model to the aggregated survival data, but there was no advantage in doing so. The Binomial model would not give us any extra information, but it would more difficult to interpret and perform rigorous residual diagnostics. The Binomial model would also be less comparable to the growth model, which was modelled with a normal error distribution (the response variable for the growth model was log-transformed tree diameter - the slopes over time estimated relative growth rate). For these reasons, we opted to model survival using a normal error distribution as well. As our statistical approach does not focus on null hypothesis statistical inference, these issues are even less important - but we check model assumptions anyway for rigorousness.

Another consideration for our models was that we have repeated measures of survival and size for the same trees. This is not a problem for our analysis, providing we model the data appropriately. Each observation represents all trees of one species in one plot pooled and measured at one time point. To account for the fact that the pooled trees in each plot were measured twice, a random effect (species:plot) was included in the analysis. In addition, time is included in the models and is considered to be a fixed effect (for survival, a factor with two levels; for growth, a slope). Thus, there is no independence assumption violated. This is a typical repeated measures design. A classic demonstration of this standard practice would be in a medical trial, where each patient gets drugA and drugB at different times. In this model, each patient is measured more than once and therefore a random effect to group patient observations is needed.

1.3.3 Analysis plan

This analysis will i) estimate species average survival after two surveys (two years and ten years since planting), ii) quantify the effects of species composition experimental treatments on mortality for each species, iii) describe the magnitude of spatial variation among species and whether species respond to plot-level variation in different ways, and iv) present evidence for an insurance effect of increasing species richness on seedling mortality.

This analysis will produce 4 manuscript figures:

1. Species-level mortality vs. growth estimates, also showing relationship between the two.
2. Magnitude of plot-level spatial variation in mortality and growth for each species, with species-dependence.
3. Spatial insurance effect of richness on mortality and growth.
4. Comparison between the full species mixture and each constituent monoculture.

2 Read and check data

Read in necessary functions and data, and check the data are coded correctly.

```
require(lme4)
require(ggplot2)
require(doBy)
require(RColorBrewer)
require(reshape2)
```

```r
require(knitr)
require(lattice)
require(regr0)
require(stringr)
require(binom)
require(geoR)
require(gamm4)
require(SBEdata)
```

```r
sessionInfo()
```

```
R version 3.3.1 (2016-06-21)
Platform: x86_64-apple-darwin13.4.0 (64-bit)
Running under: OS X 10.11.6 (El Capitan)

locale:
[1] en_GB.UTF-8/en_GB.UTF-8/en_GB.UTF-8/C/en_GB.UTF-8/en_GB.UTF-8

attached base packages:
[1] stats     graphics  grDevices utils     datasets  methods   base

other attached packages:
 [1] SBEdata_0.2.1      gamm4_0.2-3        mgcv_1.8-12
 [4] nlme_3.1-128       geoR_1.7-5.2       binom_1.1-1
 [7] stringr_1.1.0      regr0_1.0-5        lattice_0.20-33
[10] reshape2_1.4.1     RColorBrewer_1.1-2 doBy_4.5-15
[13] ggplot2_2.1.0      lme4_1.1-12        Matrix_1.2-6
[16] knitr_1.14

loaded via a namespace (and not attached):
 [1] Rcpp_0.12.6               DEoptimR_1.0-6
 [3] formatR_1.4               nloptr_1.0.4
 [5] plyr_1.8.4                highr_0.6
 [7] tools_3.3.1               digest_0.6.9
 [9] evaluate_0.9              gtable_0.2.0
[11] SparseM_1.7               MatrixModels_0.4-1
[13] RandomFieldsUtils_0.2.1.1 grid_3.3.1
[15] nnet_7.3-12               robustbase_0.92-6
[17] tcltk_3.3.1               survival_2.39-4
[19] sp_1.2-3                  minqa_1.2.4
[21] magrittr_1.5              codetools_0.2-14
[23] scales_0.4.0              splancs_2.01-39
[25] RandomFields_3.1.16       MASS_7.3-45
[27] splines_3.3.1             colorspace_1.2-6
[29] quantreg_5.26             stringi_1.1.1
[31] munsell_0.4.3
```

```r
source("~/Dropbox/Scripts/fun_diagnostics.R")
#source("~/Dropbox/Scripts/fmyColRamp.R")
msSurv <- readRDS(
  paste0("~/Dropbox/PhD/_SBE/SBE_Project/Data/",
```

```
        "SBE_GrowthData2014_Dbox/manuscriptData_Survival.rds"))
msGrowth <- readRDS(
  paste0("~/Dropbox/PhD/_SBE/SBE_Project/Data/",
         "SBE_GrowthData2014_Dbox/manuscriptData_Growth.rds"))

showd(msSurv)

dim:  106922 23
          tree.id bl  pl li  po    X    Y sd fd gd       sp.comp
1          1.1.10  1   1  1  10   10    3  4  3  4          4.13
2          1.1.10  1   1  1  10   10    3  4  3  4          4.13
3          1.1.20  1   1  1  20   10    6  4  3  4          4.13
...
29723   17.19.120  1  17 19 120  790 -444 16  3  5       sixteen
63338    58.1.350  1  58  1 350 2210 -455  4  2  2           4.1
93750   90.18.180  2  90 18 180 1100  154  1  1  1 argentifolia
124744 124.20.600  2 124 20 600  -80 1340 16  3  5       sixteen
       planting.date       genus           sp pl.li pl.row survey.type
1         2002-07-18 Dryobalanops   lanceolata   1.1    1.1     census1
2         2002-07-18 Dryobalanops   lanceolata   1.1    1.1     census2
3         2002-07-18 Dryobalanops   lanceolata   1.1    1.2     census1
...
29723     2002-08-21       Shorea   beccariana 17.19  17.12     census2
63338     2002-11-08       Shorea    leprosula  58.1  58.35     census1
93750     2003-08-24       Shorea argentifolia 90.18  90.18     census1
124744    2003-01-10       Shorea argentifolia 124.2  124.6     census2
       survey.date survival d.base dbh yearsSincePlanting
1       2003-11-18        1      8  NA                1.3
2       2011-11-25        1     82  64                9.4
3       2003-11-18        1      9  NA                1.3
...
29723   2012-08-28        0     NA  NA               10.0
63338   2005-01-24        0     NA  NA                2.2
93750   2005-06-27        0     NA  NA                1.8
124744  2013-09-23        0     NA  NA               10.7
       daysSincePlanting
1               488 days
2              3417 days
3               488 days
...
29723          3660 days
63338           808 days
93750           673 days
124744         3909 days

str(msSurv)

'data.frame': 106922 obs. of  23 variables:
 $ tree.id       : Factor w/ 53841 levels "1.1.100","1.1.110",..: 11 11 22 22 33 33 44 44 46 46 ...
 $ bl                : Factor w/ 2 levels "1","2": 1 1 1 1 1 1 1 1 1 1 ...
 $ pl               : Factor w/ 112 levels "1","2","3","4",..: 1 1 1 1 1 1 1 1 1 1 ...
 $ li               : Factor w/ 20 levels "1","2","3","4",..: 1 1 1 1 1 1 1 1 1 1 ...
```

```
$ po          : Factor w/ 128 levels "10N","10O","11N",..: 22 22 44 44 66 66 88 88 110 110 ...
 $ X                  : int  10 10 10 10 10 10 10 10 10 10 ...
 $ Y                  : int  3 3 6 6 9 9 12 12 15 15 ...
 $ sd                 : Factor w/ 3 levels "1","4","16": 2 2 2 2 2 2 2 2 2 2 ...
 $ fd                 : Factor w/ 3 levels "1","2","3": 3 3 3 3 3 3 3 3 3 3 ...
 $ gd                 : Factor w/ 4 levels "1","2","4","5": 3 3 3 3 3 3 3 3 3 3 ...
 $ sp.comp         : Factor w/ 33 levels "4.1","4.10","4.11",..: 5 5 5 5 5 5 5 5 5 5 ...
 $ planting.date    : Factor w/ 112 levels "2002-01-27","2002-02-02",..: 9 9 9 9 9 9 9 9 9 9 ...
 $ genus              : Factor w/ 5 levels "Dipterocarpus",..: 2 2 2 2 5 5 5 5 1 1 ...
 $ sp              : Factor w/ 16 levels "argentifolia",..: 8 8 8 8 10 10 10 10 3 3 ...
 $ pl.li          : Factor w/ 1728 levels "1.1","1.11","1.12",..: 1 1 1 1 1 1 1 1 1 1 ...
 $ pl.row        : Factor w/ 5720 levels "1.1","1.11","1.12",..: 1 1 11 11 21 21 31 31 41 41 ...
 $ survey.type       : Factor w/ 2 levels "census1","census2": 1 2 1 2 1 2 1 2 1 2 ...
 $ survey.date     : Factor w/ 366 levels "2003-11-18","2003-11-19",..: 1 210 1 210 1 210 1 210 202
 $ survival           : int  1 1 1 1 1 1 1 1 0 0 ...
 $ d.base             : num  7.99 82 9.03 141 14.33 ...
 $ dbh                : num  NA 64 NA 111 2.53 210 NA 87 NA NA ...
 $ yearsSincePlanting: num  1.34 9.36 1.34 9.36 1.34 ...
 $ daysSincePlanting :Class 'difftime'  atomic [1:106922] 488 3417 488 3417 488 ...
  .. ..- attr(*, "units")= chr "days"


showd(msGrowth)


dim:  12320 23
          tree.id bl  pl li  po    X    Y sd fd gd sp.comp planting.date
1          1.1.10  1   1  1  10   10    3  4  3  4    4.13    2002-07-18
2          1.1.10  1   1  1  10   10    3  4  3  4    4.13    2002-07-18
3          1.1.20  1   1  1  20   10    6  4  3  4    4.13    2002-07-18
...
20115    11.20.70  1  11 20  70  600 -339 16  3  5 sixteen    2002-08-04
54185    45.13.80  1  45 13  80 1730 -976 16  3  5 sixteen    2002-11-05
83782    79.13.80  2  79 13  80 1650  957  4  3  2     4.7    2003-08-20
124710 124.20.220  2 124 20 220  -80 1226 16  3  5 sixteen    2003-01-10
              genus          sp pl.li pl.row survey.type survey.date
1      Dryobalanops lanceolata   1.1    1.1     census1  2003-11-18
2      Dryobalanops lanceolata   1.1    1.1     census2  2011-11-25
3      Dryobalanops lanceolata   1.1    1.2     census1  2003-11-18
...
20115         Shorea beccariana  11.2   11.7     census2  2012-07-28
54185    Parashorea tomentella 45.13   45.8     census2  2012-11-23
83782          Hopea      sangal 79.13   79.8     census1  2006-01-18
124710        Shorea macroptera 124.2 124.22     census2  2013-09-24
       survival d.base  dbh yearsSincePlanting daysSincePlanting
1             1    8.0   NA                1.3          488 days
2             1   82.0 64.0                9.4         3417 days
3             1    9.0   NA                1.3          488 days
...
20115         1   83.0 69.0               10.0         3646 days
54185         1   14.5  6.2               10.1         3671 days
83782         1    5.1   NA                2.4          882 days
124710        1   12.5   NA               10.7         3910 days
```

```
str(msGrowth)

'data.frame': 12320 obs. of  23 variables:
 $ tree.id          : Factor w/ 53841 levels "1.1.100","1.1.110",..: 11 11 22 22 33 33 44 44 1 1 ...
 $ bl               : Factor w/ 2 levels "1","2": 1 1 1 1 1 1 1 1 1 1 ...
 $ pl               : Factor w/ 112 levels "1","2","3","4",..: 1 1 1 1 1 1 1 1 1 1 ...
 $ li               : Factor w/ 20 levels "1","2","3","4",..: 1 1 1 1 1 1 1 1 1 1 ...
 $ po               : Factor w/ 128 levels "10N","100","11N",..: 22 22 44 44 66 66 88 88 2 2 ...
 $ X                : int  10 10 10 10 10 10 10 10 10 10 ...
 $ Y                : int  3 3 6 6 9 9 12 12 30 30 ...
 $ sd               : Factor w/ 3 levels "1","4","16": 2 2 2 2 2 2 2 2 2 2 ...
 $ fd               : Factor w/ 3 levels "1","2","3": 3 3 3 3 3 3 3 3 3 3 ...
 $ gd               : Factor w/ 4 levels "1","2","4","5": 3 3 3 3 3 3 3 3 3 3 ...
 $ sp.comp          : Factor w/ 33 levels "4.1","4.10","4.11",..: 5 5 5 5 5 5 5 5 5 5 ...
 $ planting.date    : Factor w/ 112 levels "2002-01-27","2002-02-02",..: 9 9 9 9 9 9 9 9 9 9 ...
 $ genus            : Factor w/ 5 levels "Dipterocarpus",..: 2 2 2 2 5 5 5 5 1 1 ...
 $ sp               : Factor w/ 16 levels "argentifolia",..: 8 8 8 8 10 10 10 10 3 3 ...
 $ pl.li            : Factor w/ 1728 levels "1.1","1.11","1.12",..: 1 1 1 1 1 1 1 1 1 1 ...
 $ pl.row           : Factor w/ 5720 levels "1.1","1.11","1.12",..: 1 1 11 11 21 21 31 31 1 1 ...
 $ survey.type      : Factor w/ 2 levels "census1","census2": 1 2 1 2 1 2 1 2 1 2 ...
 $ survey.date      : Factor w/ 366 levels "2003-11-18","2003-11-19",..: 1 210 1 210 1 210 1 210 1 21
 $ survival         : int  1 1 1 1 1 1 1 1 1 1 ...
 $ d.base           : num  7.99 82 9.03 141 14.33 ...
 $ dbh              : num  NA 64 NA 111 2.53 210 NA 87 NA 44.1 ...
 $ yearsSincePlanting: num  1.34 9.36 1.34 9.36 1.34 ...
 $ daysSincePlanting :Class 'difftime'  atomic [1:12320] 488 3417 488 3417 488 ...
  .. ..- attr(*, "units")= chr "days"
```

2.1 Initial checks

There are the correct number of plots in each species composition treatment: 2 plots for each monoculture and 4-species mixture, 32 plots for the 16-species mixture, and 16 for the enhanced climber cutting plots (sixteen+CC), making 112 plots in total.

```
apply(with(msSurv, table(sp.comp, pl)), 1, function(x) sum(x > 0))

         4.1         4.10         4.11         4.12         4.13
           2            2            2            2            2
        4.14         4.15         4.16          4.2          4.3
           2            2            2            2            2
         4.4          4.5          4.6          4.7          4.8
           2            2            2            2            2
         4.9 argentifolia   beccariana    conformis   faguetiana
           2            2            2            2            2
  ferruginea      gibbosa   johorensis   lanceolata    leprosula
           2            2            2            2            2
 macrophylla   macroptera   malaanonan       ovalis   parvifolia
           2            2            2            2            2
      sangal      sixteen   tomentella
           2           32            2
```

The species compositions of the monocultures and 16-species mixture are clear. The species found in each 4-species mixture are shown below.

```
sapply(unique(as.character(subset(msSurv, sd == 4)$sp.comp)), function(x) unique(msSurv$sp[msSur
    x]))
```

```
     4.13          4.6          4.3           4.10         4.11
[1,] "lanceolata"  "macroptera" "ferruginea"  "johorensis" "gibbosa"
[2,] "macrophylla" "ferruginea" "macrophylla" "lanceolata" "conformis"
[3,] "conformis"   "gibbosa"    "sangal"      "tomentella" "lanceolata"
[4,] "sangal"      "sangal"     "parvifolia"  "conformis"  "tomentella"
     4.5          4.2          4.7           4.12         4.9
[1,] "johorensis" "malaanonan" "sangal"      "malaanonan" "malaanonan"
[2,] "ferruginea" "ovalis"     "macrophylla" "lanceolata" "conformis"
[3,] "beccariana" "macroptera" "ferruginea"  "conformis"  "faguetiana"
[4,] "sangal"     "tomentella" "faguetiana"  "johorensis" "lanceolata"
     4.14         4.16          4.8            4.4            4.1
[1,] "lanceolata" "conformis"   "johorensis"   "argentifolia" "beccariana"
[2,] "conformis"  "lanceolata"  "argentifolia" "parvifolia"   "tomentella"
[3,] "ferruginea" "ferruginea"  "sangal"       "ferruginea"   "leprosula"
[4,] "ovalis"     "macrophylla" "ferruginea"   "sangal"       "malaanonan"
     4.15
[1,] "lanceolata"
[2,] "ovalis"
[3,] "conformis"
[4,] "sangal"
```

There are no incorrect species names in the monoculture plots.

```
with(subset(msSurv, sd == 1),
     all(as.character(sp.comp) == as.character(sp)))
```

```
[1] TRUE
```

Survival data is complete – there are no NAs.

```
anyNA(msSurv$survival)
```

```
[1] FALSE
```

Sample sizes vary among species, but there are many observations for all species in both surveys.

```
table(msSurv$sp, msSurv$survey.type)
```

```

               census1 census2
  argentifolia    2349    2349
  beccariana      3236    3236
  conformis       4337    4230
  faguetiana      2204    2204
```

```
  ferruginea      4183    4086
  gibbosa         3345    3345
  johorensis      2995    2890
  lanceolata      4629    4525
  leprosula       2769    2769
  macrophylla     3226    3136
  macroptera      2769    2769
  malaanonan      3123    3030
  ovalis          3032    3032
  parvifolia      2923    2831
  sangal          4928    4836
  tomentella      3803    3803
```

Missing levels have not been dropped in growth dataset.

```
table(table(msGrowth$tree.id))


    0     2
47681  6160

nrow(msGrowth)

[1] 12320

nlevels(msGrowth$tree.id)

[1] 53841
```

3 Aggregate the data

3.1 Aggregate survival data

By aggregating the individual mortality data to the treatment plot level, we can fit a simpler linear mixed effects model and remove spatial correlation issues that occur at sub-plot scales (see subsubsection 1.3.2). We can then focus on variation among plots, treatments, within and among species.

```
msSurv$survey.date <- as.Date(msSurv$survey.date)

survival <- summaryBy(
  survival + survey.date + X + Y ~ sp + pl + sp.comp + sd + survey.type,
  data = msSurv, keep.names = TRUE, FUN = mean)

survival$survey.date <- as.Date(survival$survey.date, origin = "1970-01-01")
survival$spBySurveyByComp <- interaction(survival$sp, survival$survey.type,
                                         survival$sp.comp, drop = TRUE)
survival$sp.pl <- interaction(survival$sp, survival$pl, drop = TRUE)
```

```r
dim(survival)
```

```
[1] 1336   11
```

```r
head(survival)
```

```
            sp pl sp.comp sd survey.type survival survey.date   X    Y
1 argentifolia  5 sixteen 16     census1    0.062  2004-02-19  98 -715
2 argentifolia  5 sixteen 16     census2    0.000  2012-02-17  98 -715
3 argentifolia  8 sixteen 16     census1    0.056  2004-02-17 299 -541
4 argentifolia  8 sixteen 16     census2    0.000  2012-03-27 299 -541
5 argentifolia 11 sixteen 16     census1    0.140  2004-02-28 509 -267
6 argentifolia 11 sixteen 16     census2    0.053  2012-07-28 509 -267
              spBySurveyByComp           sp.pl
1 argentifolia.census1.sixteen  argentifolia.5
2 argentifolia.census2.sixteen  argentifolia.5
3 argentifolia.census1.sixteen  argentifolia.8
4 argentifolia.census2.sixteen  argentifolia.8
5 argentifolia.census1.sixteen argentifolia.11
6 argentifolia.census2.sixteen argentifolia.11
```

Number of tree observations that aggregated data were based on.

```r
numObs <- summaryBy(
  survival + survey.date + X + Y ~ sp + pl + sp.comp + sd + survey.type,
  data = msSurv, keep.names = TRUE, FUN = length)
summary(numObs$survival)
```

```
   Min. 1st Qu.  Median    Mean 3rd Qu.    Max. 
     16      29      37      80      63    1040 
```

3.2 Aggregate growth data

Now aggregate the growth data to the plot level in the same the survival were aggregated.

```r
growth <- aggregate(
  x = list(mean.log.diam = log10(msGrowth$d.base),
           mean.daysSincePlanting = msGrowth$daysSincePlanting,
           mean.X = msGrowth$X,
           mean.Y = msGrowth$Y),
  by = list(pl = msGrowth$pl,
            sd = msGrowth$sd,
            sp = msGrowth$sp,
            sp.comp = msGrowth$sp.comp,
            survey.type = msGrowth$survey.type),
  FUN = mean)

## lmer suggests rescaling...see also help("convergence")
growth$mdsp.sc <- scale(growth$mean.daysSincePlanting)
```

```r
growth$mdsp.sc <- as.numeric(growth$mdsp.sc)
growth$mean.daysSincePlanting <- as.numeric(growth$mean.daysSincePlanting)

## Add a new variable which is the interaction between pl and sp
growth$sp.pl <- interaction(growth$sp, growth$pl, drop = TRUE)
## Add a new variable which is the interaction between sp and sp.comp
growth$SP.sp.comp <- interaction(growth$sp, growth$sp.comp, drop = TRUE)

dim(growth)

[1] 1122   12

head(growth)

   pl sd          sp sp.comp survey.type mean.log.diam
1  58  4 beccariana     4.1     census1          0.74
2 109  4 beccariana     4.1     census1          0.89
3  58  4  leprosula     4.1     census1          0.82
4 109  4  leprosula     4.1     census1          0.92
5  58  4 malaanonan     4.1     census1          0.82
6 109  4 malaanonan     4.1     census1          0.93
  mean.daysSincePlanting mean.X mean.Y mdsp.sc          sp.pl
1                    808   2312   -440   -0.95  beccariana.58
2                    592    412   1024   -1.10 beccariana.109
3                    808   2334   -473   -0.95   leprosula.58
4                    592    397   1032   -1.10  leprosula.109
5                    808   2289   -468   -0.95  malaanonan.58
6                    592    372   1009   -1.10 malaanonan.109
      SP.sp.comp
1 beccariana.4.1
2 beccariana.4.1
3  leprosula.4.1
4  leprosula.4.1
5 malaanonan.4.1
6 malaanonan.4.1
```

4 Exploratory plotting

The spatial coordinate data maps the experiment quite well. The square gaps are the unplanted control plots.

For survival data:

```
plot(Y ~ X, data = msSurv, asp = 1, pch = 20)
```

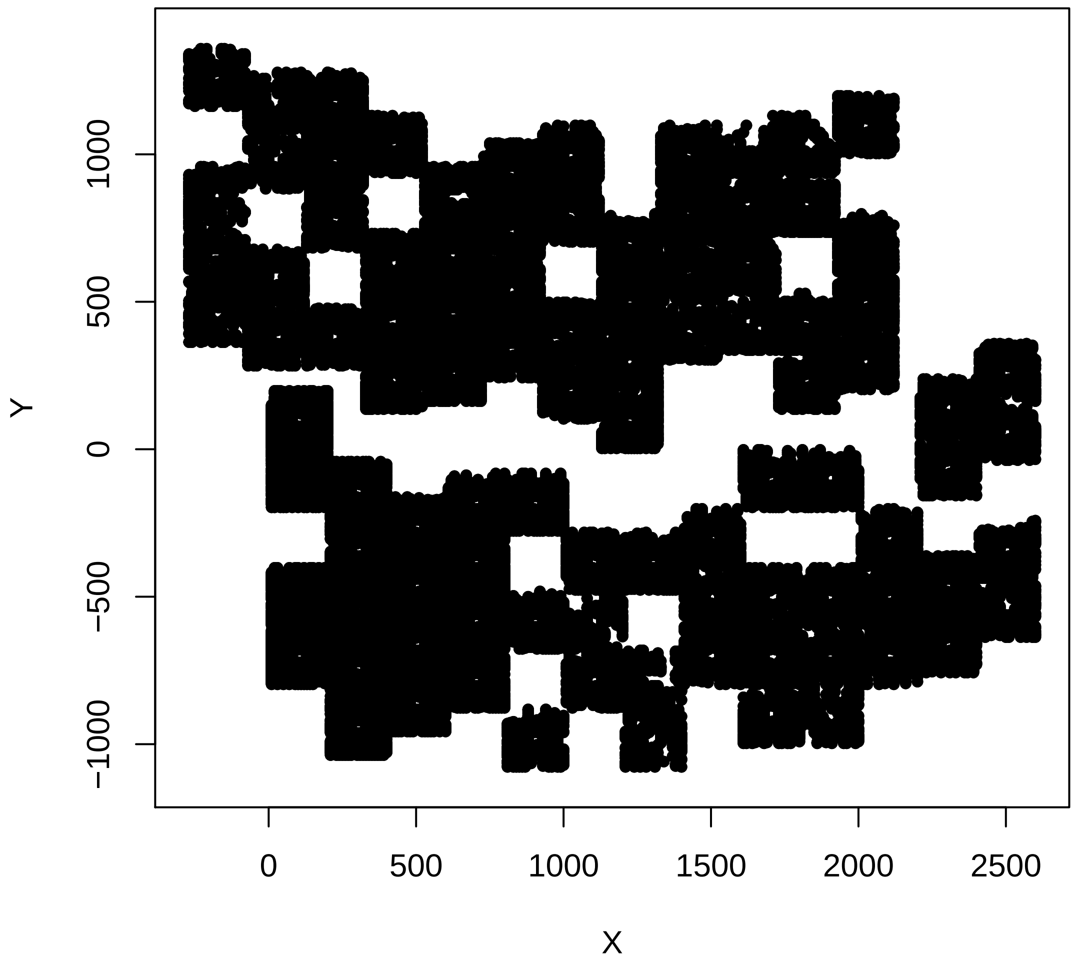

And for growth data:

```
plot(Y ~ X, data = msGrowth, asp = 1, pch = 20)
```

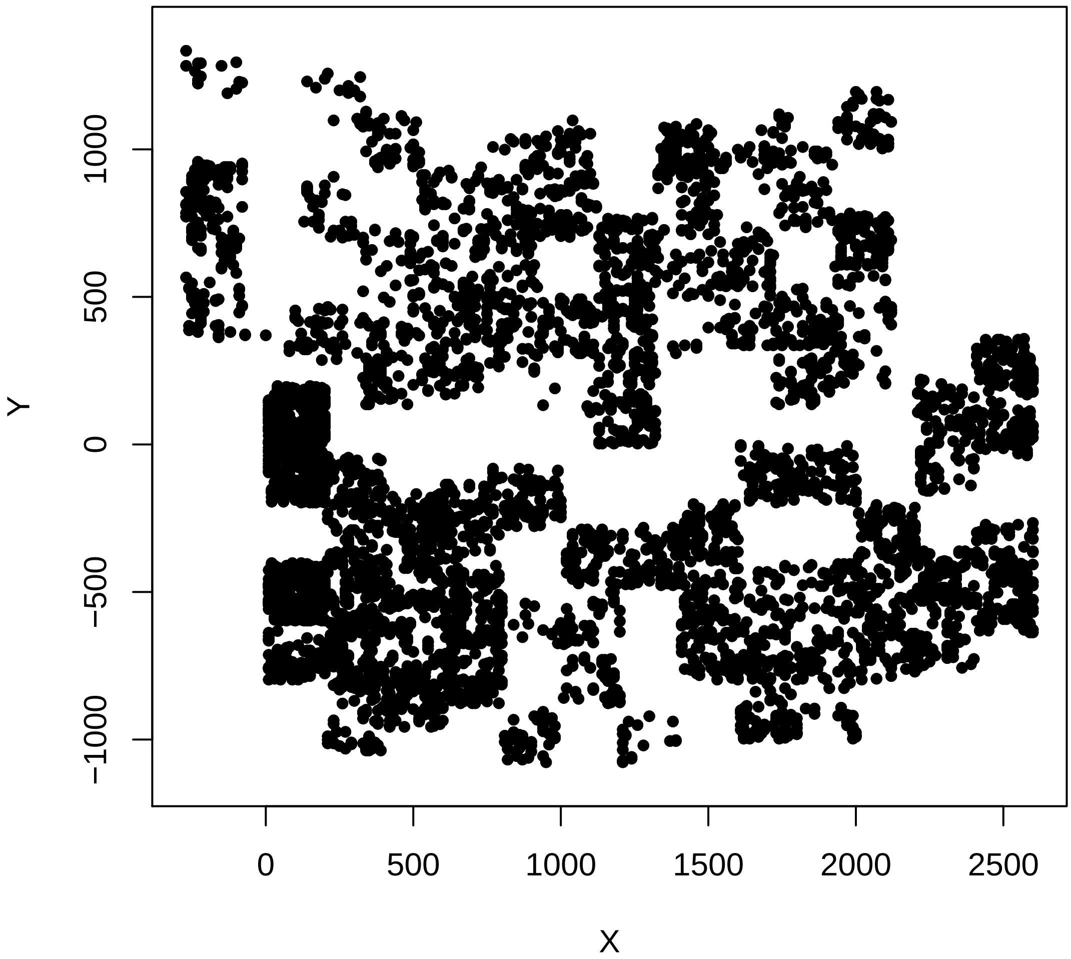

4.1 Explore survival data

4.1.1 Seedling ages

Histograms of days since planting for each seedling in the first and second surveys show that age varies within a survey. But can safely ignore this (see subsubsection 4.2.1).

```
hist(msSurv$yearsSincePlanting[msSurv$survey.type == "census1"])
```

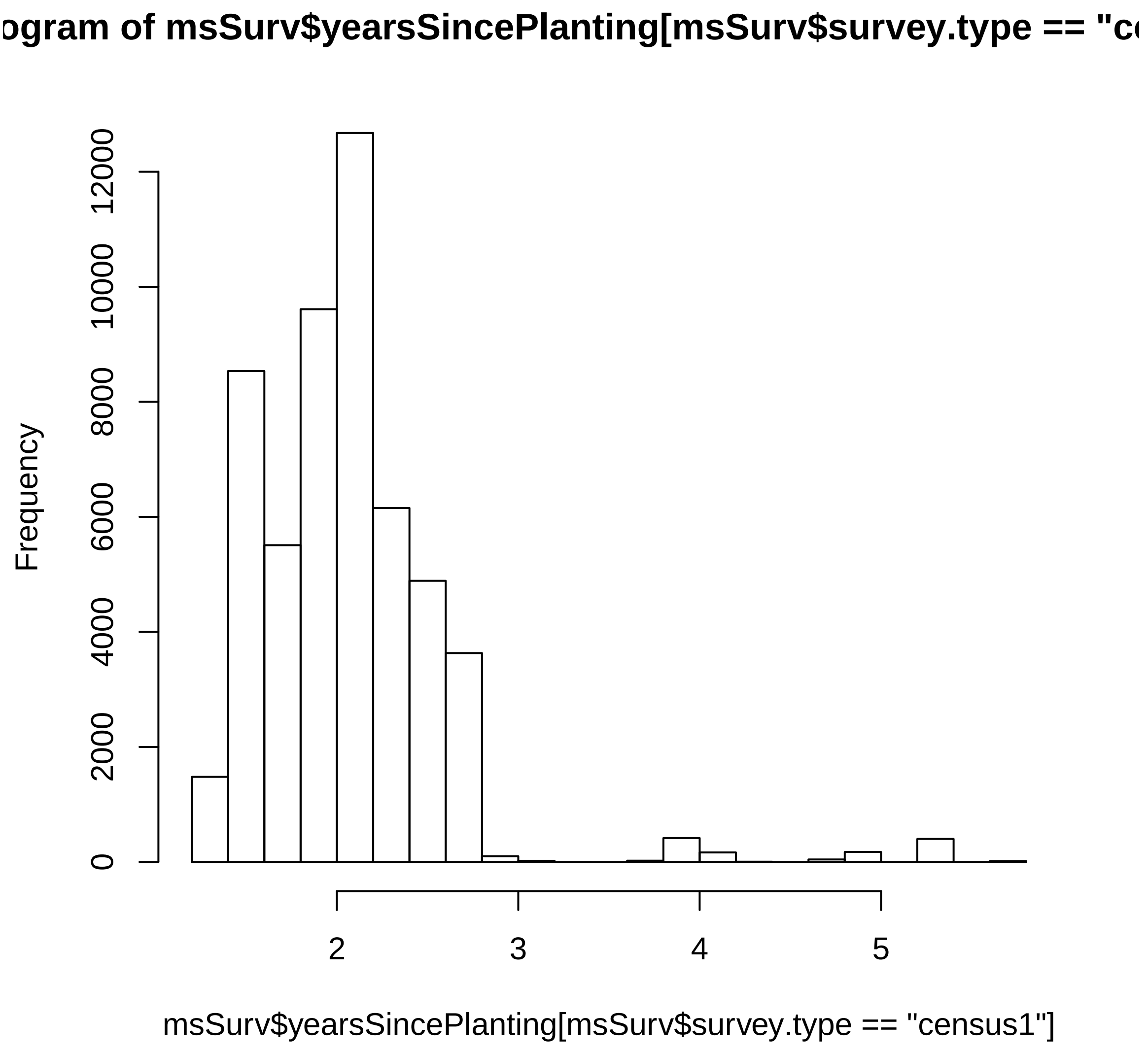


```
summary(msSurv$yearsSincePlanting[msSurv$survey.type == "census1"])

   Min. 1st Qu.  Median    Mean 3rd Qu.    Max. 
    1.3     1.6     2.0     2.1     2.2     5.8 

hist(msSurv$yearsSincePlanting[msSurv$survey.type == "census2"])
```

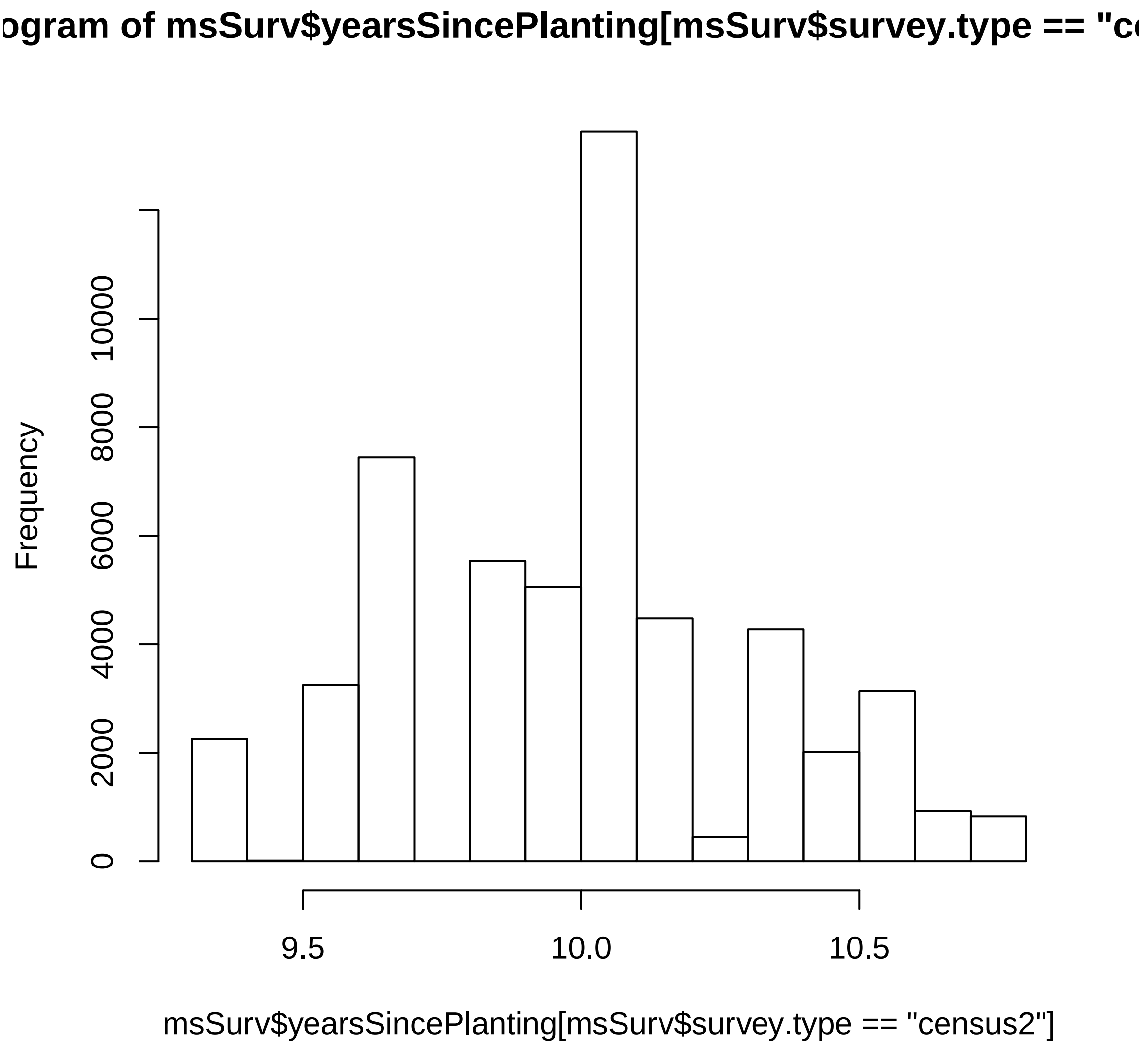


```
summary(msSurv$yearsSincePlanting[msSurv$survey.type == "census2"])

   Min. 1st Qu.  Median    Mean 3rd Qu.    Max.
    9.3     9.8    10.0    10.0    10.1    10.7
```

4.1.2 Survival response to richness

There seems no clear overall relationship between survival and species diversity. There are mixed patterns among species, however; some species are not responsive to the richness treatment, whereas others show a nonlinear pattern across the treatment levels.

```
qplot(y = survival, x = sd, group = sp, colour = sp, data = msSurv,
      geom = "smooth", method = "loess") + theme_bw()
```

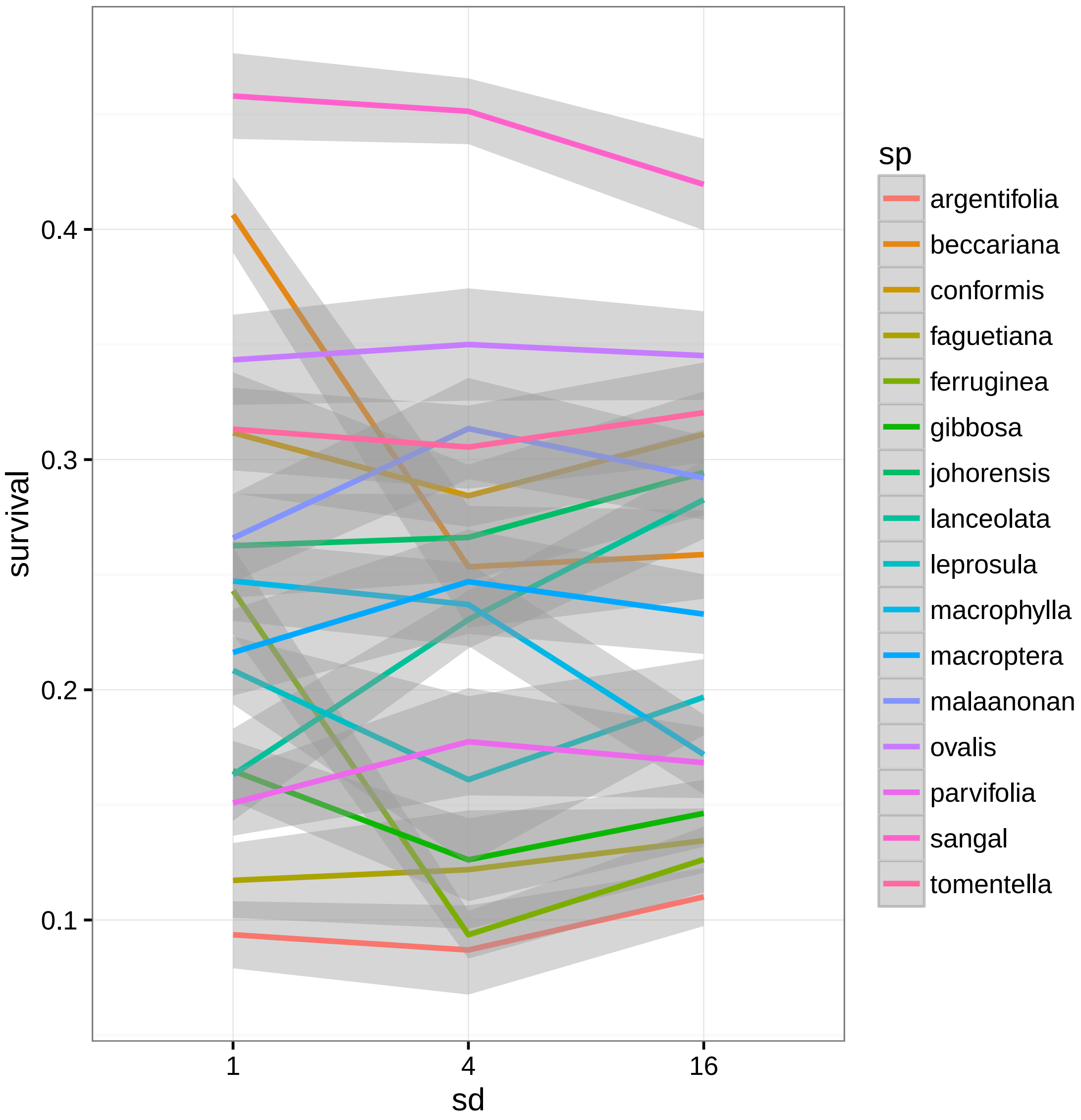

4.1.3 Survival response to composition

The aggregated data suggest that there are no consistent effects of species composition treatments. Need to model this formally.

```
qplot(y = sp, x = survival, group = sp.comp, colour = sd,
      data = subset(survival, survey.type == "census1"),
      geom = "point")
```

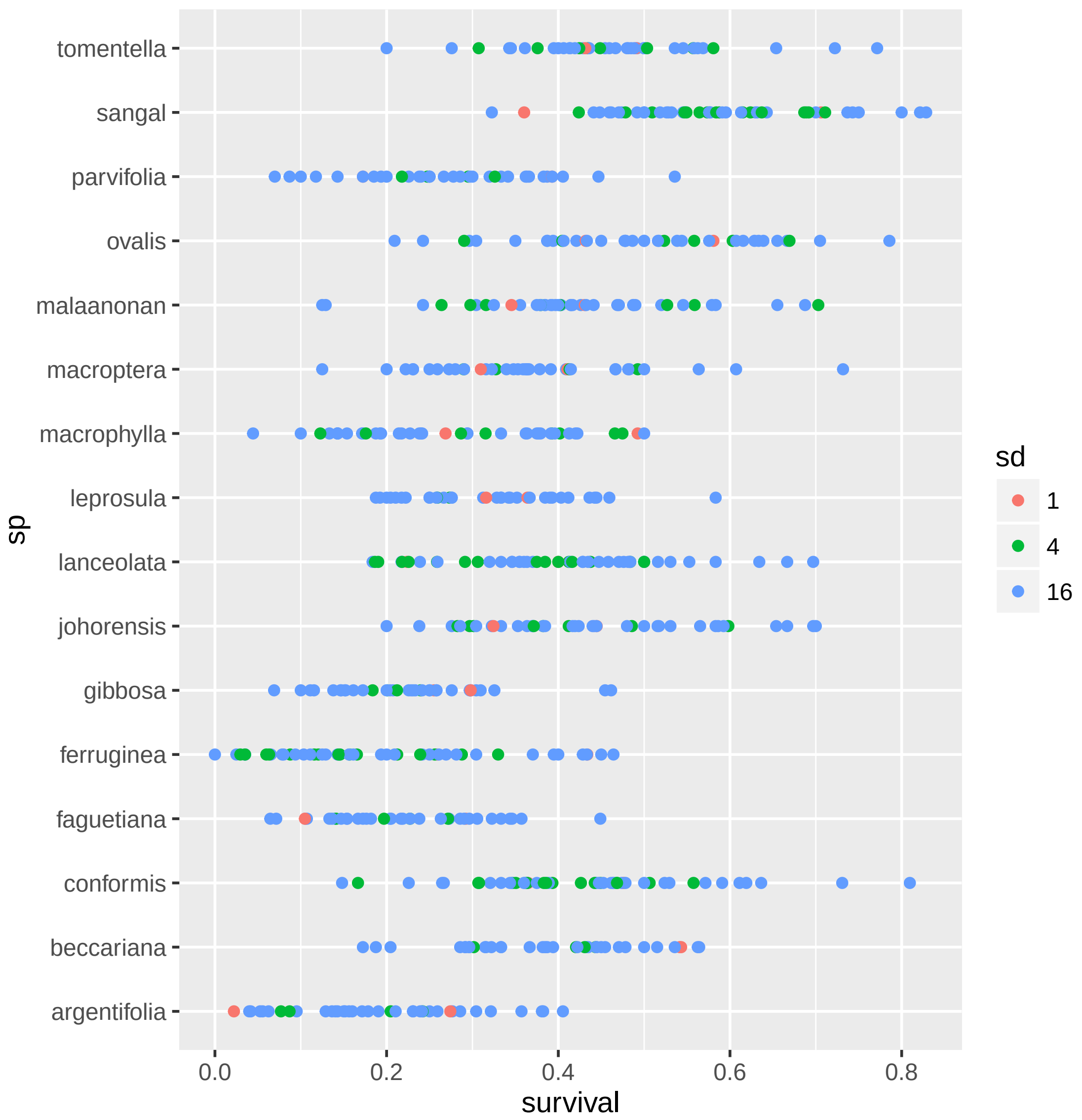


```
qplot(y = sp, x = survival, group = sp.comp, colour = sd,
      data = subset(survival, survey.type == "census2"),
      geom = "point")
```

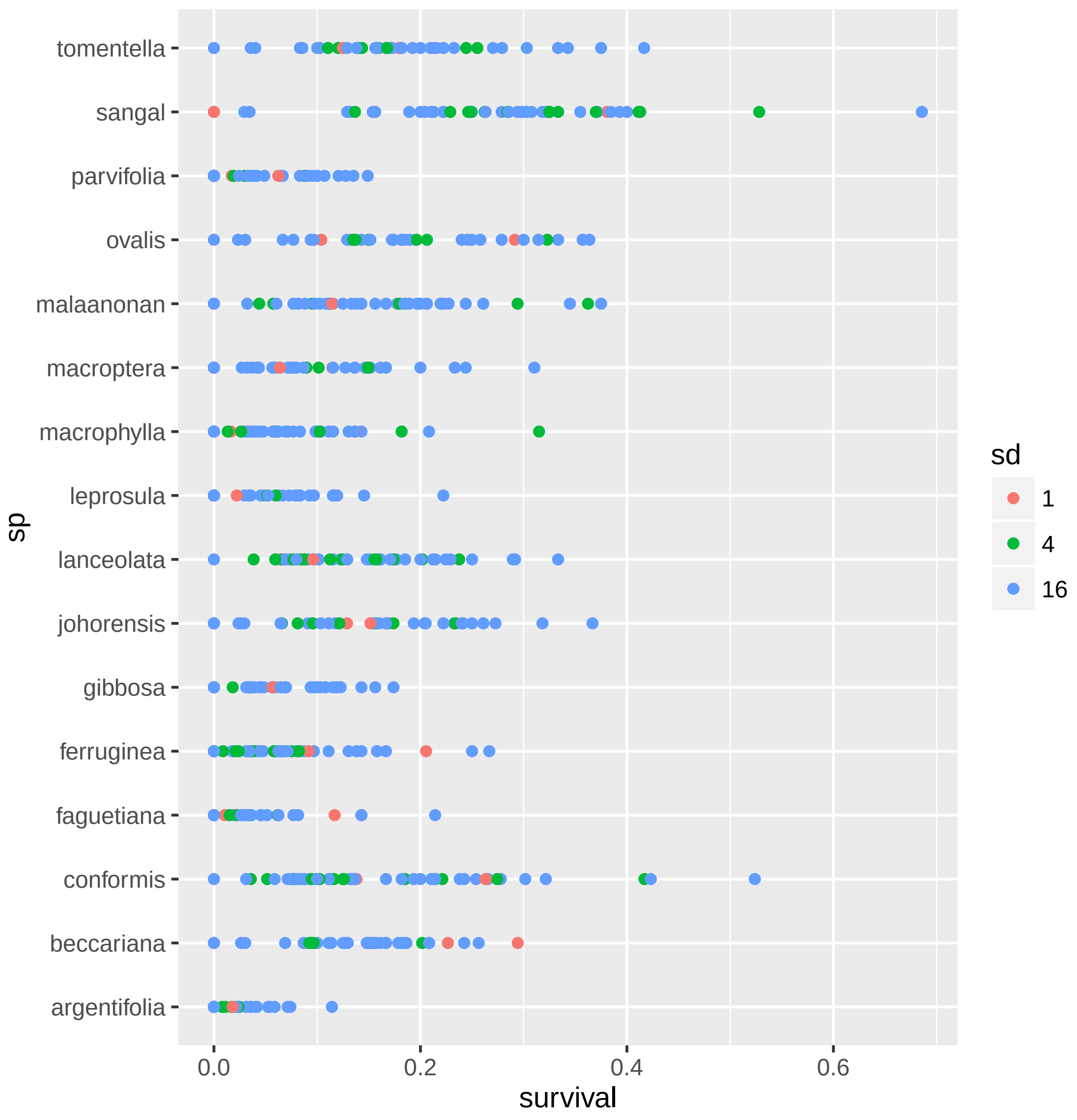
tomentella
sangal
parvifolia
ovalis
malaanonan
macroptera
macrophylla
leprosula
lanceolata
johorensis
gibbosa
ferruginea
faguetiana
conformis
beccariana
argentifolia
sp
0.0
0.2
0.4
0.6
survival
sd
1
4
16

4.2 Explore growth data

4.2.1 Survey times

If there are huge differences (not the case) between plot times of survey, this would add noise to our analysis. But surveys are clearly separate and there is no problem, especially as treatments have been allocated randomly to plots. In any case, time is still a predictor in the model (as explained in subsubsection 1.3.2).

```
plot(daysSincePlanting ~ pl, data = msGrowth)
```

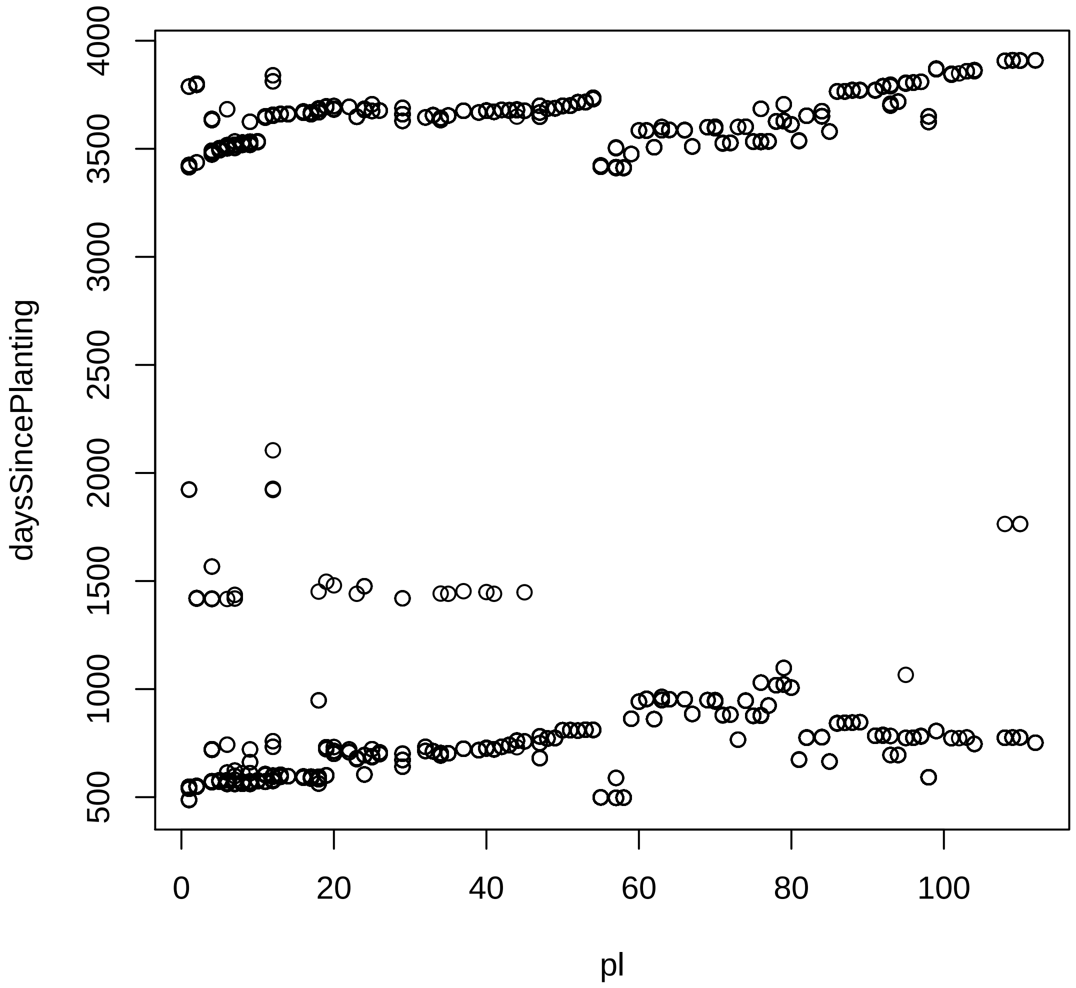

4.2.2 Growth response to composition

Now show how growth (diameter time) varies between species, and between species composition treatments for each species. Each point on this graph is sp:plot:survey.type combination. Regression lines represent different sp.comp treatments.

```
xyplot(mean.log.diam ~ mean.daysSincePlanting | sp, data = growth,
       type = c("p", "r", "g"),
       groups = sp.comp,
       scales = list(x = list(rot = 45)),
       cex = 0.3)
```

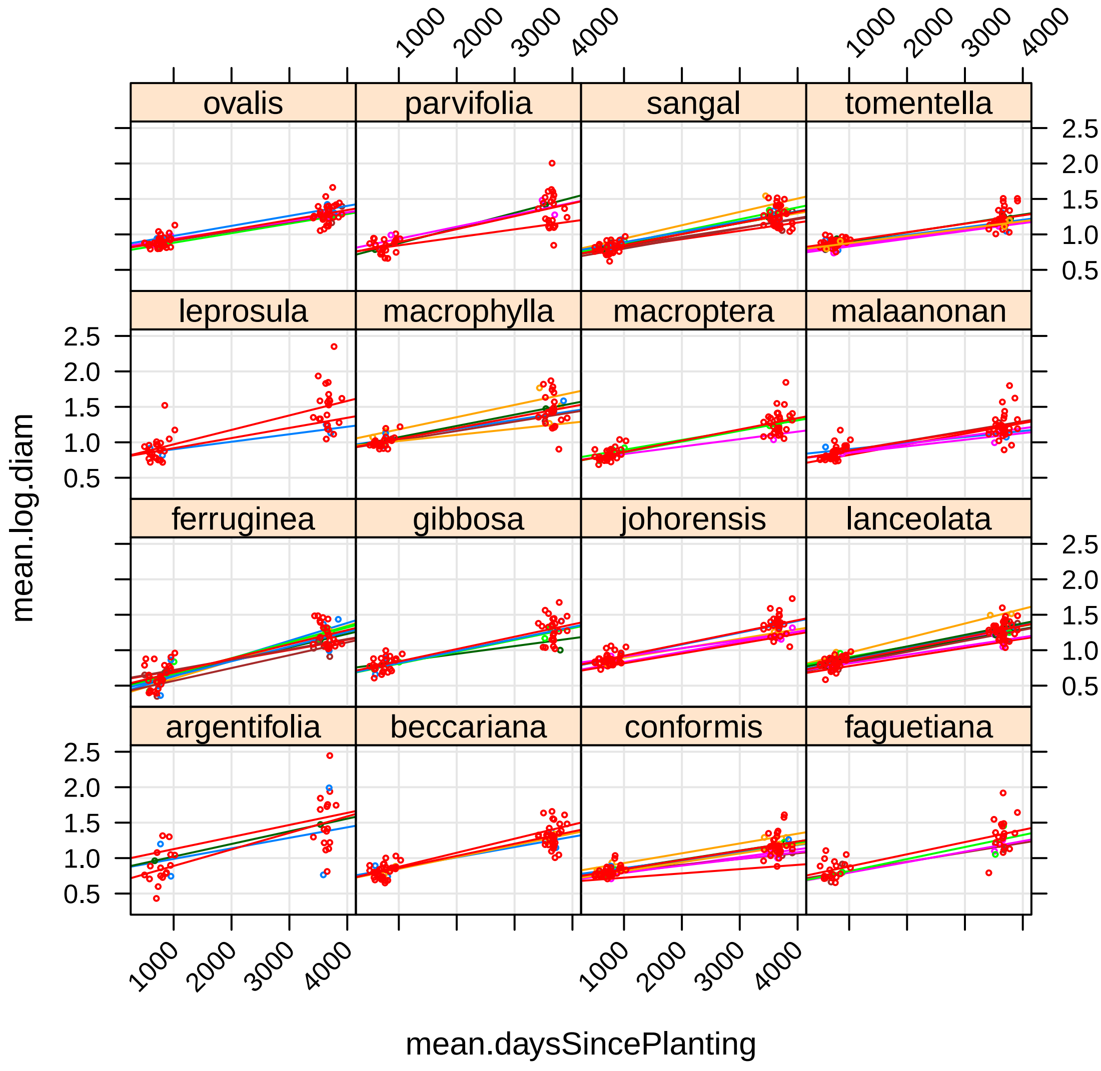

The next graph shows more clearly that sp:sp.comp random intercepts and slopes are strongly correlated. Within each panel the regression lines are diverging with surprisingly few crossings.

```
xyplot(mean.log.diam ~ mean.daysSincePlanting | sp, data = growth,
       type = c("p","r","g"),
       groups = sp.comp,
       scales = list(y = list(relation = "free", at = NULL),
                     x = list(rot = 45)),
```

```
    between = list(x = -1),
    cex = 0.3) #, auto.key=list(space="right"))
```

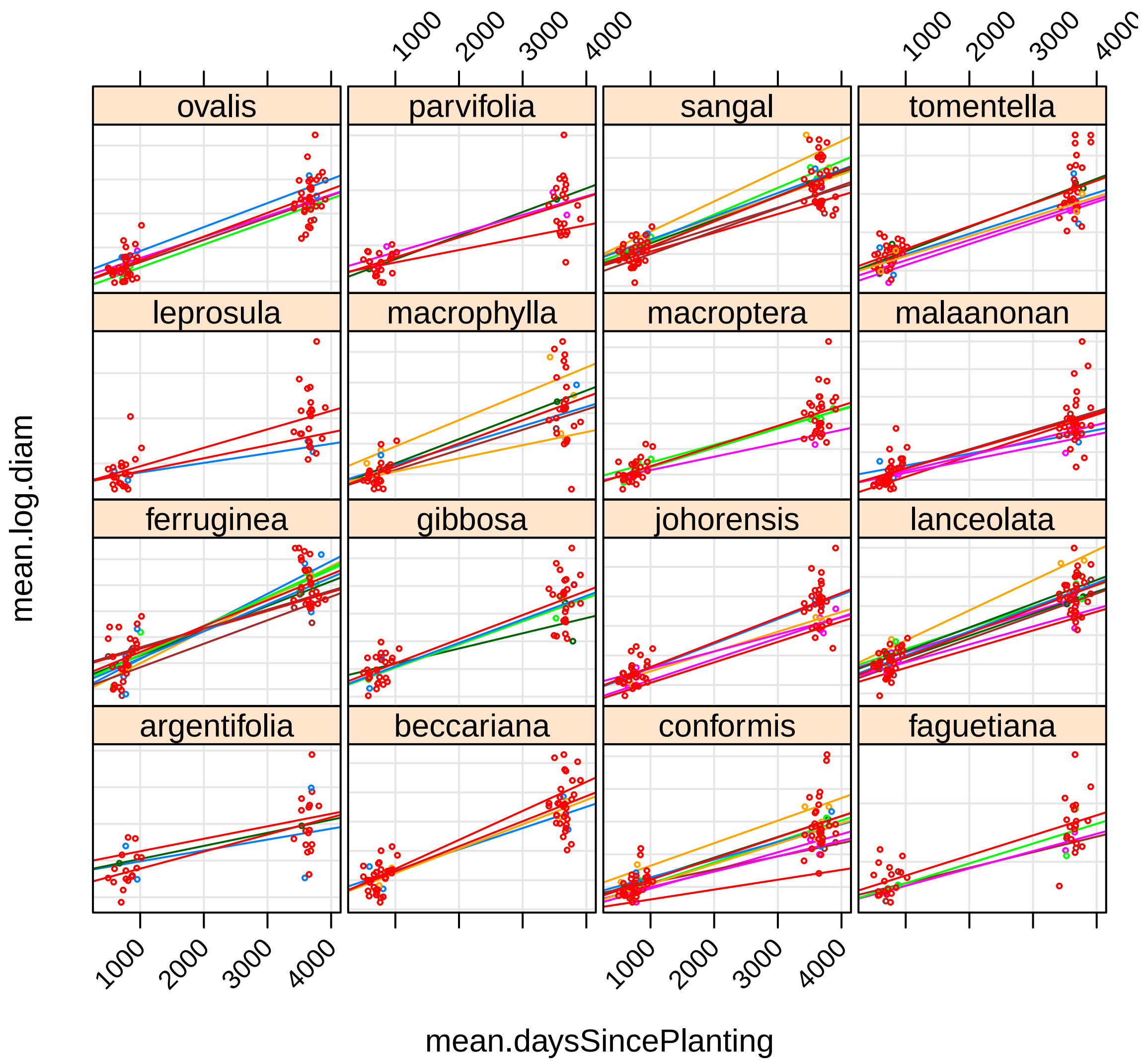

5 Modelling

I have aggregated the data to get the proportion of seedlings that have survived in each plot for both surveys. At each plot, there is also a species composition and corresponding species richness treatment. The average survey date within this plot and survey is also included. I will model survival as a function of species, interacting with survey.type and species composition, plus a plot:sp random intercepts variable. I will fit a linear mixed model to the data, then check diagnostic plots to ensure that normal assumptions are ok. The proportion data, which varies between 0 and 1, is not ideal but we are not conducting inference on these data, we are only getting estimates. These estimates will be unbiased anyway, so we shouldn't worry too much. I can check the spatial autocorrelation in survival to see whether aggregating the data has solved this issue.

5.1 Fit the model for survival

```
survivalModel <- lmer(survival ~ spBySurveyByComp + (1 | sp.pl), data = survival)
summary(survivalModel)

Linear mixed model fit by REML ['lmerMod']
Formula: survival ~ spBySurveyByComp + (1 | sp.pl)
   Data: survival

REML criterion at convergence: -2009

Scaled residuals:
    Min      1Q  Median      3Q     Max
-2.3750 -0.4519 -0.0005  0.4462  2.7971

Random effects:
 Groups   Name        Variance Std.Dev.
 sp.pl    (Intercept) 0.00564  0.0751
 Residual             0.00445  0.0667
Number of obs: 1336, groups:  sp.pl, 672

Fixed effects:
                                                  Estimate Std. Error
(Intercept)                                        0.43716    0.07101
spBySurveyByCompleprosula.census1.4.1             -0.17110    0.10043
spBySurveyByCompmalaanonan.census1.4.1             0.07219    0.10043
spBySurveyByComptomentella.census1.4.1             0.03886    0.10043
spBySurveyByCompbeccariana.census2.4.1            -0.29020    0.06669
spBySurveyByCompleprosula.census2.4.1             -0.38138    0.10043
spBySurveyByCompmalaanonan.census2.4.1            -0.23407    0.10043
spBySurveyByComptomentella.census2.4.1            -0.23013    0.10043
spBySurveyByCompconformis.census1.4.10            -0.07054    0.10043
spBySurveyByCompjohorensis.census1.4.10           -0.08977    0.10043
spBySurveyByComplanceolata.census1.4.10           -0.09205    0.10043
spBySurveyByComptomentella.census1.4.10            0.00681    0.10043
spBySurveyByCompconformis.census2.4.10            -0.28643    0.10043
spBySurveyByCompjohorensis.census2.4.10           -0.30966    0.10043
spBySurveyByComplanceolata.census2.4.10           -0.30672    0.10043
spBySurveyByComptomentella.census2.4.10           -0.28298    0.10043
spBySurveyByCompconformis.census1.4.11            -0.08201    0.10043
spBySurveyByCompgibbosa.census1.4.11              -0.23065    0.10043
spBySurveyByComplanceolata.census1.4.11           -0.17120    0.10043
spBySurveyByComptomentella.census1.4.11           -0.00765    0.10043
spBySurveyByCompconformis.census2.4.11            -0.33491    0.10043
spBySurveyByCompgibbosa.census2.4.11              -0.41021    0.10043
spBySurveyByComplanceolata.census2.4.11           -0.34772    0.10043
spBySurveyByComptomentella.census2.4.11           -0.31020    0.10043
spBySurveyByCompconformis.census1.4.12            -0.16224    0.10043
spBySurveyByCompjohorensis.census1.4.12           -0.10309    0.10043
spBySurveyByComplanceolata.census1.4.12           -0.14068    0.10043
spBySurveyByCompmalaanonan.census1.4.12           -0.02491    0.10043
spBySurveyByCompconformis.census2.4.12            -0.34093    0.11641
spBySurveyByCompjohorensis.census2.4.12           -0.35034    0.11641
spBySurveyByComplanceolata.census2.4.12           -0.35480    0.11641
```

```
spBySurveyByCompmalaanonan.census2.4.12            -0.27784    0.11641
spBySurveyByCompconformis.census1.4.13              0.05024    0.10043
spBySurveyByComplanceolata.census1.4.13            -0.09970    0.10043
spBySurveyByCompmacrophylla.census1.4.13           -0.09297    0.10043
spBySurveyByCompsangal.census1.4.13                 0.10933    0.10043
spBySurveyByCompconformis.census2.4.13             -0.18128    0.10043
spBySurveyByComplanceolata.census2.4.13            -0.27417    0.10043
spBySurveyByCompmacrophylla.census2.4.13           -0.27305    0.10043
spBySurveyByCompsangal.census2.4.13                -0.10477    0.10043
spBySurveyByCompconformis.census1.4.14              0.03818    0.10043
spBySurveyByCompferruginea.census1.4.14            -0.23489    0.10043
spBySurveyByComplanceolata.census1.4.14            -0.03871    0.10043
spBySurveyByCompovalis.census1.4.14                 0.09987    0.10043
spBySurveyByCompconformis.census2.4.14             -0.26153    0.10043
spBySurveyByCompferruginea.census2.4.14            -0.37058    0.10043
spBySurveyByComplanceolata.census2.4.14            -0.35484    0.10043
spBySurveyByCompovalis.census2.4.14                -0.20828    0.10043
spBySurveyByCompconformis.census1.4.15             -0.06207    0.10043
spBySurveyByComplanceolata.census1.4.15            -0.19827    0.10043
spBySurveyByCompovalis.census1.4.15                -0.03032    0.10043
spBySurveyByCompsangal.census1.4.15                 0.18214    0.10043
spBySurveyByCompconformis.census2.4.15             -0.26404    0.10043
spBySurveyByComplanceolata.census2.4.15            -0.32876    0.10043
spBySurveyByCompovalis.census2.4.15                -0.27048    0.10043
spBySurveyByCompsangal.census2.4.15                -0.06916    0.10043
spBySurveyByCompconformis.census1.4.16             -0.01020    0.10043
spBySurveyByCompferruginea.census1.4.16            -0.28673    0.10043
spBySurveyByComplanceolata.census1.4.16             0.00478    0.10043
spBySurveyByCompmacrophylla.census1.4.16           -0.13838    0.10043
spBySurveyByCompconformis.census2.4.16             -0.32452    0.10043
spBySurveyByCompferruginea.census2.4.16            -0.39575    0.10043
spBySurveyByComplanceolata.census2.4.16            -0.31485    0.10043
spBySurveyByCompmacrophylla.census2.4.16           -0.33309    0.10043
spBySurveyByCompmacroptera.census1.4.2             -0.04596    0.10043
spBySurveyByCompmalaanonan.census1.4.2             -0.10430    0.10043
spBySurveyByCompovalis.census1.4.2                  0.14360    0.10043
spBySurveyByComptomentella.census1.4.2              0.02944    0.10043
spBySurveyByCompmacroptera.census2.4.2             -0.31181    0.10043
spBySurveyByCompmalaanonan.census2.4.2             -0.29142    0.10043
spBySurveyByCompovalis.census2.4.2                 -0.25898    0.10043
spBySurveyByComptomentella.census2.4.2             -0.25487    0.10043
spBySurveyByCompferruginea.census1.4.3             -0.24226    0.10043
spBySurveyByCompmacrophylla.census1.4.3            -0.19159    0.10043
spBySurveyByCompparvifolia.census1.4.3             -0.12654    0.10043
spBySurveyByCompsangal.census1.4.3                  0.02948    0.10043
spBySurveyByCompferruginea.census2.4.3             -0.32944    0.11641
spBySurveyByCompmacrophylla.census2.4.3            -0.37705    0.11641
spBySurveyByCompparvifolia.census2.4.3             -0.34041    0.11641
spBySurveyByCompsangal.census2.4.3                 -0.17640    0.11641
spBySurveyByCompargentifolia.census1.4.4           -0.29634    0.10043
spBySurveyByCompferruginea.census1.4.4             -0.34680    0.10043
spBySurveyByCompparvifolia.census1.4.4             -0.20444    0.10043
```

```
spBySurveyByCompsangal.census1.4.4                  0.16677    0.10043
spBySurveyByCompargentifolia.census2.4.4           -0.41680    0.10043
spBySurveyByCompferruginea.census2.4.4             -0.39879    0.10043
spBySurveyByCompparvifolia.census2.4.4             -0.41278    0.10043
spBySurveyByCompsangal.census2.4.4                 -0.10674    0.10043
spBySurveyByCompbeccariana.census1.4.5             -0.07602    0.10043
spBySurveyByCompferruginea.census1.4.5             -0.39077    0.10043
spBySurveyByCompjohorensis.census1.4.5             -0.00313    0.10043
spBySurveyByCompsangal.census1.4.5                  0.18077    0.10043
spBySurveyByCompbeccariana.census2.4.5             -0.34126    0.10043
spBySurveyByCompferruginea.census2.4.5             -0.41874    0.10043
spBySurveyByCompjohorensis.census2.4.5             -0.29801    0.10043
spBySurveyByCompsangal.census2.4.5                 -0.18961    0.10043
spBySurveyByCompferruginea.census1.4.6             -0.28713    0.10043
spBySurveyByCompgibbosa.census1.4.6                -0.22551    0.10043
spBySurveyByCompmacroptera.census1.4.6             -0.06689    0.10043
spBySurveyByCompsangal.census1.4.6                  0.16822    0.10043
spBySurveyByCompferruginea.census2.4.6             -0.37633    0.10043
spBySurveyByCompgibbosa.census2.4.6                -0.38559    0.10043
spBySurveyByCompmacroptera.census2.4.6             -0.31747    0.10043
spBySurveyByCompsangal.census2.4.6                 -0.12108    0.10043
spBySurveyByCompfaguetiana.census1.4.7             -0.20287    0.10043
spBySurveyByCompferruginea.census1.4.7             -0.23308    0.10043
spBySurveyByCompmacrophylla.census1.4.7            -0.00813    0.10043
spBySurveyByCompsangal.census1.4.7                  0.21201    0.10043
spBySurveyByCompfaguetiana.census2.4.7             -0.39872    0.10043
spBySurveyByCompferruginea.census2.4.7             -0.36594    0.10043
spBySurveyByCompmacrophylla.census2.4.7            -0.35661    0.10043
spBySurveyByCompsangal.census2.4.7                 -0.16020    0.10043
spBySurveyByCompargentifolia.census1.4.8           -0.27271    0.10043
spBySurveyByCompferruginea.census1.4.8             -0.25116    0.10043
spBySurveyByCompjohorensis.census1.4.8              0.01259    0.10043
spBySurveyByCompsangal.census1.4.8                  0.18954    0.10043
spBySurveyByCompargentifolia.census2.4.8           -0.42769    0.10043
spBySurveyByCompferruginea.census2.4.8             -0.39357    0.10043
spBySurveyByCompjohorensis.census2.4.8             -0.27239    0.10043
spBySurveyByCompsangal.census2.4.8                 -0.10920    0.10043
spBySurveyByCompconformis.census1.4.9              -0.03741    0.10043
spBySurveyByCompfaguetiana.census1.4.9             -0.25293    0.10043
spBySurveyByComplanceolata.census1.4.9              0.03159    0.10043
spBySurveyByCompmalaanonan.census1.4.9              0.03191    0.10043
spBySurveyByCompconformis.census2.4.9              -0.30424    0.10043
spBySurveyByCompfaguetiana.census2.4.9             -0.40815    0.10043
spBySurveyByComplanceolata.census2.4.9             -0.24063    0.10043
spBySurveyByCompmalaanonan.census2.4.9             -0.26136    0.10043
spBySurveyByCompargentifolia.census1.argentifolia -0.28899    0.10043
spBySurveyByCompargentifolia.census2.argentifolia -0.42809    0.10043
spBySurveyByCompbeccariana.census1.beccariana       0.10515    0.10043
spBySurveyByCompbeccariana.census2.beccariana      -0.17672    0.10043
spBySurveyByCompconformis.census1.conformis        -0.01673    0.10043
spBySurveyByCompconformis.census2.conformis        -0.23655    0.10043
spBySurveyByCompfaguetiana.census1.faguetiana      -0.23859    0.10043
```

```
spBySurveyByCompfaguetiana.census2.faguetiana     -0.37346    0.10043
spBySurveyByCompferruginea.census1.ferruginea     -0.09077    0.10043
spBySurveyByCompferruginea.census2.ferruginea     -0.28856    0.10043
spBySurveyByCompgibbosa.census1.gibbosa           -0.16052    0.10043
spBySurveyByCompgibbosa.census2.gibbosa           -0.38030    0.10043
spBySurveyByCompjohorensis.census1.johorensis     -0.05256    0.10043
spBySurveyByCompjohorensis.census2.johorensis     -0.29689    0.10043
spBySurveyByComplanceolata.census1.lanceolata     -0.12531    0.10043
spBySurveyByComplanceolata.census2.lanceolata     -0.35699    0.10043
spBySurveyByCompleprosula.census1.leprosula       -0.09713    0.10043
spBySurveyByCompleprosula.census2.leprosula       -0.36777    0.10043
spBySurveyByCompmacrophylla.census1.macrophylla   -0.05658    0.10043
spBySurveyByCompmacrophylla.census2.macrophylla   -0.36046    0.10043
spBySurveyByCompmacroptera.census1.macroptera     -0.07694    0.10043
spBySurveyByCompmacroptera.census2.macroptera     -0.34784    0.10043
spBySurveyByCompmalaanonan.census1.malaanonan     -0.04897    0.10043
spBySurveyByCompmalaanonan.census2.malaanonan     -0.28739    0.10043
spBySurveyByCompovalis.census1.ovalis              0.06920    0.10043
spBySurveyByCompovalis.census2.ovalis             -0.23949    0.10043
spBySurveyByCompparvifolia.census1.parvifolia     -0.15460    0.10043
spBySurveyByCompparvifolia.census2.parvifolia     -0.39728    0.10043
spBySurveyByCompsangal.census1.sangal              0.09608    0.10043
spBySurveyByCompsangal.census2.sangal             -0.24673    0.10043
spBySurveyByCompargentifolia.census1.sixteen      -0.23621    0.07320
spBySurveyByCompbeccariana.census1.sixteen        -0.05373    0.07320
spBySurveyByCompconformis.census1.sixteen          0.01230    0.07320
spBySurveyByCompfaguetiana.census1.sixteen        -0.21573    0.07320
spBySurveyByCompferruginea.census1.sixteen        -0.22971    0.07320
spBySurveyByCompgibbosa.census1.sixteen           -0.20980    0.07320
spBySurveyByCompjohorensis.census1.sixteen         0.01152    0.07320
spBySurveyByComplanceolata.census1.sixteen        -0.00793    0.07320
spBySurveyByCompleprosula.census1.sixteen         -0.11432    0.07320
spBySurveyByCompmacrophylla.census1.sixteen       -0.17227    0.07320
spBySurveyByCompmacroptera.census1.sixteen        -0.07006    0.07320
spBySurveyByCompmalaanonan.census1.sixteen        -0.01499    0.07320
spBySurveyByCompovalis.census1.sixteen             0.05749    0.07320
spBySurveyByCompparvifolia.census1.sixteen        -0.16257    0.07320
spBySurveyByCompsangal.census1.sixteen             0.14348    0.07320
spBySurveyByComptomentella.census1.sixteen         0.03351    0.07320
spBySurveyByCompargentifolia.census2.sixteen      -0.41530    0.07320
spBySurveyByCompbeccariana.census2.sixteen        -0.30257    0.07320
spBySurveyByCompconformis.census2.sixteen         -0.26580    0.07320
spBySurveyByCompfaguetiana.census2.sixteen        -0.39920    0.07320
spBySurveyByCompferruginea.census2.sixteen        -0.36534    0.07320
spBySurveyByCompgibbosa.census2.sixteen           -0.37298    0.07320
spBySurveyByCompjohorensis.census2.sixteen        -0.28384    0.07320
spBySurveyByComplanceolata.census2.sixteen        -0.28155    0.07320
spBySurveyByCompleprosula.census2.sixteen         -0.37852    0.07320
spBySurveyByCompmacrophylla.census2.sixteen       -0.37787    0.07320
spBySurveyByCompmacroptera.census2.sixteen        -0.34046    0.07320
spBySurveyByCompmalaanonan.census2.sixteen        -0.28121    0.07320
spBySurveyByCompovalis.census2.sixteen            -0.25603    0.07320
```

```
spBySurveyByCompparvifolia.census2.sixteen        -0.38927    0.07320
spBySurveyByCompsangal.census2.sixteen            -0.18085    0.07320
spBySurveyByComptomentella.census2.sixteen        -0.24838    0.07320
spBySurveyByComptomentella.census1.tomentella      0.02386    0.10043
spBySurveyByComptomentella.census2.tomentella     -0.28495    0.10043
                                                  t value
(Intercept)                                          6.16
spBySurveyByCompleprosula.census1.4.1               -1.70
spBySurveyByCompmalaanonan.census1.4.1               0.72
spBySurveyByComptomentella.census1.4.1               0.39
spBySurveyByCompbeccariana.census2.4.1              -4.35
spBySurveyByCompleprosula.census2.4.1               -3.80
spBySurveyByCompmalaanonan.census2.4.1              -2.33
spBySurveyByComptomentella.census2.4.1              -2.29
spBySurveyByCompconformis.census1.4.10              -0.70
spBySurveyByCompjohorensis.census1.4.10             -0.89
spBySurveyByComplanceolata.census1.4.10             -0.92
spBySurveyByComptomentella.census1.4.10              0.07
spBySurveyByCompconformis.census2.4.10              -2.85
spBySurveyByCompjohorensis.census2.4.10             -3.08
spBySurveyByComplanceolata.census2.4.10             -3.05
spBySurveyByComptomentella.census2.4.10             -2.82
spBySurveyByCompconformis.census1.4.11              -0.82
spBySurveyByCompgibbosa.census1.4.11                -2.30
spBySurveyByComplanceolata.census1.4.11             -1.70
spBySurveyByComptomentella.census1.4.11             -0.08
spBySurveyByCompconformis.census2.4.11              -3.33
spBySurveyByCompgibbosa.census2.4.11                -4.08
spBySurveyByComplanceolata.census2.4.11             -3.46
spBySurveyByComptomentella.census2.4.11             -3.09
spBySurveyByCompconformis.census1.4.12              -1.62
spBySurveyByCompjohorensis.census1.4.12             -1.03
spBySurveyByComplanceolata.census1.4.12             -1.40
spBySurveyByCompmalaanonan.census1.4.12             -0.25
spBySurveyByCompconformis.census2.4.12              -2.93
spBySurveyByCompjohorensis.census2.4.12             -3.01
spBySurveyByComplanceolata.census2.4.12             -3.05
spBySurveyByCompmalaanonan.census2.4.12             -2.39
spBySurveyByCompconformis.census1.4.13               0.50
spBySurveyByComplanceolata.census1.4.13             -0.99
spBySurveyByCompmacrophylla.census1.4.13            -0.93
spBySurveyByCompsangal.census1.4.13                  1.09
spBySurveyByCompconformis.census2.4.13              -1.81
spBySurveyByComplanceolata.census2.4.13             -2.73
spBySurveyByCompmacrophylla.census2.4.13            -2.72
spBySurveyByCompsangal.census2.4.13                 -1.04
spBySurveyByCompconformis.census1.4.14               0.38
spBySurveyByCompferruginea.census1.4.14             -2.34
spBySurveyByComplanceolata.census1.4.14             -0.39
spBySurveyByCompovalis.census1.4.14                  0.99
spBySurveyByCompconformis.census2.4.14              -2.60
spBySurveyByCompferruginea.census2.4.14             -3.69
```

```
spBySurveyByComplanceolata.census2.4.14            -3.53
spBySurveyByCompovalis.census2.4.14                -2.07
spBySurveyByCompconformis.census1.4.15             -0.62
spBySurveyByComplanceolata.census1.4.15            -1.97
spBySurveyByCompovalis.census1.4.15                -0.30
spBySurveyByCompsangal.census1.4.15                 1.81
spBySurveyByCompconformis.census2.4.15             -2.63
spBySurveyByComplanceolata.census2.4.15            -3.27
spBySurveyByCompovalis.census2.4.15                -2.69
spBySurveyByCompsangal.census2.4.15                -0.69
spBySurveyByCompconformis.census1.4.16             -0.10
spBySurveyByCompferruginea.census1.4.16            -2.86
spBySurveyByComplanceolata.census1.4.16             0.05
spBySurveyByCompmacrophylla.census1.4.16           -1.38
spBySurveyByCompconformis.census2.4.16             -3.23
spBySurveyByCompferruginea.census2.4.16            -3.94
spBySurveyByComplanceolata.census2.4.16            -3.14
spBySurveyByCompmacrophylla.census2.4.16           -3.32
spBySurveyByCompmacroptera.census1.4.2             -0.46
spBySurveyByCompmalaanonan.census1.4.2             -1.04
spBySurveyByCompovalis.census1.4.2                  1.43
spBySurveyByComptomentella.census1.4.2              0.29
spBySurveyByCompmacroptera.census2.4.2             -3.10
spBySurveyByCompmalaanonan.census2.4.2             -2.90
spBySurveyByCompovalis.census2.4.2                 -2.58
spBySurveyByComptomentella.census2.4.2             -2.54
spBySurveyByCompferruginea.census1.4.3             -2.41
spBySurveyByCompmacrophylla.census1.4.3            -1.91
spBySurveyByCompparvifolia.census1.4.3             -1.26
spBySurveyByCompsangal.census1.4.3                  0.29
spBySurveyByCompferruginea.census2.4.3             -2.83
spBySurveyByCompmacrophylla.census2.4.3            -3.24
spBySurveyByCompparvifolia.census2.4.3             -2.92
spBySurveyByCompsangal.census2.4.3                 -1.52
spBySurveyByCompargentifolia.census1.4.4           -2.95
spBySurveyByCompferruginea.census1.4.4             -3.45
spBySurveyByCompparvifolia.census1.4.4             -2.04
spBySurveyByCompsangal.census1.4.4                  1.66
spBySurveyByCompargentifolia.census2.4.4           -4.15
spBySurveyByCompferruginea.census2.4.4             -3.97
spBySurveyByCompparvifolia.census2.4.4             -4.11
spBySurveyByCompsangal.census2.4.4                 -1.06
spBySurveyByCompbeccariana.census1.4.5             -0.76
spBySurveyByCompferruginea.census1.4.5             -3.89
spBySurveyByCompjohorensis.census1.4.5             -0.03
spBySurveyByCompsangal.census1.4.5                  1.80
spBySurveyByCompbeccariana.census2.4.5             -3.40
spBySurveyByCompferruginea.census2.4.5             -4.17
spBySurveyByCompjohorensis.census2.4.5             -2.97
spBySurveyByCompsangal.census2.4.5                 -1.89
spBySurveyByCompferruginea.census1.4.6             -2.86
spBySurveyByCompgibbosa.census1.4.6                -2.25
```

```
spBySurveyByCompmacroptera.census1.4.6                -0.67
spBySurveyByCompsangal.census1.4.6                     1.68
spBySurveyByCompferruginea.census2.4.6                -3.75
spBySurveyByCompgibbosa.census2.4.6                   -3.84
spBySurveyByCompmacroptera.census2.4.6                -3.16
spBySurveyByCompsangal.census2.4.6                    -1.21
spBySurveyByCompfaguetiana.census1.4.7                -2.02
spBySurveyByCompferruginea.census1.4.7                -2.32
spBySurveyByCompmacrophylla.census1.4.7               -0.08
spBySurveyByCompsangal.census1.4.7                     2.11
spBySurveyByCompfaguetiana.census2.4.7                -3.97
spBySurveyByCompferruginea.census2.4.7                -3.64
spBySurveyByCompmacrophylla.census2.4.7               -3.55
spBySurveyByCompsangal.census2.4.7                    -1.60
spBySurveyByCompargentifolia.census1.4.8              -2.72
spBySurveyByCompferruginea.census1.4.8                -2.50
spBySurveyByCompjohorensis.census1.4.8                 0.13
spBySurveyByCompsangal.census1.4.8                     1.89
spBySurveyByCompargentifolia.census2.4.8              -4.26
spBySurveyByCompferruginea.census2.4.8                -3.92
spBySurveyByCompjohorensis.census2.4.8                -2.71
spBySurveyByCompsangal.census2.4.8                    -1.09
spBySurveyByCompconformis.census1.4.9                 -0.37
spBySurveyByCompfaguetiana.census1.4.9                -2.52
spBySurveyByComplanceolata.census1.4.9                 0.31
spBySurveyByCompmalaanonan.census1.4.9                 0.32
spBySurveyByCompconformis.census2.4.9                 -3.03
spBySurveyByCompfaguetiana.census2.4.9                -4.06
spBySurveyByComplanceolata.census2.4.9                -2.40
spBySurveyByCompmalaanonan.census2.4.9                -2.60
spBySurveyByCompargentifolia.census1.argentifolia    -2.88
spBySurveyByCompargentifolia.census2.argentifolia    -4.26
spBySurveyByCompbeccariana.census1.beccariana         1.05
spBySurveyByCompbeccariana.census2.beccariana        -1.76
spBySurveyByCompconformis.census1.conformis          -0.17
spBySurveyByCompconformis.census2.conformis          -2.36
spBySurveyByCompfaguetiana.census1.faguetiana        -2.38
spBySurveyByCompfaguetiana.census2.faguetiana        -3.72
spBySurveyByCompferruginea.census1.ferruginea        -0.90
spBySurveyByCompferruginea.census2.ferruginea        -2.87
spBySurveyByCompgibbosa.census1.gibbosa              -1.60
spBySurveyByCompgibbosa.census2.gibbosa              -3.79
spBySurveyByCompjohorensis.census1.johorensis        -0.52
spBySurveyByCompjohorensis.census2.johorensis        -2.96
spBySurveyByComplanceolata.census1.lanceolata        -1.25
spBySurveyByComplanceolata.census2.lanceolata        -3.55
spBySurveyByCompleprosula.census1.leprosula          -0.97
spBySurveyByCompleprosula.census2.leprosula          -3.66
spBySurveyByCompmacrophylla.census1.macrophylla      -0.56
spBySurveyByCompmacrophylla.census2.macrophylla      -3.59
spBySurveyByCompmacroptera.census1.macroptera        -0.77
spBySurveyByCompmacroptera.census2.macroptera        -3.46
```

```
spBySurveyByCompmalaanonan.census1.malaanonan       -0.49
spBySurveyByCompmalaanonan.census2.malaanonan       -2.86
spBySurveyByCompovalis.census1.ovalis                0.69
spBySurveyByCompovalis.census2.ovalis               -2.38
spBySurveyByCompparvifolia.census1.parvifolia       -1.54
spBySurveyByCompparvifolia.census2.parvifolia       -3.96
spBySurveyByCompsangal.census1.sangal                0.96
spBySurveyByCompsangal.census2.sangal               -2.46
spBySurveyByCompargentifolia.census1.sixteen        -3.23
spBySurveyByCompbeccariana.census1.sixteen          -0.73
spBySurveyByCompconformis.census1.sixteen            0.17
spBySurveyByCompfaguetiana.census1.sixteen          -2.95
spBySurveyByCompferruginea.census1.sixteen          -3.14
spBySurveyByCompgibbosa.census1.sixteen             -2.87
spBySurveyByCompjohorensis.census1.sixteen           0.16
spBySurveyByComplanceolata.census1.sixteen          -0.11
spBySurveyByCompleprosula.census1.sixteen           -1.56
spBySurveyByCompmacrophylla.census1.sixteen         -2.35
spBySurveyByCompmacroptera.census1.sixteen          -0.96
spBySurveyByCompmalaanonan.census1.sixteen          -0.20
spBySurveyByCompovalis.census1.sixteen               0.79
spBySurveyByCompparvifolia.census1.sixteen          -2.22
spBySurveyByCompsangal.census1.sixteen               1.96
spBySurveyByComptomentella.census1.sixteen           0.46
spBySurveyByCompargentifolia.census2.sixteen        -5.67
spBySurveyByCompbeccariana.census2.sixteen          -4.13
spBySurveyByCompconformis.census2.sixteen           -3.63
spBySurveyByCompfaguetiana.census2.sixteen          -5.45
spBySurveyByCompferruginea.census2.sixteen          -4.99
spBySurveyByCompgibbosa.census2.sixteen             -5.10
spBySurveyByCompjohorensis.census2.sixteen          -3.88
spBySurveyByComplanceolata.census2.sixteen          -3.85
spBySurveyByCompleprosula.census2.sixteen           -5.17
spBySurveyByCompmacrophylla.census2.sixteen         -5.16
spBySurveyByCompmacroptera.census2.sixteen          -4.65
spBySurveyByCompmalaanonan.census2.sixteen          -3.84
spBySurveyByCompovalis.census2.sixteen              -3.50
spBySurveyByCompparvifolia.census2.sixteen          -5.32
spBySurveyByCompsangal.census2.sixteen              -2.47
spBySurveyByComptomentella.census2.sixteen          -3.39
spBySurveyByComptomentella.census1.tomentella        0.24
spBySurveyByComptomentella.census2.tomentella       -2.84


Correlation matrix not shown by default, as p = 192 > 12.
Use print(x, correlation=TRUE)  or
vcov(x) if you need it
```

```
VarCorr(survivalModel)
```

```
 Groups   Name        Std.Dev.
 sp.pl    (Intercept) 0.0751
```

```
Residual             0.0667
```

5.2 Survival residual diagnostics

The residual plots show a hard boundary against 0, but overall they look good.

```
plot(survivalModel)
```

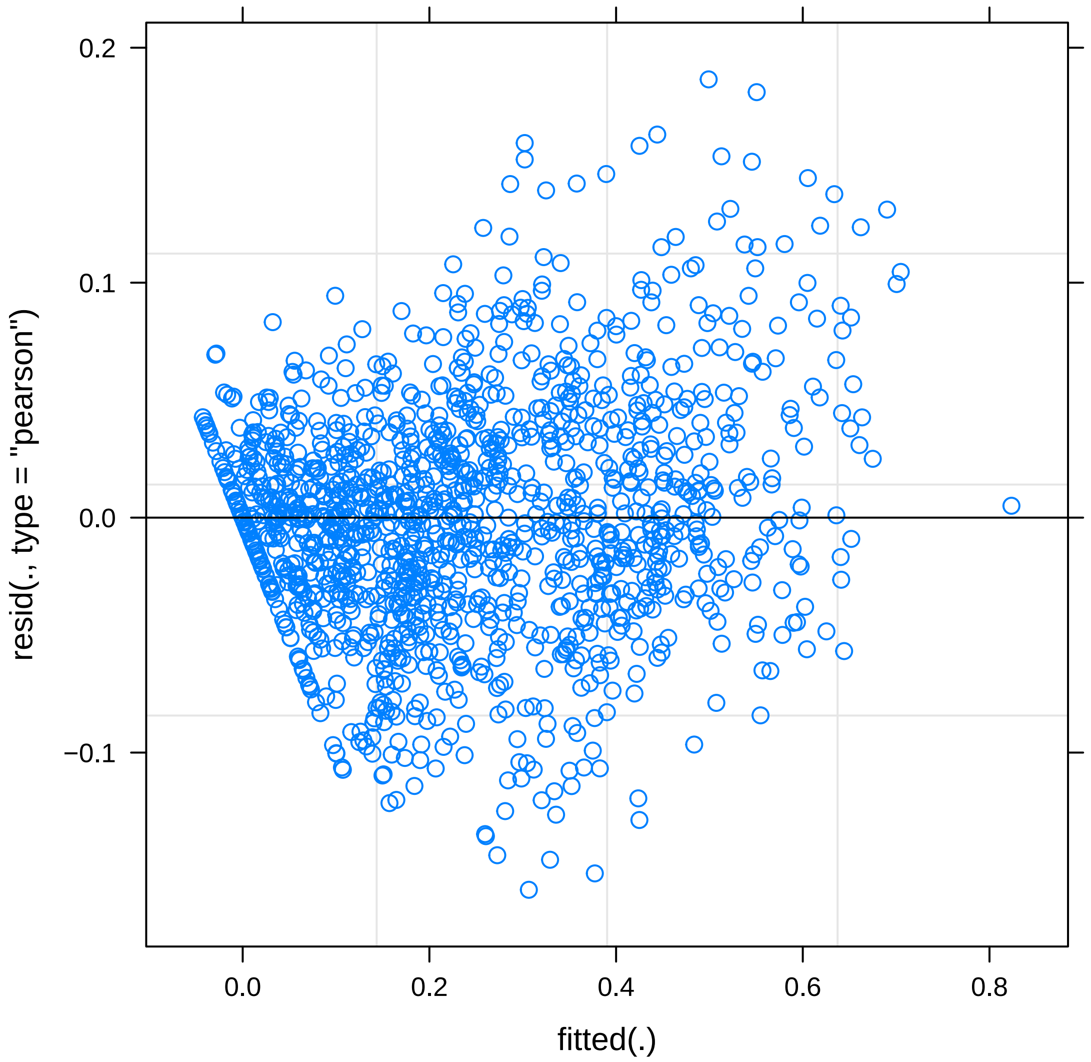

Here with the arcsine square root transformation.

```
plot(lmer(asin(sqrt(survival)) ~ spBySurveyByComp + (1 | sp.pl), data = survival))
```

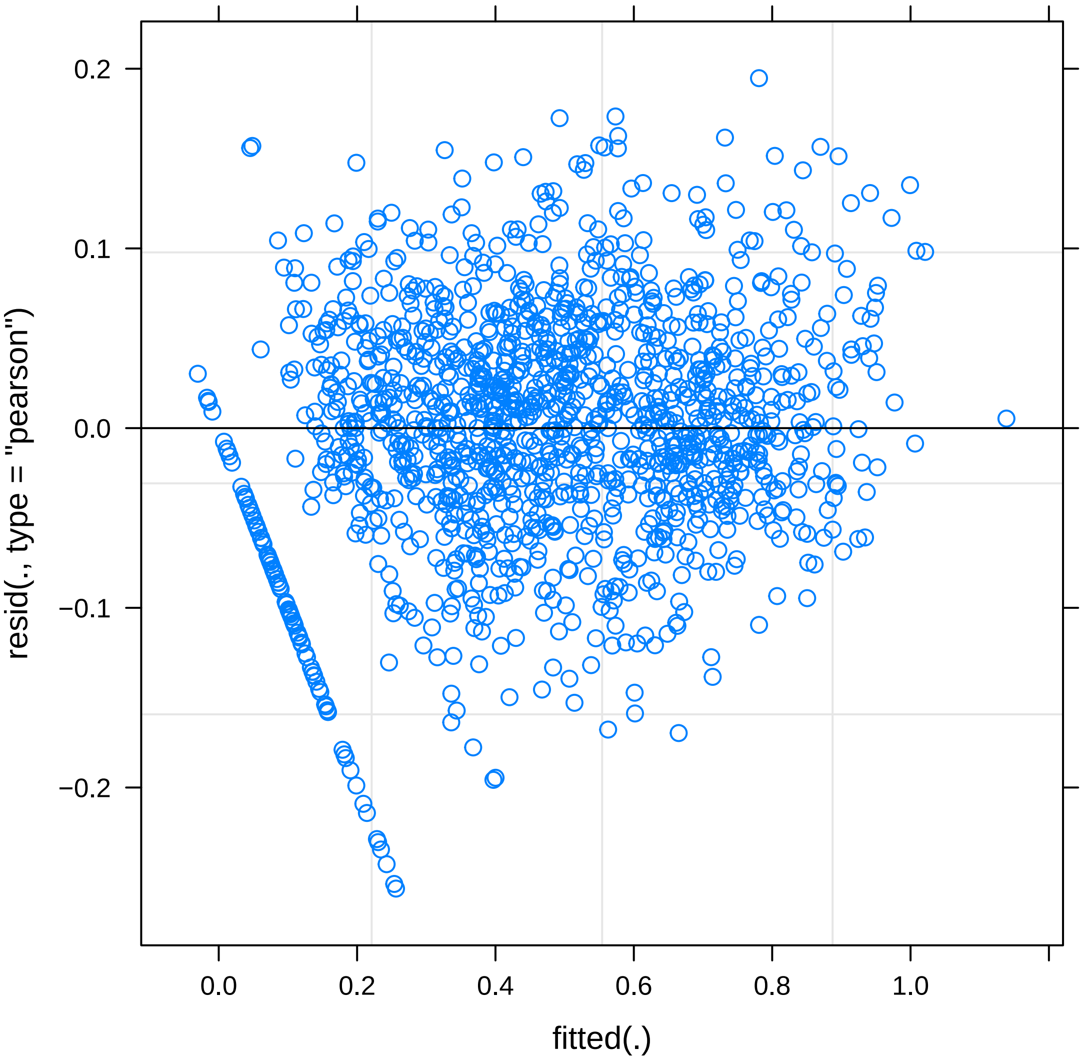

No real difference, so transformation not necessary.

```
f.diagno(survivalModel)
```

survival‘~‘spBySurveyByComp + (1 | sp.pl)

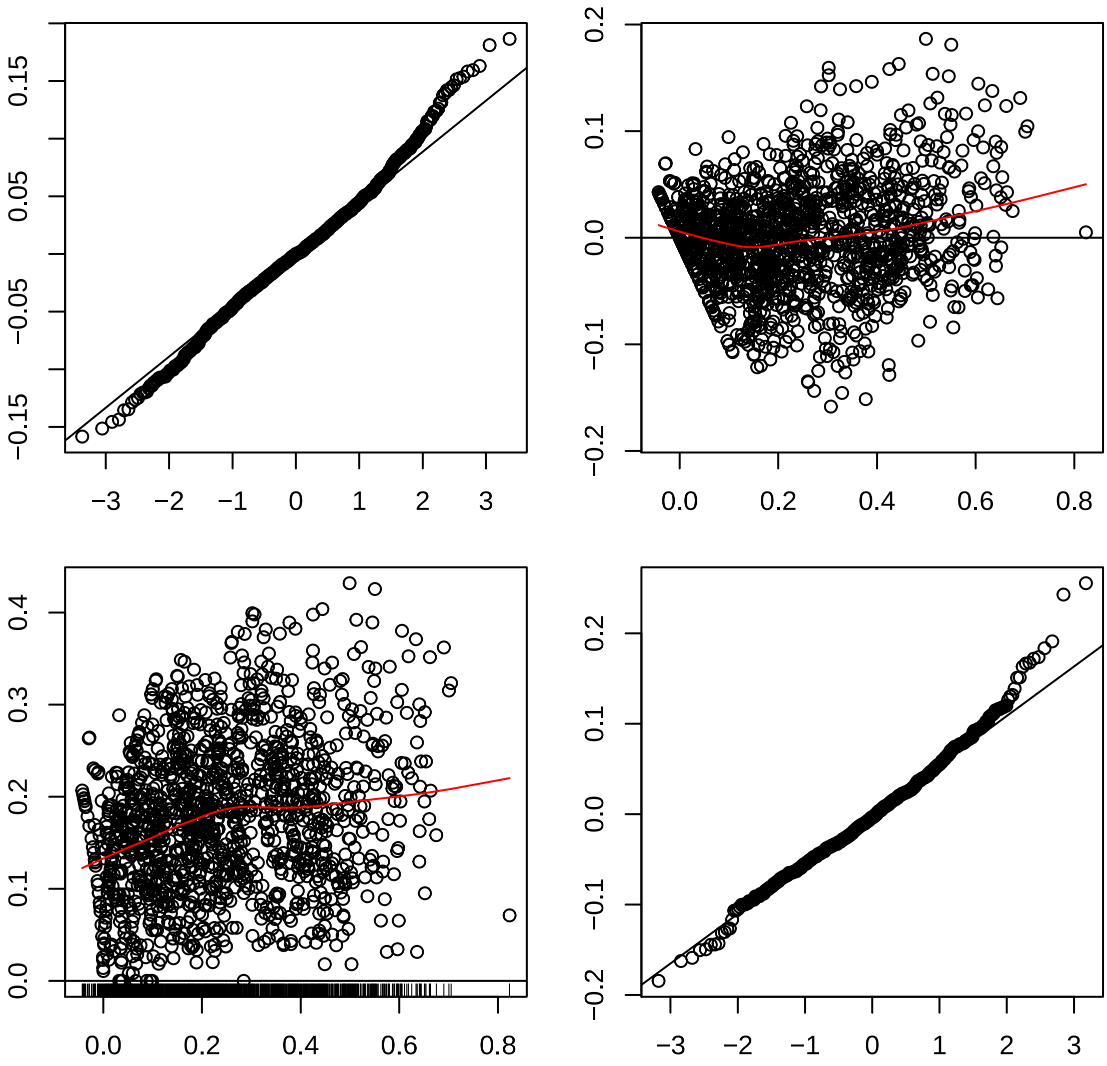

Diagnostics look pretty good!

```r
f.good(survivalModel)
```

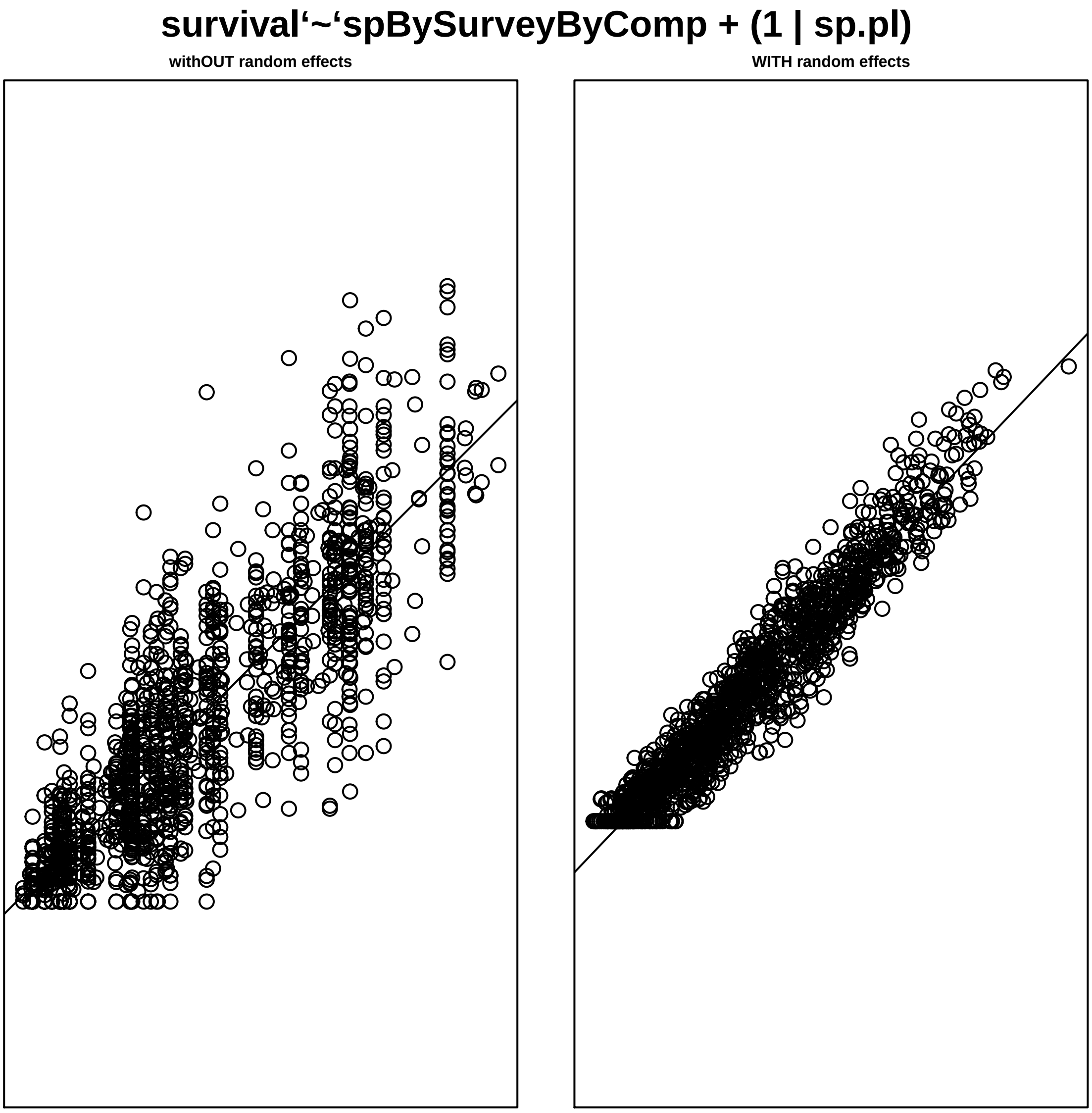

The goodness of fit looks good (better than growth model below), and random effects make a big difference.

What about spatial correlation?

```r
plres2x(residuals(survivalModel) ~ X + Y, data = survival, asp = 1)
```

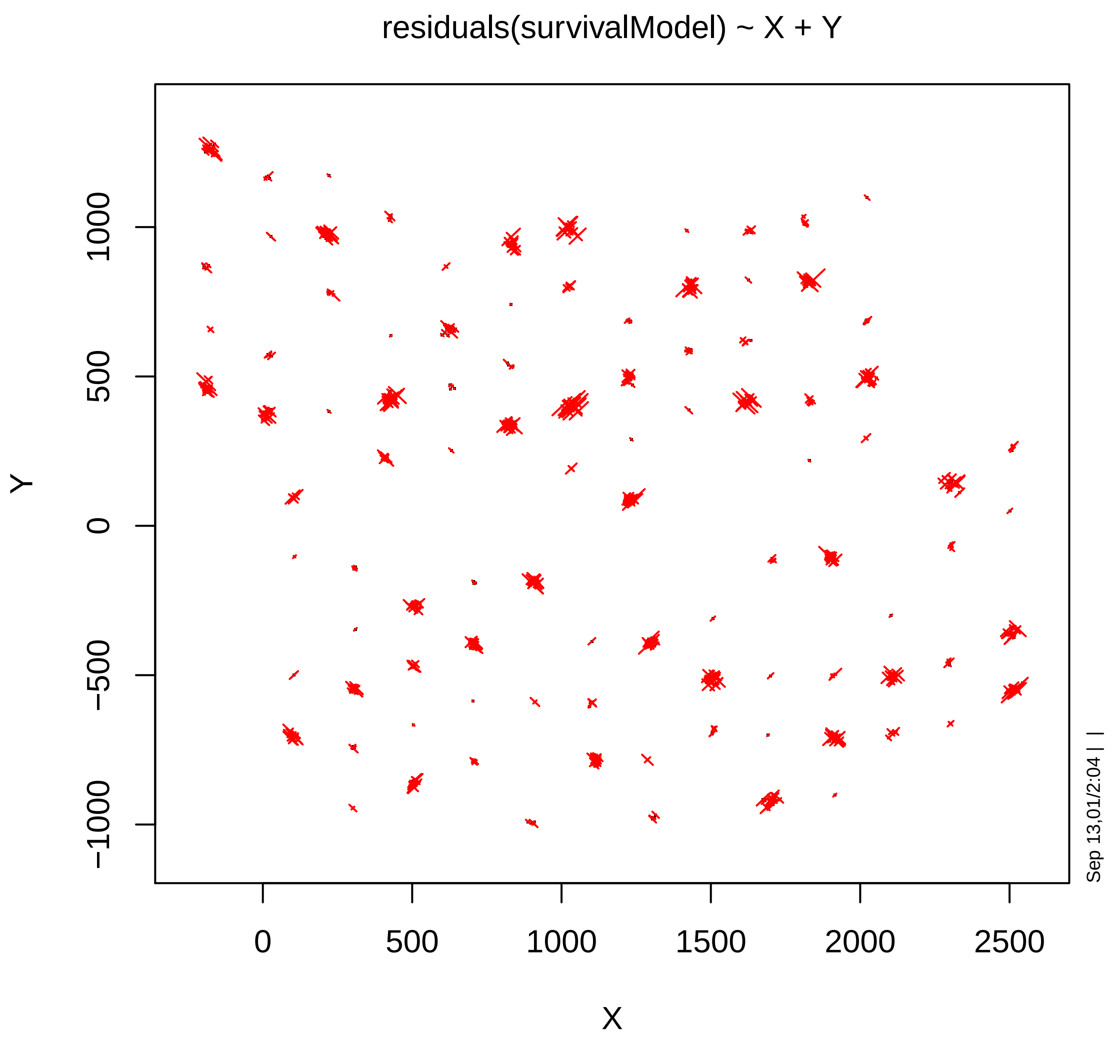


```
[1] "plres2x done"

plot(variog(data = residuals(survivalModel), coords = survival[, c("X", "Y")],
    max.dist = 500), pch = 20, main = "Spatial correlation of survival model residuals")

variog: computing omnidirectional variogram
variog: co-locatted data found, adding one bin at the origin
```

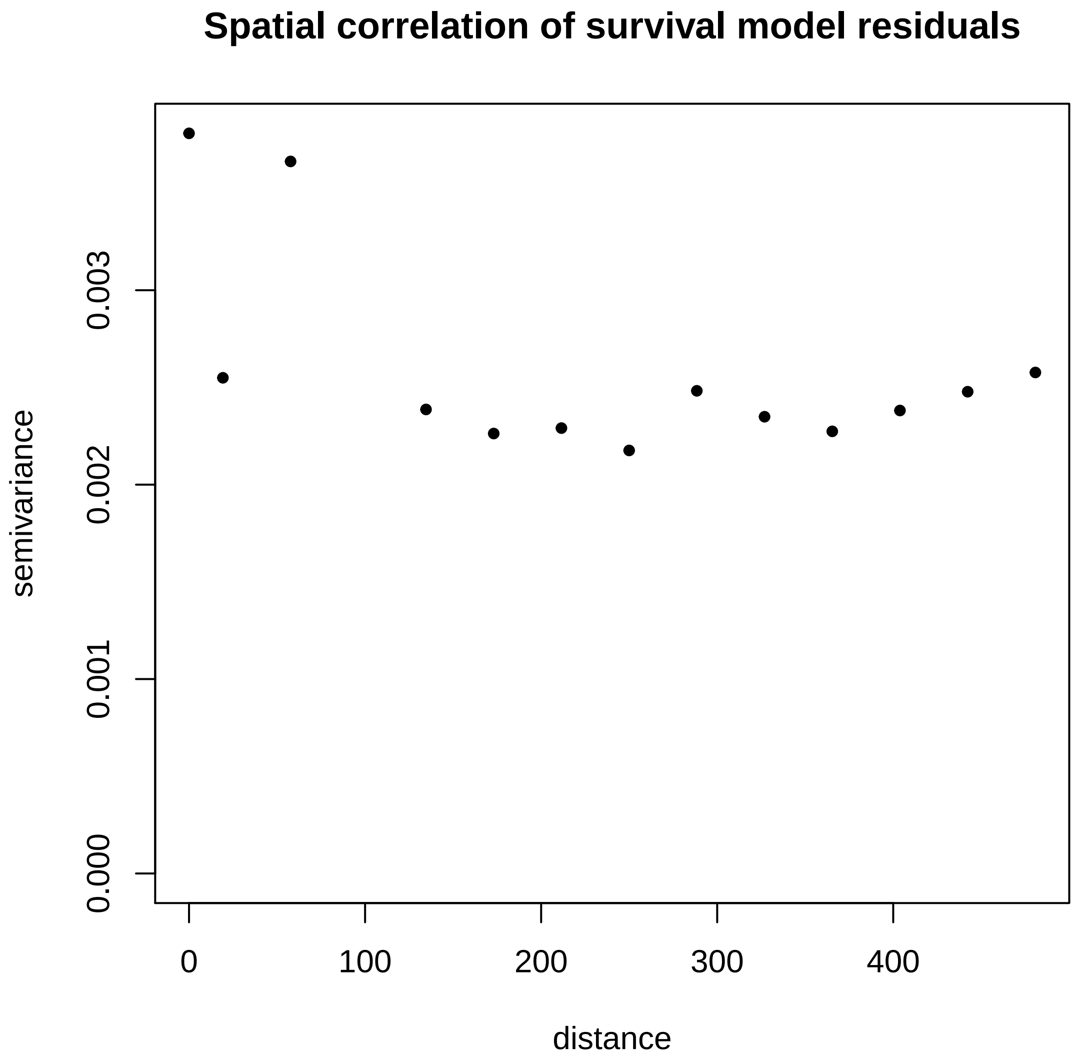

5.3 Fit the model for growth

```
growthModel <- lmer(mean.log.diam ~ SP.sp.comp * mdsp.sc + (1 | sp.pl),
                    data = growth)
summary(growthModel)

Linear mixed model fit by REML ['lmerMod']
Formula: mean.log.diam ~ SP.sp.comp * mdsp.sc + (1 | sp.pl)
   Data: growth

REML criterion at convergence: -626

Scaled residuals:
   Min     1Q Median     3Q    Max
-4.020 -0.380 -0.001  0.344  5.011

Random effects:
 Groups   Name        Variance Std.Dev.
```

```
 sp.pl    (Intercept) 0.0095   0.0975  
 Residual             0.0135   0.1161  
Number of obs: 1122, groups:  sp.pl, 561

Fixed effects:
                                            Estimate Std. Error t value
(Intercept)                                 1.039648   0.090121   11.54
SP.sp.compleprosula.4.1                    -0.010784   0.127450   -0.08
SP.sp.compmalaanonan.4.1                   -0.033840   0.127450   -0.27
SP.sp.comptomentella.4.1                   -0.026220   0.127450   -0.21
SP.sp.compconformis.4.10                   -0.115748   0.127449   -0.91
SP.sp.compjohorensis.4.10                  -0.035354   0.127450   -0.28
SP.sp.complanceolata.4.10                   0.009785   0.127452    0.08
SP.sp.comptomentella.4.10                  -0.058659   0.127454   -0.46
SP.sp.compconformis.4.11                   -0.034779   0.127450   -0.27
SP.sp.compgibbosa.4.11                     -0.069696   0.127450   -0.55
SP.sp.complanceolata.4.11                  -0.013363   0.127450   -0.10
SP.sp.comptomentella.4.11                   0.012346   0.127450    0.10
SP.sp.compconformis.4.12                   -0.243324   0.156097   -1.56
SP.sp.compjohorensis.4.12                  -0.058503   0.156096   -0.37
SP.sp.complanceolata.4.12                  -0.110061   0.156096   -0.71
SP.sp.compmalaanonan.4.12                  -0.038364   0.156094   -0.25
SP.sp.compconformis.4.13                    0.056287   0.127472    0.44
SP.sp.complanceolata.4.13                   0.171718   0.127474    1.35
SP.sp.compmacrophylla.4.13                  0.349573   0.127471    2.74
SP.sp.compsangal.4.13                       0.126923   0.127465    1.00
SP.sp.compconformis.4.14                   -0.063970   0.127467   -0.50
SP.sp.compferruginea.4.14                  -0.113200   0.127489   -0.89
SP.sp.complanceolata.4.14                   0.039990   0.127467    0.31
SP.sp.compovalis.4.14                       0.004721   0.127467    0.04
SP.sp.compconformis.4.15                   -0.116855   0.127516   -0.92
SP.sp.complanceolata.4.15                  -0.032861   0.127516   -0.26
SP.sp.compovalis.4.15                       0.030937   0.127553    0.24
SP.sp.compsangal.4.15                      -0.050544   0.127516   -0.40
SP.sp.compconformis.4.16                   -0.050329   0.127470   -0.39
SP.sp.compferruginea.4.16                  -0.107199   0.127470   -0.84
SP.sp.complanceolata.4.16                   0.019927   0.127470    0.16
SP.sp.compmacrophylla.4.16                  0.174980   0.127470    1.37
SP.sp.compmacroptera.4.2                   -0.078997   0.127451   -0.62
SP.sp.compmalaanonan.4.2                   -0.041335   0.127451   -0.32
SP.sp.compovalis.4.2                        0.047016   0.127451    0.37
SP.sp.comptomentella.4.2                   -0.078872   0.127456   -0.62
SP.sp.compferruginea.4.3                   -0.153216   0.156242   -0.98
SP.sp.compmacrophylla.4.3                   0.222522   0.156278    1.42
SP.sp.compparvifolia.4.3                    0.091815   0.156281    0.59
SP.sp.compsangal.4.3                        0.002680   0.156282    0.02
SP.sp.compargentifolia.4.4                  0.291415   0.127478    2.29
SP.sp.compferruginea.4.4                   -0.154441   0.127478   -1.21
SP.sp.compparvifolia.4.4                   -0.058923   0.127478   -0.46
SP.sp.compsangal.4.4                       -0.083714   0.127478   -0.66
SP.sp.compbeccariana.4.5                    0.008268   0.127464    0.06
SP.sp.compferruginea.4.5                   -0.142442   0.156094   -0.91
```

```
SP.sp.compjohorensis.4.5                      0.014050   0.127458    0.11
SP.sp.compsangal.4.5                          0.013836   0.127455    0.11
SP.sp.compferruginea.4.6                     -0.107789   0.127453   -0.85
SP.sp.compgibbosa.4.6                        -0.028746   0.127452   -0.23
SP.sp.compmacroptera.4.6                      0.000227   0.127452    0.00
SP.sp.compsangal.4.6                          0.040098   0.127452    0.31
SP.sp.compfaguetiana.4.7                     -0.064075   0.127450   -0.50
SP.sp.compferruginea.4.7                     -0.254934   0.127450   -2.00
SP.sp.compmacrophylla.4.7                     0.146414   0.127450    1.15
SP.sp.compsangal.4.7                         -0.068528   0.127450   -0.54
SP.sp.compargentifolia.4.8                    0.127132   0.127467    1.00
SP.sp.compferruginea.4.8                     -0.154280   0.127467   -1.21
SP.sp.compjohorensis.4.8                      0.073771   0.127467    0.58
SP.sp.compsangal.4.8                          0.024825   0.127467    0.19
SP.sp.compconformis.4.9                      -0.126529   0.127449   -0.99
SP.sp.compfaguetiana.4.9                     -0.066812   0.127450   -0.52
SP.sp.complanceolata.4.9                     -0.078370   0.127450   -0.61
SP.sp.compmalaanonan.4.9                     -0.077753   0.127450   -0.61
SP.sp.compargentifolia.argentifolia           0.195547   0.156187    1.25
SP.sp.compbeccariana.beccariana               0.075338   0.127451    0.59
SP.sp.compconformis.conformis                -0.073998   0.127486   -0.58
SP.sp.compfaguetiana.faguetiana              -0.019307   0.127449   -0.15
SP.sp.compferruginea.ferruginea              -0.144638   0.127561   -1.13
SP.sp.compgibbosa.gibbosa                    -0.017262   0.127450   -0.14
SP.sp.compjohorensis.johorensis               0.011166   0.127512    0.09
SP.sp.complanceolata.lanceolata               0.045627   0.127478    0.36
SP.sp.compleprosula.leprosula                 0.048369   0.127450    0.38
SP.sp.compmacrophylla.macrophylla             0.082047   0.127450    0.64
SP.sp.compmacroptera.macroptera               0.020917   0.127492    0.16
SP.sp.compmalaanonan.malaanonan               0.008567   0.127459    0.07
SP.sp.compovalis.ovalis                       0.108501   0.127455    0.85
SP.sp.compparvifolia.parvifolia               0.101944   0.127451    0.80
SP.sp.compsangal.sangal                      -0.004259   0.156289   -0.03
SP.sp.compargentifolia.sixteen                0.129306   0.096343    1.34
SP.sp.compbeccariana.sixteen                  0.025895   0.093076    0.28
SP.sp.compconformis.sixteen                  -0.039221   0.093076   -0.42
SP.sp.compfaguetiana.sixteen                  0.048977   0.094996    0.52
SP.sp.compferruginea.sixteen                 -0.115613   0.093656   -1.23
SP.sp.compgibbosa.sixteen                     0.010680   0.093656    0.11
SP.sp.compjohorensis.sixteen                  0.082663   0.093177    0.89
SP.sp.complanceolata.sixteen                  0.009148   0.092983    0.10
SP.sp.compleprosula.sixteen                   0.175978   0.094520    1.86
SP.sp.compmacrophylla.sixteen                 0.192169   0.094315    2.04
SP.sp.compmacroptera.sixteen                  0.015282   0.093284    0.16
SP.sp.compmalaanonan.sixteen                  0.001306   0.093076    0.01
SP.sp.compovalis.sixteen                      0.052330   0.092983    0.56
SP.sp.compparvifolia.sixteen                  0.070160   0.094520    0.74
SP.sp.compsangal.sixteen                     -0.004832   0.092983   -0.05
SP.sp.comptomentella.sixteen                  0.015393   0.093177    0.17
SP.sp.comptomentella.tomentella              -0.040138   0.127454   -0.31
mdsp.sc                                       0.214684   0.056856    3.78
SP.sp.compleprosula.4.1:mdsp.sc              -0.058585   0.080422   -0.73
```

```
SP.sp.compmalaanonan.4.1:mdsp.sc          -0.088309   0.080402   -1.10
SP.sp.comptomentella.4.1:mdsp.sc          -0.056007   0.080405   -0.70
SP.sp.compconformis.4.10:mdsp.sc          -0.052650   0.081010   -0.65
SP.sp.compjohorensis.4.10:mdsp.sc         -0.006601   0.080735   -0.08
SP.sp.complanceolata.4.10:mdsp.sc         -0.012838   0.080418   -0.16
SP.sp.comptomentella.4.10:mdsp.sc         -0.060116   0.080402   -0.75
SP.sp.compconformis.4.11:mdsp.sc          -0.029529   0.079581   -0.37
SP.sp.compgibbosa.4.11:mdsp.sc            -0.053058   0.079593   -0.67
SP.sp.complanceolata.4.11:mdsp.sc          0.004385   0.079592    0.06
SP.sp.comptomentella.4.11:mdsp.sc         -0.031295   0.079588   -0.39
SP.sp.compconformis.4.12:mdsp.sc          -0.126616   0.099554   -1.27
SP.sp.compjohorensis.4.12:mdsp.sc         -0.012318   0.099649   -0.12
SP.sp.complanceolata.4.12:mdsp.sc         -0.026971   0.099707   -0.27
SP.sp.compmalaanonan.4.12:mdsp.sc          0.003458   0.099887    0.03
SP.sp.compconformis.4.13:mdsp.sc          -0.013567   0.080684   -0.17
SP.sp.complanceolata.4.13:mdsp.sc          0.085186   0.080451    1.06
SP.sp.compmacrophylla.4.13:mdsp.sc         0.040344   0.080650    0.50
SP.sp.compsangal.4.13:mdsp.sc              0.062892   0.080685    0.78
SP.sp.compconformis.4.14:mdsp.sc          -0.027680   0.080980   -0.34
SP.sp.compferruginea.4.14:mdsp.sc          0.104189   0.081729    1.27
SP.sp.complanceolata.4.14:mdsp.sc         -0.002209   0.080979   -0.03
SP.sp.compovalis.4.14:mdsp.sc             -0.017970   0.080980   -0.22
SP.sp.compconformis.4.15:mdsp.sc          -0.096594   0.079750   -1.21
SP.sp.complanceolata.4.15:mdsp.sc          0.010698   0.079752    0.13
SP.sp.compovalis.4.15:mdsp.sc             -0.025139   0.080413   -0.31
SP.sp.compsangal.4.15:mdsp.sc             -0.032046   0.079747   -0.40
SP.sp.compconformis.4.16:mdsp.sc          -0.058118   0.080062   -0.73
SP.sp.compferruginea.4.16:mdsp.sc          0.149737   0.080055    1.87
SP.sp.complanceolata.4.16:mdsp.sc          0.026999   0.080051    0.34
SP.sp.compmacrophylla.4.16:mdsp.sc        -0.033075   0.080049   -0.41
SP.sp.compmacroptera.4.2:mdsp.sc          -0.060535   0.082935   -0.73
SP.sp.compmalaanonan.4.2:mdsp.sc          -0.054437   0.082930   -0.66
SP.sp.compovalis.4.2:mdsp.sc              -0.034103   0.082930   -0.41
SP.sp.comptomentella.4.2:mdsp.sc          -0.055196   0.083393   -0.66
SP.sp.compferruginea.4.3:mdsp.sc           0.063363   0.100107    0.63
SP.sp.compmacrophylla.4.3:mdsp.sc          0.016589   0.098971    0.17
SP.sp.compparvifolia.4.3:mdsp.sc           0.098555   0.099194    0.99
SP.sp.compsangal.4.3:mdsp.sc              -0.002062   0.099092   -0.02
SP.sp.compargentifolia.4.4:mdsp.sc         0.034983   0.083419    0.42
SP.sp.compferruginea.4.4:mdsp.sc          -0.002746   0.083419   -0.03
SP.sp.compparvifolia.4.4:mdsp.sc          -0.050182   0.083426   -0.60
SP.sp.compsangal.4.4:mdsp.sc              -0.044065   0.083419   -0.53
SP.sp.compbeccariana.4.5:mdsp.sc           0.028238   0.082946    0.34
SP.sp.compferruginea.4.5:mdsp.sc           0.148306   0.098794    1.50
SP.sp.compjohorensis.4.5:mdsp.sc          -0.019718   0.082609   -0.24
SP.sp.compsangal.4.5:mdsp.sc              -0.016580   0.082429   -0.20
SP.sp.compferruginea.4.6:mdsp.sc           0.105618   0.083456    1.27
SP.sp.compgibbosa.4.6:mdsp.sc              0.026885   0.083262    0.32
SP.sp.compmacroptera.4.6:mdsp.sc           0.005388   0.083313    0.06
SP.sp.compsangal.4.6:mdsp.sc               0.029727   0.083473    0.36
SP.sp.compfaguetiana.4.7:mdsp.sc          -0.020994   0.082680   -0.25
SP.sp.compferruginea.4.7:mdsp.sc           0.049548   0.082700    0.60
```

```
SP.sp.compmacrophylla.4.7:mdsp.sc           -0.022621   0.082689   -0.27
SP.sp.compsangal.4.7:mdsp.sc                -0.006511   0.082691   -0.08
SP.sp.compargentifolia.4.8:mdsp.sc           0.005729   0.083376    0.07
SP.sp.compferruginea.4.8:mdsp.sc             0.086145   0.083380    1.03
SP.sp.compjohorensis.4.8:mdsp.sc             0.027148   0.083379    0.33
SP.sp.compsangal.4.8:mdsp.sc                -0.002481   0.083377   -0.03
SP.sp.compconformis.4.9:mdsp.sc             -0.076961   0.083439   -0.92
SP.sp.compfaguetiana.4.9:mdsp.sc             0.001729   0.083127    0.02
SP.sp.complanceolata.4.9:mdsp.sc            -0.035189   0.083128   -0.42
SP.sp.compmalaanonan.4.9:mdsp.sc            -0.079419   0.083127   -0.96
SP.sp.compargentifolia.argentifolia:mdsp.sc  0.045504   0.101155    0.45
SP.sp.compbeccariana.beccariana:mdsp.sc      0.074226   0.081230    0.91
SP.sp.compconformis.conformis:mdsp.sc       -0.032972   0.083482   -0.39
SP.sp.compfaguetiana.faguetiana:mdsp.sc      0.033091   0.082979    0.40
SP.sp.compferruginea.ferruginea:mdsp.sc     -0.003496   0.083174   -0.04
SP.sp.compgibbosa.gibbosa:mdsp.sc            0.029363   0.079498    0.37
SP.sp.compjohorensis.johorensis:mdsp.sc     -0.047436   0.079847   -0.59
SP.sp.complanceolata.lanceolata:mdsp.sc      0.021284   0.080240    0.27
SP.sp.compleprosula.leprosula:mdsp.sc       -0.004931   0.080219   -0.06
SP.sp.compmacrophylla.macrophylla:mdsp.sc   -0.089152   0.081516   -1.09
SP.sp.compmacroptera.macroptera:mdsp.sc     -0.013507   0.080403   -0.17
SP.sp.compmalaanonan.malaanonan:mdsp.sc     -0.015756   0.083132   -0.19
SP.sp.compovalis.ovalis:mdsp.sc             -0.008001   0.080594   -0.10
SP.sp.compparvifolia.parvifolia:mdsp.sc      0.033346   0.082973    0.40
SP.sp.compsangal.sangal:mdsp.sc              0.016672   0.101008    0.17
SP.sp.compargentifolia.sixteen:mdsp.sc       0.124890   0.061086    2.04
SP.sp.compbeccariana.sixteen:mdsp.sc         0.035496   0.058806    0.60
SP.sp.compconformis.sixteen:mdsp.sc         -0.026076   0.058805   -0.44
SP.sp.compfaguetiana.sixteen:mdsp.sc         0.036013   0.060023    0.60
SP.sp.compferruginea.sixteen:mdsp.sc         0.078738   0.059193    1.33
SP.sp.compgibbosa.sixteen:mdsp.sc            0.039095   0.059167    0.66
SP.sp.compjohorensis.sixteen:mdsp.sc         0.029259   0.058873    0.50
SP.sp.complanceolata.sixteen:mdsp.sc         0.026010   0.058735    0.44
SP.sp.compleprosula.sixteen:mdsp.sc          0.081508   0.059705    1.37
SP.sp.compmacrophylla.sixteen:mdsp.sc        0.008452   0.059592    0.14
SP.sp.compmacroptera.sixteen:mdsp.sc         0.016290   0.058926    0.28
SP.sp.compmalaanonan.sixteen:mdsp.sc        -0.021862   0.058785   -0.37
SP.sp.compovalis.sixteen:mdsp.sc            -0.011205   0.058744   -0.19
SP.sp.compparvifolia.sixteen:mdsp.sc         0.054761   0.059770    0.92
SP.sp.compsangal.sixteen:mdsp.sc             0.015259   0.058742    0.26
SP.sp.comptomentella.sixteen:mdsp.sc        -0.041576   0.058888   -0.71
SP.sp.comptomentella.tomentella:mdsp.sc     -0.065455   0.080106   -0.82


Correlation matrix not shown by default, as p = 192 > 12.
Use print(x, correlation=TRUE)  or
vcov(x) if you need it
```

```
VarCorr(growthModel)
```

```
 Groups   Name        Std.Dev.
 sp.pl    (Intercept) 0.0975
```

```
Residual              0.1161
```

5.3.1 Model fit plot

Predicted growth slopes from the model:

```
xyplot(predict(growthModel, re.form = NA) ~ mean.daysSincePlanting | sp,
       data = growth,
       type = c("p","r","g"),
       groups = sp.comp,
       scales = list(y = list(at = NULL),  #relation="free",
                     x = list(rot = 45)),
       between = list(x = 0),
       cex = 0.3) #, auto.key=list(space="right"))
```

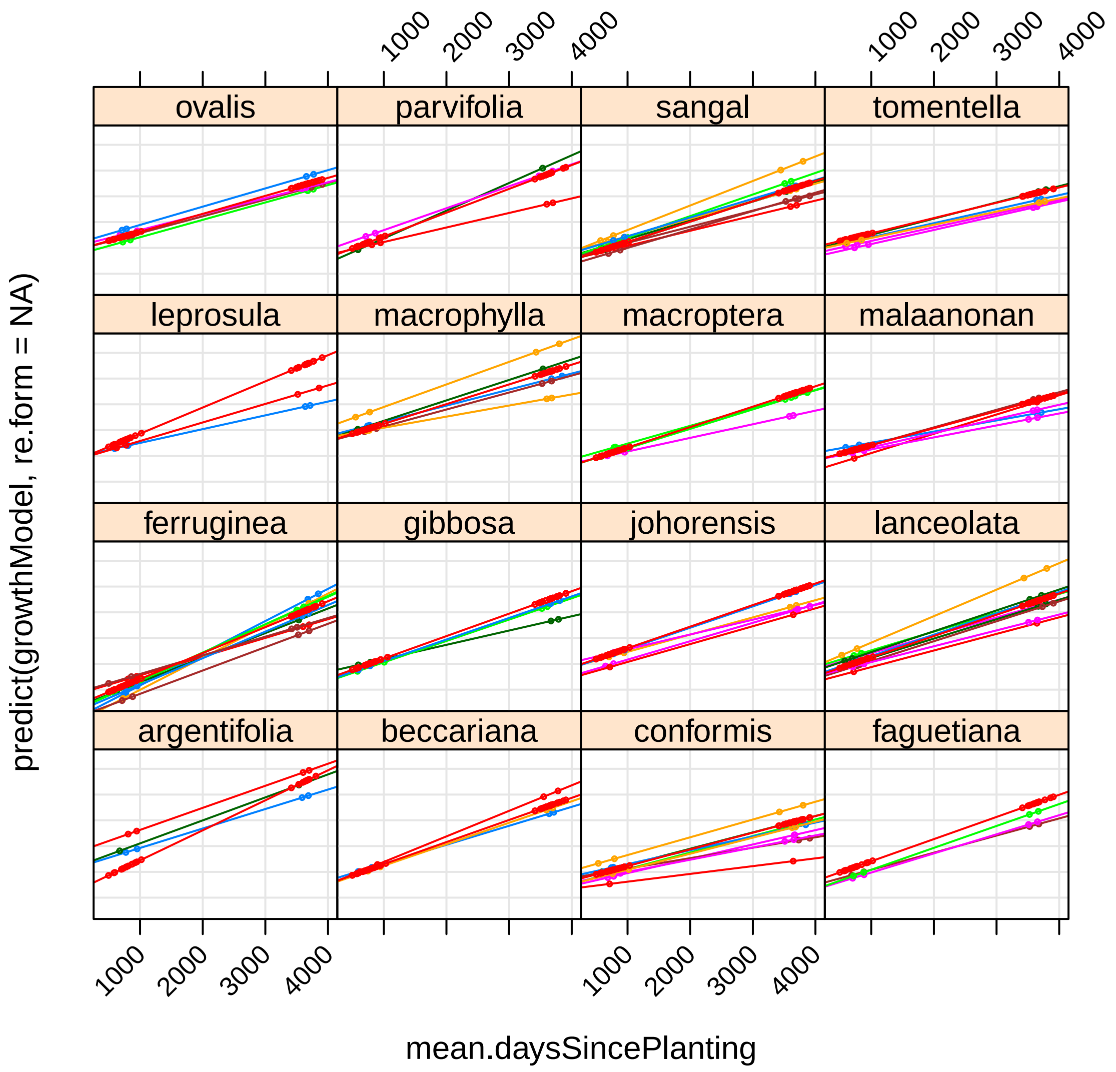

5.4 Growth residual diagnostics

```
f.diagno(growthModel)
```

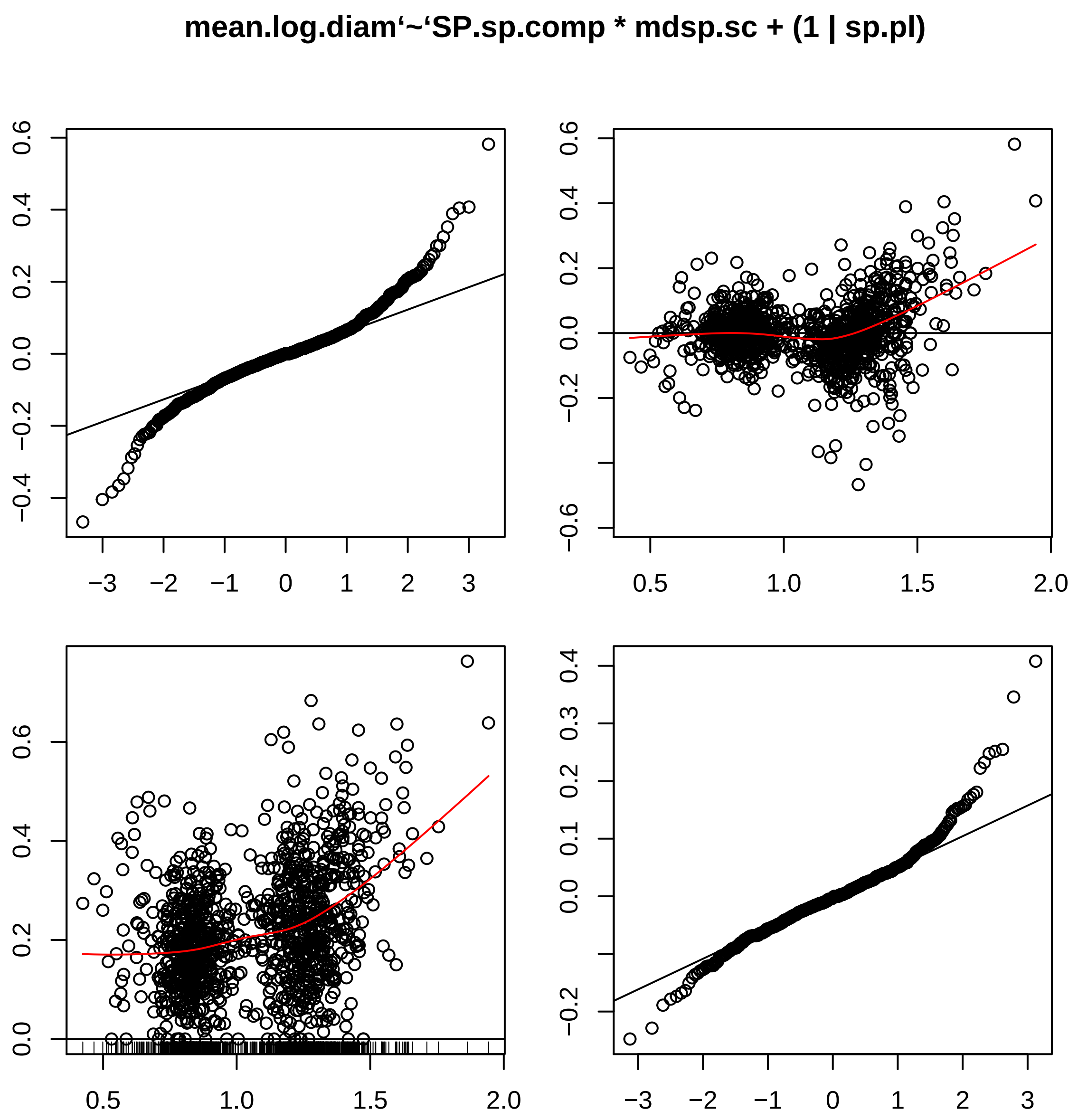

Errors are slightly longtailed, but pretty much symmetric.

```r
qqnorm(unlist(ranef(growthModel)), main = "sp.pl (int)")
qqline(unlist(ranef(growthModel)))
```

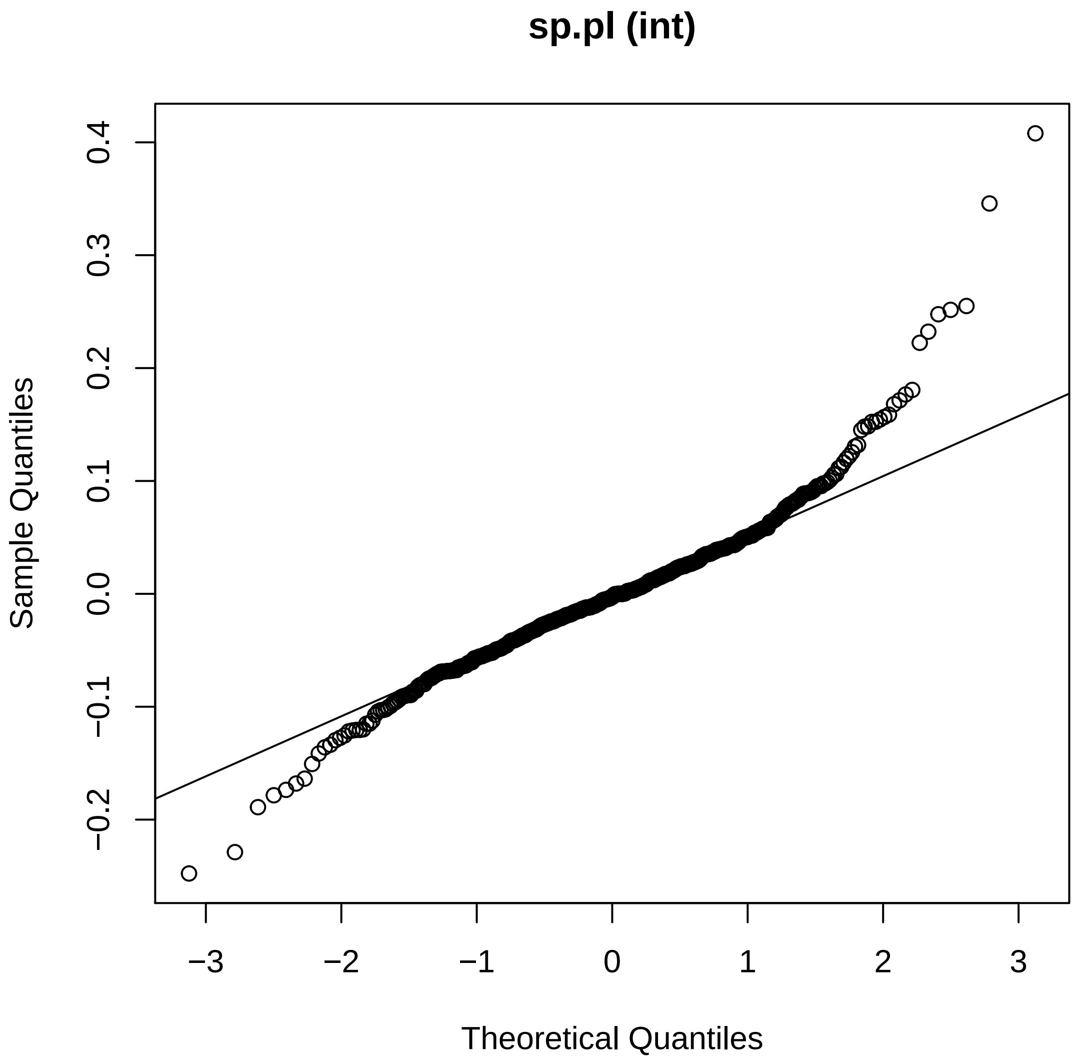

Both random intercepts are a bit right-skewed, the random slopes are a bit long-tailed and right-skewed.

```
f.good(growthModel)
```

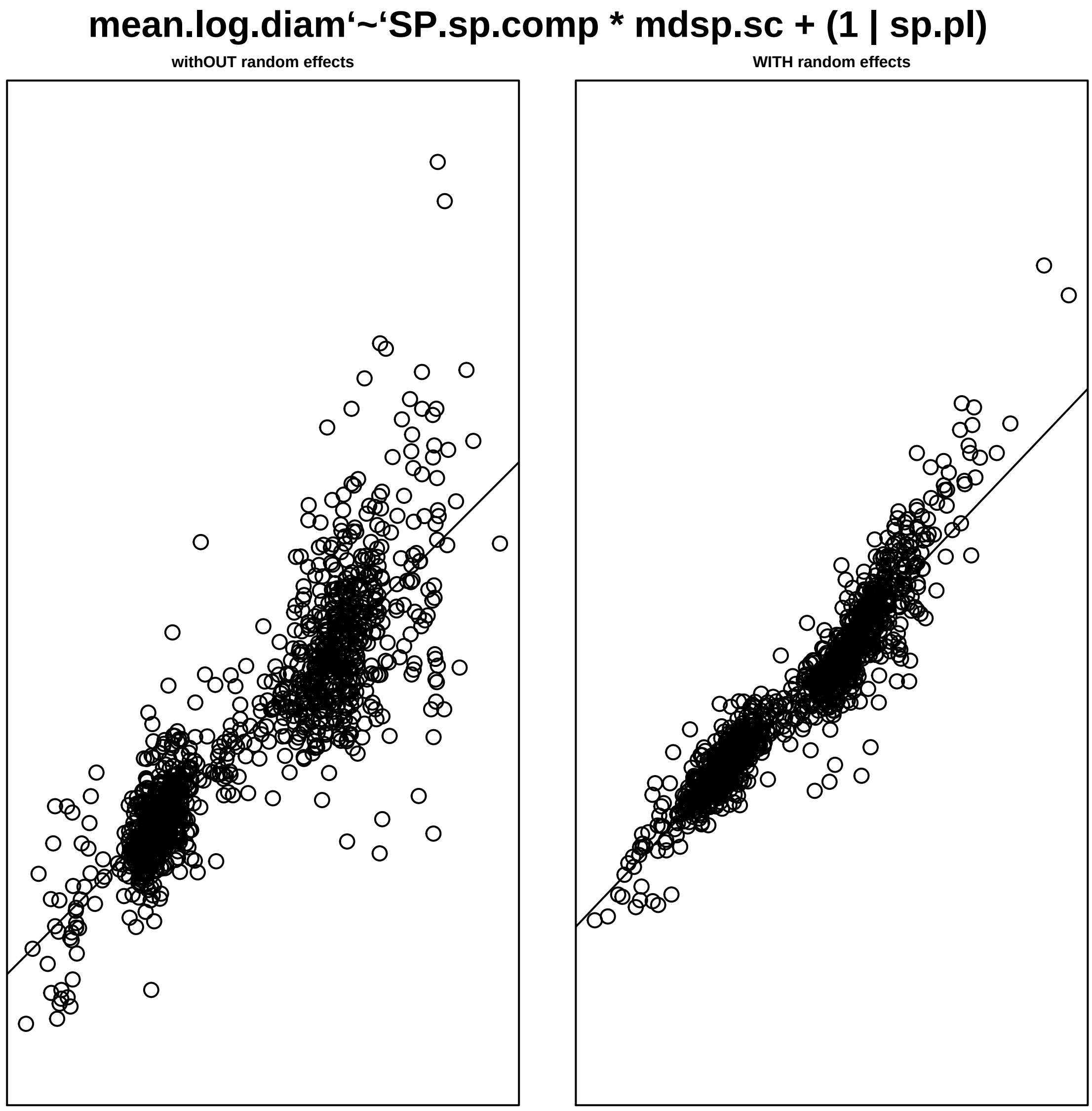

What about spatial correlation?

```
plres2x(residuals(growthModel) ~ mean.X + mean.Y, data = growth, asp = 1)
```

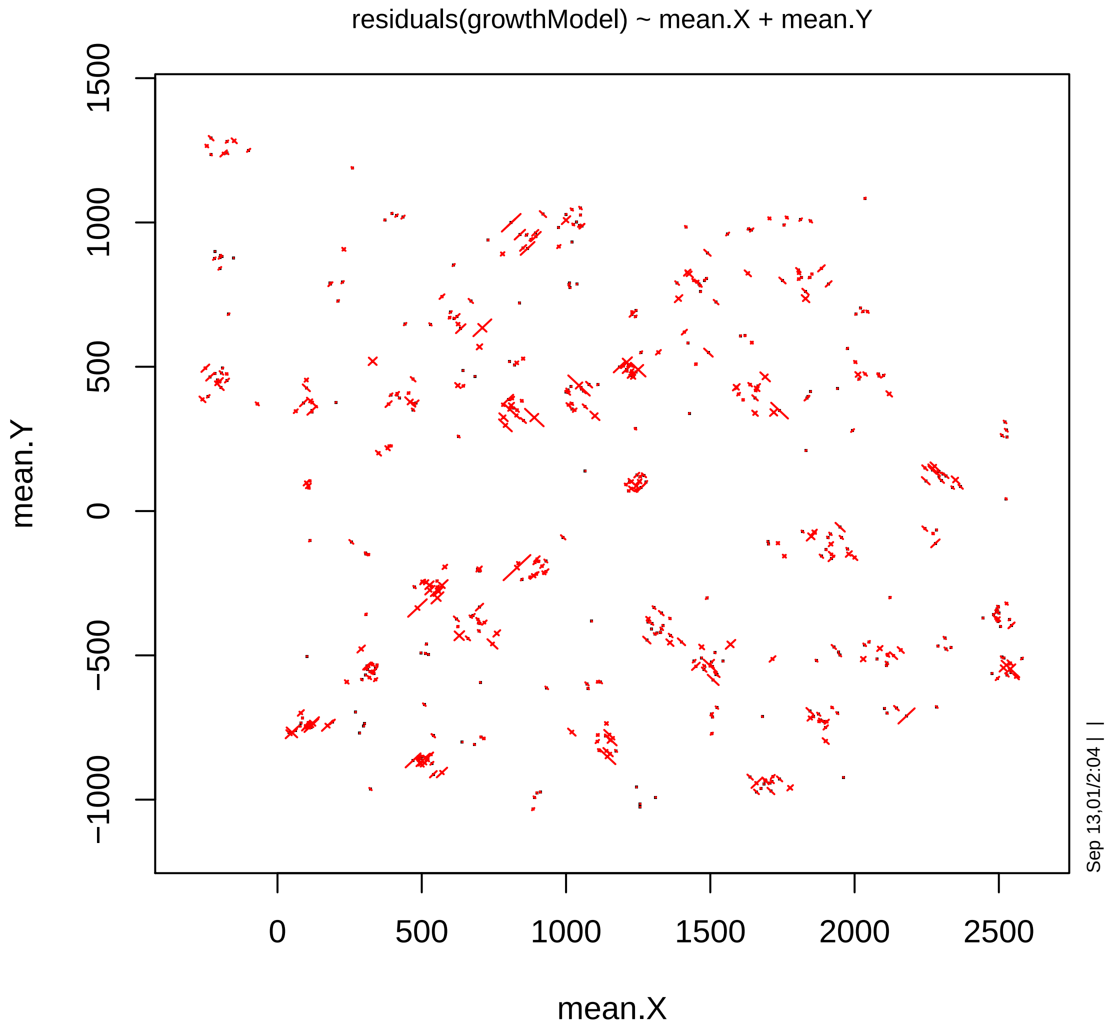


```
[1] "plres2x done"

plot(variog(data = residuals(growthModel), coords = growth[, c("mean.X", "mean.Y")],
    max.dist = 500), pch = 20, main = "Spatial correlation of growth model residuals")

variog: computing omnidirectional variogram
variog: co-locatted data found, adding one bin at the origin
```

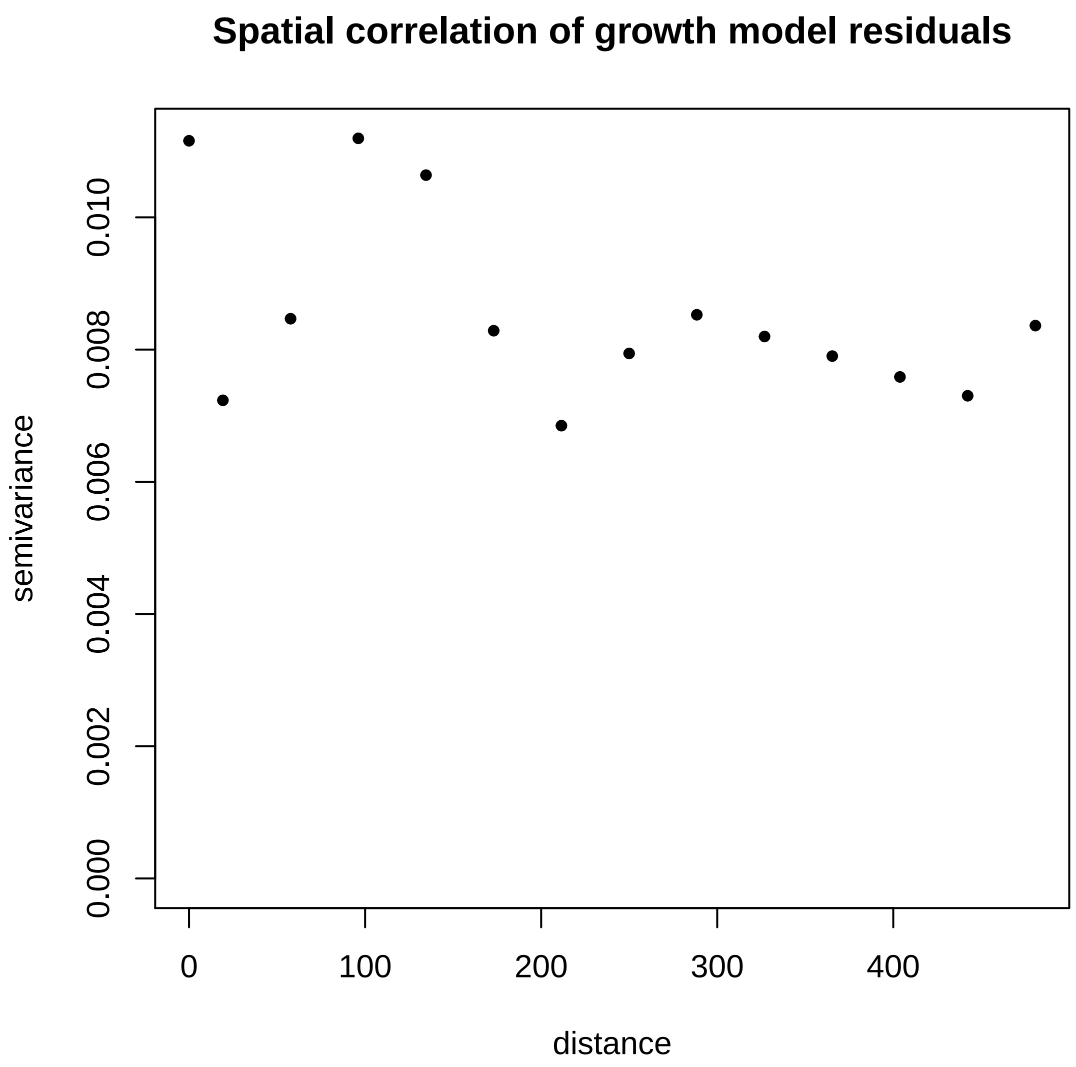

5.5 Models inference

Inference not necessary for our results as we're not testing hypotheses, but we'll do some anyway to make sure there are no problems with random effects and so on.

```
survivalModel.ML <- update(survivalModel, REML = FALSE)
drop1(survivalModel.ML, test = "Chi")

Single term deletions

Model:
survival ~ spBySurveyByComp + (1 | sp.pl)
                  Df   AIC  LRT Pr(Chi)
<none>                -2418
spBySurveyByComp 191  -763 2037  <2e-16 ***
---
Signif. codes:  0 `***' 0.001 `**' 0.01 `*' 0.05 `.' 0.1 ` ' 1

growthModel.ML <- update(growthModel, REML = FALSE)
```

```
drop1(growthModel.ML, test = "Chi")

Single term deletions

Model:
mean.log.diam ~ SP.sp.comp * mdsp.sc + (1 | sp.pl)
                   Df   AIC LRT Pr(Chi)
<none>                 -977
SP.sp.comp:mdsp.sc 95 -999 167   7e-06 ***
---
Signif. codes:  0 `***' 0.001 `**' 0.01 `*' 0.05 `.' 0.1 ` ' 1
```

Let's do profile zeta plots for survival:

```
survivalProfiles <- profile(survivalModel, which = "theta_")

confint(survivalProfiles)

       2.5 % 97.5 %
.sig01 0.064  0.075
.sigma 0.059  0.065

xyplot(survivalProfiles, level = c(0.95, 0.99))
```

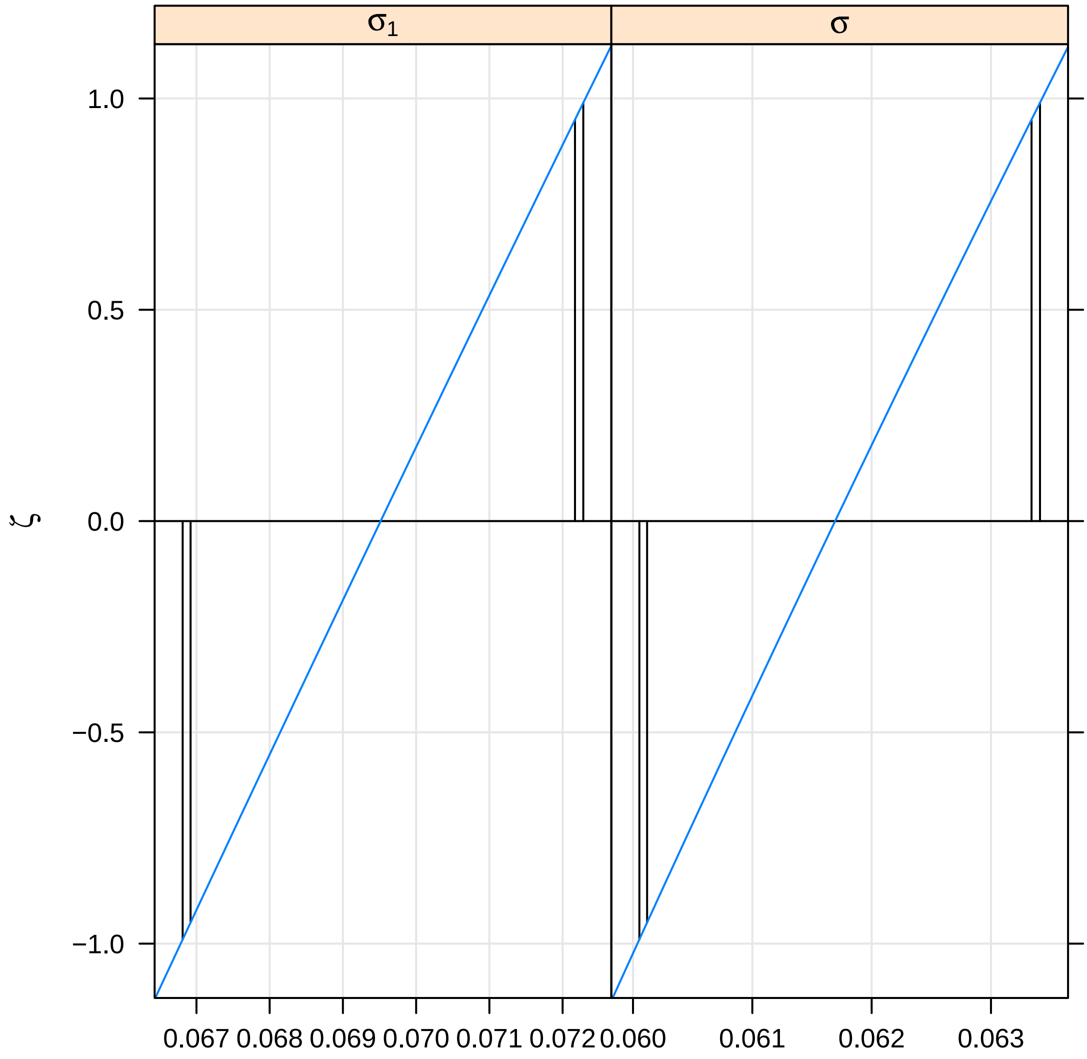

And zeta plots for growth:

```
growthProfiles <- profile(growthModel, which = "theta_")

confint(growthProfiles)

       2.5 % 97.5 %
.sig01 0.079  0.098
.sigma 0.100  0.112

xyplot(growthProfiles, level = c(0.95, 0.99))
```

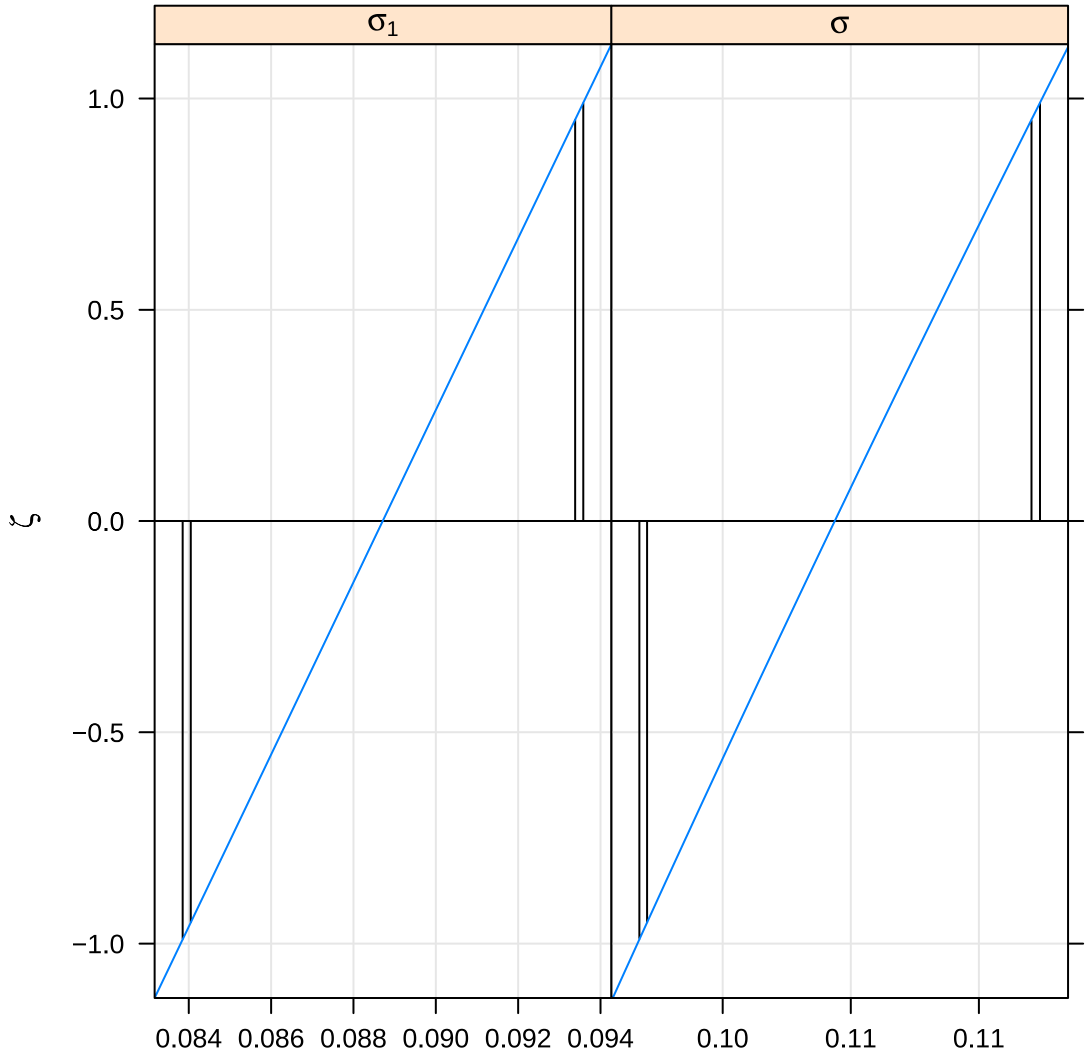

The plot looks linear, so the normality assumptions on the distribution of the variance component seem to be sensible (see lme4 manual).

```
splom(survivalProfiles)
```

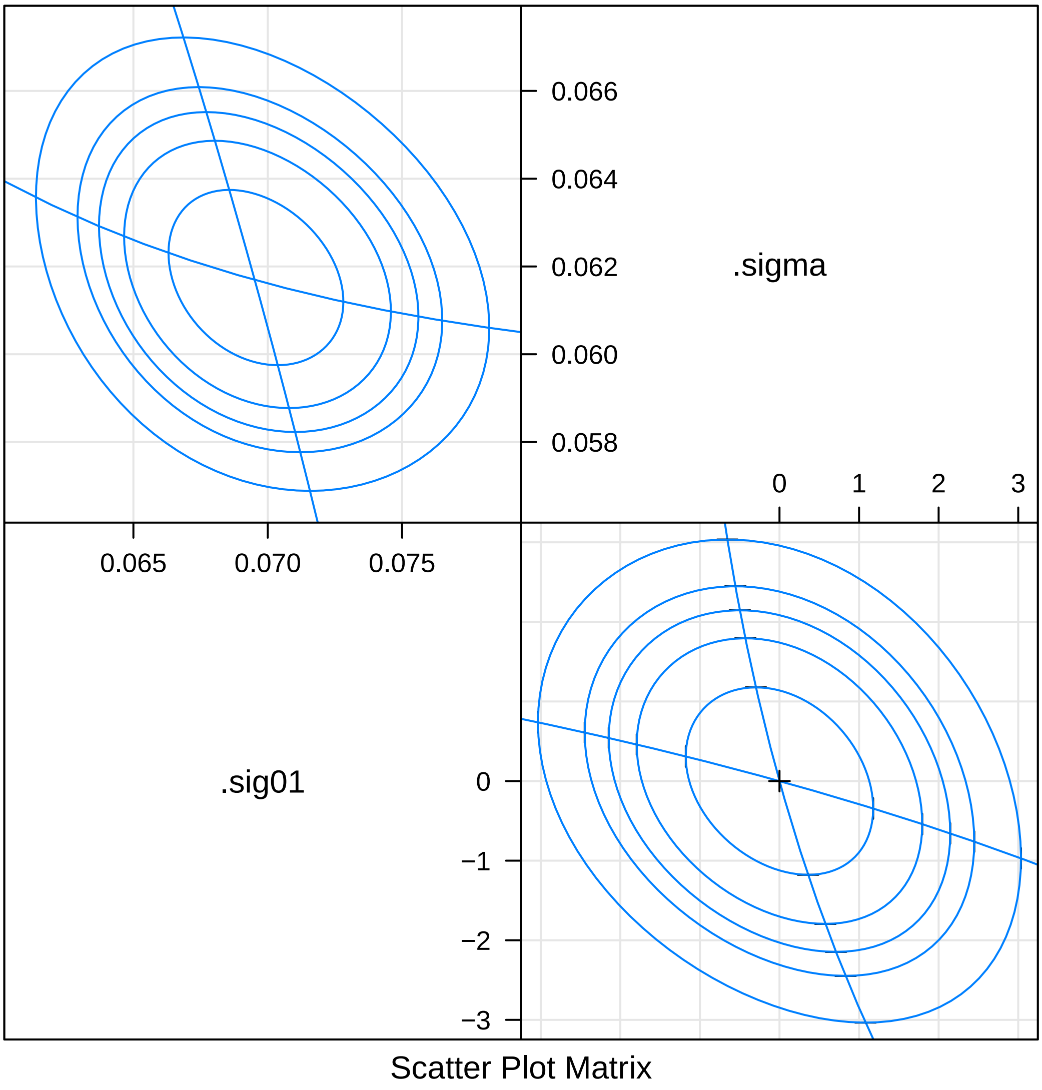


```
splom(growthProfiles)
```

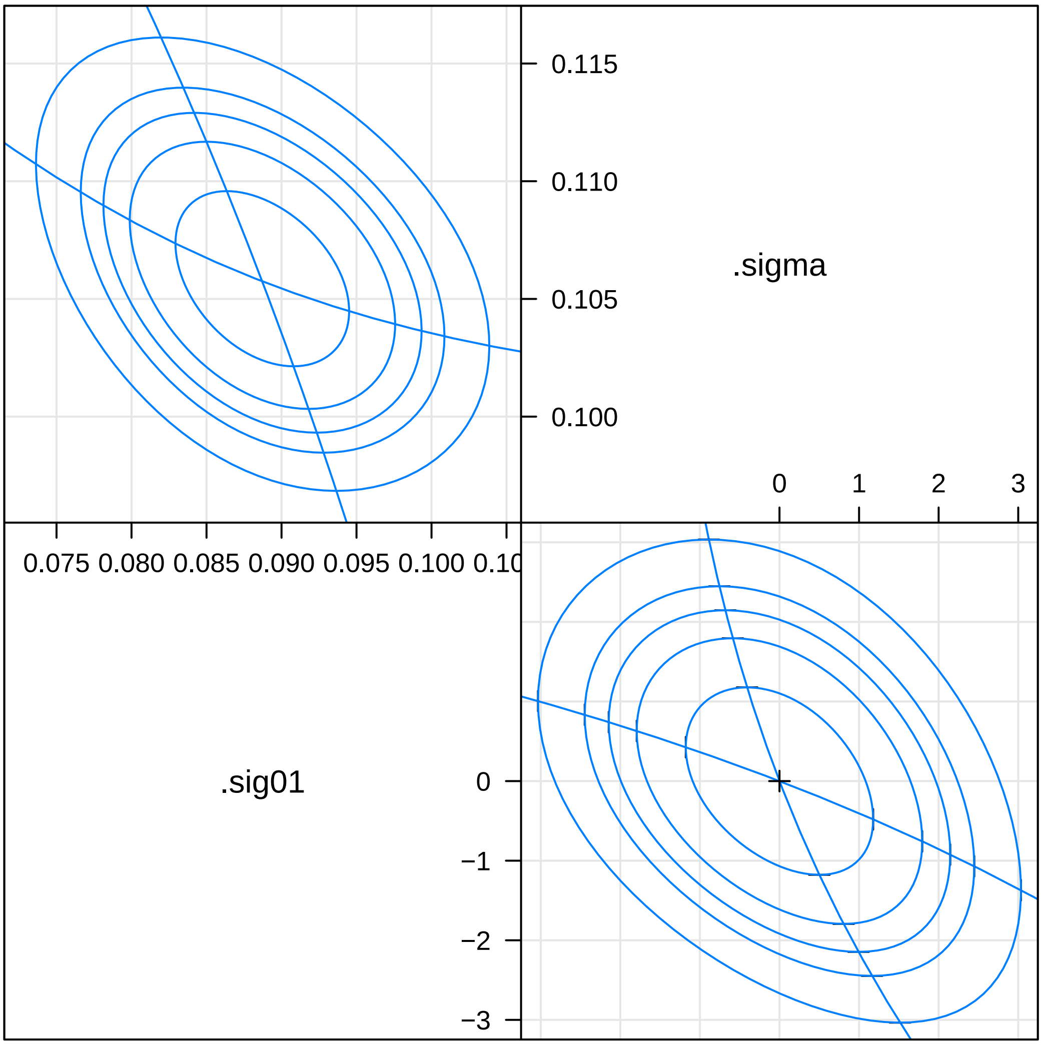

Scatter Plot Matrix

There is no sign of correlation between variance components in either model.

6 Extract information from models

6.1 Extract growth slope values

```
showd(names(fixef(growthModel)))


                                        r.1
data (Intercept)
                                        r.2
data SP.sp.compleprosula.4.1
                                        r.3
data SP.sp.compmalaanonan.4.1
                                        ...
data
                                       r.50
data SP.sp.compgibbosa.4.6
```

```
                                        r.98
data SP.sp.compleprosula.4.1:mdsp.sc
                                       r.145
data SP.sp.compferruginea.4.6:mdsp.sc
                                       r.192
data SP.sp.comptomentella.tomentella:mdsp.sc

levels(growth$sp)

 [1] "argentifolia" "beccariana"   "conformis"    "faguetiana"
 [5] "ferruginea"   "gibbosa"      "johorensis"   "lanceolata"
 [9] "leprosula"    "macrophylla"  "macroptera"   "malaanonan"
[13] "ovalis"       "parvifolia"   "sangal"       "tomentella"

levels(growth$sp.comp)

 [1] "4.1"          "4.10"         "4.11"         "4.12"
 [5] "4.13"         "4.14"         "4.15"         "4.16"
 [9] "4.2"          "4.3"          "4.4"          "4.5"
[13] "4.6"          "4.7"          "4.8"          "4.9"
[17] "argentifolia" "beccariana"   "conformis"    "faguetiana"
[21] "ferruginea"   "gibbosa"      "johorensis"   "lanceolata"
[25] "leprosula"    "macrophylla"  "macroptera"   "malaanonan"
[29] "ovalis"       "parvifolia"   "sangal"       "sixteen"
[33] "tomentella"

## argentifolia.4.1 does not exist, therefore is droped and the first level
## is beccariana.4.1

levels(growth$SP.sp.comp)[1]

[1] "beccariana.4.1"

fixef(growthModel)[1] ## is actually beccariana.4.1.

(Intercept)
          1

## this should be added to all other intercepts (treatment contrasts)

t.int <- grep(pattern = "mdsp.sc", x = names(fixef(growthModel)))

growthCoefs <- data.frame(slopes = unname(fixef(growthModel))[t.int],
                          #comb.2=names(fixef(mod.6)[t.int]),
                          intercepts = unname(fixef(growthModel))[-t.int],
                          comb = names(fixef(growthModel)[-t.int]),
                          stringsAsFactors = FALSE)
head(growthCoefs)
```

```
   slopes intercepts                      comb
1  0.2147      1.040               (Intercept)
2 -0.0586     -0.011   SP.sp.compleprosula.4.1
3 -0.0883     -0.034  SP.sp.compmalaanonan.4.1
4 -0.0560     -0.026  SP.sp.comptomentella.4.1
5 -0.0526     -0.116  SP.sp.compconformis.4.10
6 -0.0066     -0.035 SP.sp.compjohorensis.4.10

str(growthCoefs)

'data.frame': 96 obs. of  3 variables:
 $ slopes    : num  0.2147 -0.0586 -0.0883 -0.056 -0.0526 ...
 $ intercepts: num  1.0396 -0.0108 -0.0338 -0.0262 -0.1157 ...
 $ comb      : chr  "(Intercept)" "SP.sp.compleprosula.4.1" "SP.sp.compmalaanonan.4.1" "SP.sp.compt

growthCoefs$intercepts <- growthCoefs$intercepts +
  c(0, rep(growthCoefs$intercepts[1], times = nrow(growthCoefs)-1))
growthCoefs$slopes <- growthCoefs$slopes +
  c(0, rep(growthCoefs$slopes[1], times = nrow(growthCoefs)-1))

growthCoefs$comb[1] <- paste0("SP.sp.comp", levels(growth$SP.sp.comp)[1])
growthCoefs$comb <- str_sub(string = growthCoefs$comb, start = 11, end= -1)

growthCoefs$sp <- sub(pattern = "\\..*", replacement = "",
                      x = growthCoefs$comb)
growthCoefs$sp.comp <- sub(pattern = "^.*?\\.", replacement = "",
                           x = growthCoefs$comb)

showd(growthCoefs)

dim:  96 5
    slopes intercepts                  comb         sp    sp.comp
1     0.21        1.0        beccariana.4.1 beccariana        4.1
2     0.16        1.0         leprosula.4.1  leprosula        4.1
3     0.13        1.0        malaanonan.4.1 malaanonan        4.1
...
26    0.23        1.0       lanceolata.4.15 lanceolata       4.15
50    0.24        1.0           gibbosa.4.6    gibbosa        4.6
73    0.21        1.1   leprosula.leprosula  leprosula  leprosula
96    0.15        1.0 tomentella.tomentella tomentella tomentella
```

6.2 Extract survival fixed effects

Create data.frame of the fixed effects, then aggregate over species compositions to get mean species survival in each survey.

```
coefs <- data.frame(
  names = I(names(fixef(survivalModel))),
  survival = c(fixef(survivalModel)[1],
             fixef(survivalModel)[1]+fixef(survivalModel)[2:192]),
```

```
  row.names = NULL)

coefs$names[1] <- "spBySurveyByCompbeccariana.census1.4.1"

coefs$names <- substr(coefs$names, 17, nchar(coefs$names))

coefs$species <- unname(sapply(coefs$names,
                   function(x) substr(x, 1, gregexpr("\\.", x)[[1]][1]-1)
                  ))
coefs$survey <- unname(sapply(coefs$names,
                  function(x) substr(x, gregexpr("\\.", x)[[1]][1]+1,
                                   gregexpr("\\.", x)[[1]][2]-1)
                 ))
coefs$comp <- unname(sapply(coefs$names,
                function(x) substr(x, gregexpr("\\.", x)[[1]][2]+1, nchar(x))
              ))

coefs$richness[coefs$comp == "sixteen"] <- 16
coefs$richness[grepl("4", coefs$comp)] <- 4
coefs$richness[is.na(coefs$richness)] <- 1
coefs$richness <- factor(coefs$richness)

all.equal(growthCoefs$sp, coefs$species[coefs$survey == "census1"])

[1] TRUE

all.equal(growthCoefs$sp, coefs$species[coefs$survey == "census2"])

[1] TRUE

all.equal(growthCoefs$comp, coefs$sp.comp[coefs$survey == "census1"])

[1] TRUE

all.equal(growthCoefs$comp, coefs$sp.comp[coefs$survey == "census2"])

[1] TRUE

coefs$growthSlopes[coefs$survey == "census1"] <- growthCoefs$slopes
coefs$growthSlopes[coefs$survey == "census2"] <- growthCoefs$slopes
coefs$growthIntercepts[coefs$survey == "census1"] <- growthCoefs$intercepts
coefs$growthIntercepts[coefs$survey == "census2"] <- growthCoefs$intercepts

## Make species names in coefs consistent
coefs$species <- as.character(coefs$species)
coefs$species[coefs$species == "argentifolia"] <- "S. argentifolia"
coefs$species[coefs$species == "beccariana"] <- "S. beccariana"
coefs$species[coefs$species == "conformis"] <- "D. conformis"
coefs$species[coefs$species == "faguetiana"] <- "S. faguetiana"
coefs$species[coefs$species == "ferruginea"] <- "H. ferruginea"
```

```
coefs$species[coefs$species == "gibbosa"] <- "S. gibbosa"
coefs$species[coefs$species == "johorensis"] <- "S. johorensis"
coefs$species[coefs$species == "lanceolata"] <- "D. lanceolata"
coefs$species[coefs$species == "leprosula"] <- "S. leprosula"
coefs$species[coefs$species == "macrophylla"] <- "S. macrophylla"
coefs$species[coefs$species == "macroptera"] <- "S. macroptera"
coefs$species[coefs$species == "malaanonan"] <- "P. malaanonan"
coefs$species[coefs$species == "ovalis"] <- "S. ovalis"
coefs$species[coefs$species == "parvifolia"] <- "S. parvifolia"
coefs$species[coefs$species == "sangal"] <- "H. sangal"
coefs$species[coefs$species == "tomentella"] <- "P. tomentella"
```

6.3 Collate information into ideal data.frame for plots

Get species averages of survival, averaged over species composition treatments, by taking the model (population-level) predictions and aggregating by species and survey. Doing it this way deals with the fact that some species composition treatment estimates are based on a greater number of plots. This way of calculating species averages describes each species overall performance across the experiment (speciesPerformance data.frame). Another way would be to just take the average of species composition model estimates, for each species (see speciesMeans data.frame): this would describe the average effect of species composition treatments on each species. The first average is desirable for manuscript figure 2, the second is better for figure 3.

```
survival$modelPreds <- predict(survivalModel, re.form = ~ 0)

speciesPerformance <- aggregate(modelPreds ~ sp + survey.type, data = survival,
                                FUN = mean)
speciesPerformance$sp <- as.character(speciesPerformance$sp)

## Make species names in coefs consistent
findSp <- function(name, data = speciesPerformance) data$sp == name
speciesPerformance$sp <- as.character(speciesPerformance$sp)
speciesPerformance$sp[findSp("argentifolia")] <- "S. argentifolia"
speciesPerformance$sp[findSp("beccariana")] <- "S. beccariana"
speciesPerformance$sp[findSp("conformis")] <- "D. conformis"
speciesPerformance$sp[findSp("faguetiana")] <- "S. faguetiana"
speciesPerformance$sp[findSp("ferruginea")] <- "H. ferruginea"
speciesPerformance$sp[findSp("gibbosa")] <- "S. gibbosa"
speciesPerformance$sp[findSp("johorensis")] <- "S. johorensis"
speciesPerformance$sp[findSp("lanceolata")] <- "D. lanceolata"
speciesPerformance$sp[findSp("leprosula")] <- "S. leprosula"
speciesPerformance$sp[findSp("macrophylla")] <- "S. macrophylla"
speciesPerformance$sp[findSp("macroptera")] <- "S. macroptera"
speciesPerformance$sp[findSp("malaanonan")] <- "P. malaanonan"
speciesPerformance$sp[findSp("ovalis")] <- "S. ovalis"
speciesPerformance$sp[findSp("parvifolia")] <- "S. parvifolia"
speciesPerformance$sp[findSp("sangal")] <- "H. sangal"
speciesPerformance$sp[findSp("tomentella")] <- "P. tomentella"

speciesPerformance$codes[findSp("S. argentifolia")] <- "SA"
speciesPerformance$codes[findSp("S. beccariana")] <- "SB"
```

```
speciesPerformance$codes[findSp("D. conformis")] <- "DC"
speciesPerformance$codes[findSp("S. faguetiana")] <- "SF"
speciesPerformance$codes[findSp("H. ferruginea")] <- "HF"
speciesPerformance$codes[findSp("S. gibbosa")] <- "SG"
speciesPerformance$codes[findSp("S. johorensis")] <- "SJ"
speciesPerformance$codes[findSp("D. lanceolata")] <- "DL"
speciesPerformance$codes[findSp("S. leprosula")] <- "SL"
speciesPerformance$codes[findSp("S. macrophylla")] <- "SM1"
speciesPerformance$codes[findSp("S. macroptera")] <- "SM2"
speciesPerformance$codes[findSp("P. malaanonan")] <- "PM"
speciesPerformance$codes[findSp("S. ovalis")] <- "SO"
speciesPerformance$codes[findSp("S. parvifolia")] <- "SP"
speciesPerformance$codes[findSp("H. sangal")] <- "HS"
speciesPerformance$codes[findSp("P. tomentella")] <- "PT"

speciesPerformance$genus[findSp("S. argentifolia")] <- "Shorea"
speciesPerformance$genus[findSp("S. beccariana")] <- "Shorea"
speciesPerformance$genus[findSp("D. conformis")] <- "Dipterocarpus"
speciesPerformance$genus[findSp("S. faguetiana")] <- "Shorea"
speciesPerformance$genus[findSp("H. ferruginea")] <- "Hopea"
speciesPerformance$genus[findSp("S. gibbosa")] <- "Shorea"
speciesPerformance$genus[findSp("S. johorensis")] <- "Shorea"
speciesPerformance$genus[findSp("D. lanceolata")] <- "Dryobalanops"
speciesPerformance$genus[findSp("S. leprosula")] <- "Shorea"
speciesPerformance$genus[findSp("S. macrophylla")] <- "Shorea"
speciesPerformance$genus[findSp("S. macroptera")] <- "Shorea"
speciesPerformance$genus[findSp("P. malaanonan")] <- "Parashorea"
speciesPerformance$genus[findSp("S. ovalis")] <- "Shorea"
speciesPerformance$genus[findSp("S. parvifolia")] <- "Shorea"
speciesPerformance$genus[findSp("H. sangal")] <- "Hopea"
speciesPerformance$genus[findSp("P. tomentella")] <- "Parashorea"

speciesPerformance$section[findSp("S. argentifolia")] <- "Mutica"
speciesPerformance$section[findSp("S. beccariana")] <- "Pachycarpae"
speciesPerformance$section[findSp("D. conformis")] <- "NotShorea"
speciesPerformance$section[findSp("S. faguetiana")] <- "Richetioides"
speciesPerformance$section[findSp("H. ferruginea")] <- "NotShorea"
speciesPerformance$section[findSp("S. gibbosa")] <- "Richetioides"
speciesPerformance$section[findSp("S. johorensis")] <- "Brachypterae"
speciesPerformance$section[findSp("D. lanceolata")] <- "NotShorea"
speciesPerformance$section[findSp("S. leprosula")] <- "Mutica"
speciesPerformance$section[findSp("S. macrophylla")] <- "Pachycarpae"
speciesPerformance$section[findSp("S. macroptera")] <- "Auriculatae"
speciesPerformance$section[findSp("P. malaanonan")] <- "NotShorea"
speciesPerformance$section[findSp("S. ovalis")] <- "Ovalis"
speciesPerformance$section[findSp("S. parvifolia")] <- "Mutica"
speciesPerformance$section[findSp("H. sangal")] <- "NotShorea"
speciesPerformance$section[findSp("P. tomentella")] <- "NotShorea"

findGenus <- function(name, data = speciesPerformance) data$genus == name
speciesPerformance$colour[findGenus("Shorea")] <- "#8DA0CB"
speciesPerformance$colour[findGenus("Parashorea")] <- "#FC8D62"
```

```
speciesPerformance$colour[findGenus("Hopea")] <- "#66C2A5"
speciesPerformance$colour[findGenus("Dryobalanops")] <- "#E78AC3"
speciesPerformance$colour[findGenus("Dipterocarpus")] <- "#A6D854"

speciesPerformance
```

```
                sp survey.type modelPreds codes         genus      section
1  S. argentifolia     census1      0.193    SA        Shorea       Mutica
2    S. beccariana     census1      0.393    SB        Shorea  Pachycarpae
3     D. conformis     census1      0.431    DC Dipterocarpus    NotShorea
4    S. faguetiana     census1      0.219    SF        Shorea Richetioides
5    H. ferruginea     census1      0.196    HF         Hopea    NotShorea
6       S. gibbosa     census1      0.228    SG        Shorea Richetioides
7    S. johorensis     census1      0.435    SJ        Shorea Brachypterae
8    D. lanceolata     census1      0.399    DL  Dryobalanops    NotShorea
9     S. leprosula     census1      0.321    SL        Shorea       Mutica
10  S. macrophylla     census1      0.283   SM1        Shorea  Pachycarpae
11   S. macroptera     census1      0.368   SM2        Shorea  Auriculatae
12   P. malaanonan     census1      0.422    PM    Parashorea    NotShorea
13       S. ovalis     census1      0.497    SO        Shorea       Ovalis
14   S. parvifolia     census1      0.275    SP        Shorea       Mutica
15       H. sangal     census1      0.582    HS         Hopea    NotShorea
16   P. tomentella     census1      0.467    PT    Parashorea    NotShorea
17 S. argentifolia     census2      0.020    SA        Shorea       Mutica
18   S. beccariana     census2      0.140    SB        Shorea  Pachycarpae
19    D. conformis     census2      0.167    DC Dipterocarpus    NotShorea
20   S. faguetiana     census2      0.039    SF        Shorea Richetioides
21   H. ferruginea     census2      0.069    HF         Hopea    NotShorea
22      S. gibbosa     census2      0.061    SG        Shorea Richetioides
23   S. johorensis     census2      0.150    SJ        Shorea Brachypterae
24   D. lanceolata     census2      0.143    DL  Dryobalanops    NotShorea
25    S. leprosula     census2      0.059    SL        Shorea       Mutica
26  S. macrophylla     census2      0.068   SM1        Shorea  Pachycarpae
27   S. macroptera     census2      0.099   SM2        Shorea  Auriculatae
28   P. malaanonan     census2      0.159    PM    Parashorea    NotShorea
29       S. ovalis     census2      0.183    SO        Shorea       Ovalis
30   S. parvifolia     census2      0.048    SP        Shorea       Mutica
31       H. sangal     census2      0.270    HS         Hopea    NotShorea
32   P. tomentella     census2      0.183    PT    Parashorea    NotShorea
    colour
1  #8DA0CB
2  #8DA0CB
3  #A6D854
4  #8DA0CB
5  #66C2A5
6  #8DA0CB
7  #8DA0CB
8  #E78AC3
9  #8DA0CB
10 #8DA0CB
11 #8DA0CB
12 #FC8D62
```

```
13 #8DA0CB
14 #8DA0CB
15 #66C2A5
16 #FC8D62
17 #8DA0CB
18 #8DA0CB
19 #A6D854
20 #8DA0CB
21 #66C2A5
22 #8DA0CB
23 #8DA0CB
24 #E78AC3
25 #8DA0CB
26 #8DA0CB
27 #8DA0CB
28 #FC8D62
29 #8DA0CB
30 #8DA0CB
31 #66C2A5
32 #FC8D62
```

```
speciesMeans <- summaryBy(
  survival + growthSlopes + growthIntercepts ~ species + survey,
  data = coefs, FUN = mean, keep.names = TRUE)

speciesMeans$species <- as.character(speciesMeans$species)

speciesMeans$codes[speciesMeans$species == "S. argentifolia"] <- "SA"
speciesMeans$codes[speciesMeans$species == "S. beccariana"] <- "SB"
speciesMeans$codes[speciesMeans$species == "D. conformis"] <- "DC"
speciesMeans$codes[speciesMeans$species == "S. faguetiana"] <- "SF"
speciesMeans$codes[speciesMeans$species == "H. ferruginea"] <- "HF"
speciesMeans$codes[speciesMeans$species == "S. gibbosa"] <- "SG"
speciesMeans$codes[speciesMeans$species == "S. johorensis"] <- "SJ"
speciesMeans$codes[speciesMeans$species == "D. lanceolata"] <- "DL"
speciesMeans$codes[speciesMeans$species == "S. leprosula"] <- "SL"
speciesMeans$codes[speciesMeans$species == "S. macrophylla"] <- "SM1"
speciesMeans$codes[speciesMeans$species == "S. macroptera"] <- "SM2"
speciesMeans$codes[speciesMeans$species == "P. malaanonan"] <- "PM"
speciesMeans$codes[speciesMeans$species == "S. ovalis"] <- "SO"
speciesMeans$codes[speciesMeans$species == "S. parvifolia"] <- "SP"
speciesMeans$codes[speciesMeans$species == "H. sangal"] <- "HS"
speciesMeans$codes[speciesMeans$species == "P. tomentella"] <- "PT"

speciesMeans$genus[speciesMeans$species == "S. argentifolia"] <- "Shorea"
speciesMeans$genus[speciesMeans$species == "S. beccariana"] <- "Shorea"
speciesMeans$genus[speciesMeans$species == "D. conformis"] <- "Dipterocarpus"
speciesMeans$genus[speciesMeans$species == "S. faguetiana"] <- "Shorea"
speciesMeans$genus[speciesMeans$species == "H. ferruginea"] <- "Hopea"
speciesMeans$genus[speciesMeans$species == "S. gibbosa"] <- "Shorea"
speciesMeans$genus[speciesMeans$species == "S. johorensis"] <- "Shorea"
speciesMeans$genus[speciesMeans$species == "D. lanceolata"] <- "Dryobalanops"
```

```
speciesMeans$genus[speciesMeans$species == "S. leprosula"] <- "Shorea"
speciesMeans$genus[speciesMeans$species == "S. macrophylla"] <- "Shorea"
speciesMeans$genus[speciesMeans$species == "S. macroptera"] <- "Shorea"
speciesMeans$genus[speciesMeans$species == "P. malaanonan"] <- "Parashorea"
speciesMeans$genus[speciesMeans$species == "S. ovalis"] <- "Shorea"
speciesMeans$genus[speciesMeans$species == "S. parvifolia"] <- "Shorea"
speciesMeans$genus[speciesMeans$species == "H. sangal"] <- "Hopea"
speciesMeans$genus[speciesMeans$species == "P. tomentella"] <- "Parashorea"

speciesMeans$section[speciesMeans$species == "S. argentifolia"] <- "Mutica"
speciesMeans$section[speciesMeans$species == "S. beccariana"] <- "Pachycarpae"
speciesMeans$section[speciesMeans$species == "D. conformis"] <- "NotShorea"
speciesMeans$section[speciesMeans$species == "S. faguetiana"] <- "Richetioides"
speciesMeans$section[speciesMeans$species == "H. ferruginea"] <- "NotShorea"
speciesMeans$section[speciesMeans$species == "S. gibbosa"] <- "Richetioides"
speciesMeans$section[speciesMeans$species == "S. johorensis"] <- "Brachypterae"
speciesMeans$section[speciesMeans$species == "D. lanceolata"] <- "NotShorea"
speciesMeans$section[speciesMeans$species == "S. leprosula"] <- "Mutica"
speciesMeans$section[speciesMeans$species == "S. macrophylla"] <- "Pachycarpae"
speciesMeans$section[speciesMeans$species == "S. macroptera"] <- "Auriculatae"
speciesMeans$section[speciesMeans$species == "P. malaanonan"] <- "NotShorea"
speciesMeans$section[speciesMeans$species == "S. ovalis"] <- "Ovalis"
speciesMeans$section[speciesMeans$species == "S. parvifolia"] <- "Mutica"
speciesMeans$section[speciesMeans$species == "H. sangal"] <- "NotShorea"
speciesMeans$section[speciesMeans$species == "P. tomentella"] <- "NotShorea"

speciesMeans$colour[speciesMeans$genus == "Shorea"] <- "#8DA0CB"
speciesMeans$colour[speciesMeans$genus == "Parashorea"] <- "#FC8D62"
speciesMeans$colour[speciesMeans$genus == "Hopea"] <- "#66C2A5"
speciesMeans$colour[speciesMeans$genus == "Dryobalanops"] <- "#E78AC3"
speciesMeans$colour[speciesMeans$genus == "Dipterocarpus"] <- "#A6D854"

speciesMeans

           species  survey survival growthSlopes growthIntercepts codes
1     D. conformis census1    0.403         0.16             0.96    DC
2     D. conformis census2    0.157         0.16             0.96    DC
3    D. lanceolata census1    0.353         0.22             1.05    DL
4    D. lanceolata census2    0.121         0.22             1.05    DL
5    H. ferruginea census1    0.178         0.29             0.89    HF
6    H. ferruginea census2    0.067         0.29             0.89    HF
7        H. sangal census1    0.585         0.22             1.04    HS
8        H. sangal census2    0.291         0.22             1.04    HS
9    P. malaanonan census1    0.422         0.17             1.01    PM
10   P. malaanonan census2    0.165         0.17             1.01    PM
11   P. tomentella census1    0.458         0.16             1.01    PT
12   P. tomentella census2    0.169         0.16             1.01    PT
13 S. argentifolia census1    0.164         0.27             1.23    SA
14 S. argentifolia census2    0.015         0.27             1.23    SA
15   S. beccariana census1    0.431         0.25             1.07    SB
16   S. beccariana census2    0.159         0.25             1.07    SB
17   S. faguetiana census1    0.210         0.23             1.01    SF
```

```
18   S. faguetiana census2    0.042          0.23              1.01     SF
19      S. gibbosa census1    0.231          0.23              1.01     SG
20      S. gibbosa census2    0.050          0.23              1.01     SG
21   S. johorensis census1    0.400          0.21              1.05     SJ
22   S. johorensis census2    0.135          0.21              1.05     SJ
23    S. leprosula census1    0.310          0.22              1.11     SL
24    S. leprosula census2    0.061          0.22              1.11     SL
25  S. macrophylla census1    0.327          0.20              1.23    SM1
26  S. macrophylla census2    0.091          0.20              1.23    SM1
27   S. macroptera census1    0.372          0.20              1.03    SM2
28   S. macroptera census2    0.108          0.20              1.03    SM2
29       S. ovalis census1    0.505          0.20              1.09     SO
30       S. ovalis census2    0.191          0.20              1.09     SO
31   S. parvifolia census1    0.275          0.25              1.09     SP
32   S. parvifolia census2    0.052          0.25              1.09     SP
           genus      section  colour
1  Dipterocarpus    NotShorea #A6D854
2  Dipterocarpus    NotShorea #A6D854
3   Dryobalanops    NotShorea #E78AC3
4   Dryobalanops    NotShorea #E78AC3
5          Hopea    NotShorea #66C2A5
6          Hopea    NotShorea #66C2A5
7          Hopea    NotShorea #66C2A5
8          Hopea    NotShorea #66C2A5
9     Parashorea    NotShorea #FC8D62
10    Parashorea    NotShorea #FC8D62
11    Parashorea    NotShorea #FC8D62
12    Parashorea    NotShorea #FC8D62
13        Shorea       Mutica #8DA0CB
14        Shorea       Mutica #8DA0CB
15        Shorea  Pachycarpae #8DA0CB
16        Shorea  Pachycarpae #8DA0CB
17        Shorea Richetioides #8DA0CB
18        Shorea Richetioides #8DA0CB
19        Shorea Richetioides #8DA0CB
20        Shorea Richetioides #8DA0CB
21        Shorea Brachypterae #8DA0CB
22        Shorea Brachypterae #8DA0CB
23        Shorea       Mutica #8DA0CB
24        Shorea       Mutica #8DA0CB
25        Shorea  Pachycarpae #8DA0CB
26        Shorea  Pachycarpae #8DA0CB
27        Shorea  Auriculatae #8DA0CB
28        Shorea  Auriculatae #8DA0CB
29        Shorea       Ovalis #8DA0CB
30        Shorea       Ovalis #8DA0CB
31        Shorea       Mutica #8DA0CB
32        Shorea       Mutica #8DA0CB

## Match growth estimates from speciesMeans to rows in speciesPerformance
speciesPerformance$growthSlopes[speciesPerformance$sp == "S. argentifolia"] <-
  speciesMeans$growthSlopes[speciesMeans$species == "S. argentifolia"]
```

```
speciesPerformance$growthSlopes[speciesPerformance$sp == "S. beccariana"] <-
  speciesMeans$growthSlopes[speciesMeans$species == "S. beccariana"]
speciesPerformance$growthSlopes[speciesPerformance$sp == "D. conformis"] <-
  speciesMeans$growthSlopes[speciesMeans$species == "D. conformis"]
speciesPerformance$growthSlopes[speciesPerformance$sp == "S. faguetiana"] <-
  speciesMeans$growthSlopes[speciesMeans$species == "S. faguetiana"]
speciesPerformance$growthSlopes[speciesPerformance$sp == "H. ferruginea"] <-
  speciesMeans$growthSlopes[speciesMeans$species == "H. ferruginea"]
speciesPerformance$growthSlopes[speciesPerformance$sp == "S. gibbosa"] <-
  speciesMeans$growthSlopes[speciesMeans$species == "S. gibbosa"]
speciesPerformance$growthSlopes[speciesPerformance$sp == "S. johorensis"] <-
  speciesMeans$growthSlopes[speciesMeans$species == "S. johorensis"]
speciesPerformance$growthSlopes[speciesPerformance$sp == "D. lanceolata"] <-
  speciesMeans$growthSlopes[speciesMeans$species == "D. lanceolata"]
speciesPerformance$growthSlopes[speciesPerformance$sp == "S. leprosula"] <-
  speciesMeans$growthSlopes[speciesMeans$species == "S. leprosula"]
speciesPerformance$growthSlopes[speciesPerformance$sp == "S. macrophylla"] <-
  speciesMeans$growthSlopes[speciesMeans$species == "S. macrophylla"]
speciesPerformance$growthSlopes[speciesPerformance$sp == "S. macroptera"] <-
  speciesMeans$growthSlopes[speciesMeans$species == "S. macroptera"]
speciesPerformance$growthSlopes[speciesPerformance$sp == "P. malaanonan"] <-
  speciesMeans$growthSlopes[speciesMeans$species == "P. malaanonan"]
speciesPerformance$growthSlopes[speciesPerformance$sp == "S. ovalis"] <-
  speciesMeans$growthSlopes[speciesMeans$species == "S. ovalis"]
speciesPerformance$growthSlopes[speciesPerformance$sp == "S. parvifolia"] <-
  speciesMeans$growthSlopes[speciesMeans$species == "S. parvifolia"]
speciesPerformance$growthSlopes[speciesPerformance$sp == "H. sangal"] <-
  speciesMeans$growthSlopes[speciesMeans$species == "H. sangal"]
speciesPerformance$growthSlopes[speciesPerformance$sp == "P. tomentella"] <-
  speciesMeans$growthSlopes[speciesMeans$species == "P. tomentella"]

speciesPerformance$growthIntercepts[speciesPerformance$sp == "S. argentifolia"] <-
  speciesMeans$growthIntercepts[speciesMeans$species == "S. argentifolia"]
speciesPerformance$growthIntercepts[speciesPerformance$sp == "S. beccariana"] <-
  speciesMeans$growthIntercepts[speciesMeans$species == "S. beccariana"]
speciesPerformance$growthIntercepts[speciesPerformance$sp == "D. conformis"] <-
  speciesMeans$growthIntercepts[speciesMeans$species == "D. conformis"]
speciesPerformance$growthIntercepts[speciesPerformance$sp == "S. faguetiana"] <-
  speciesMeans$growthIntercepts[speciesMeans$species == "S. faguetiana"]
speciesPerformance$growthIntercepts[speciesPerformance$sp == "H. ferruginea"] <-
  speciesMeans$growthIntercepts[speciesMeans$species == "H. ferruginea"]
speciesPerformance$growthIntercepts[speciesPerformance$sp == "S. gibbosa"] <-
  speciesMeans$growthIntercepts[speciesMeans$species == "S. gibbosa"]
speciesPerformance$growthIntercepts[speciesPerformance$sp == "S. johorensis"] <-
  speciesMeans$growthIntercepts[speciesMeans$species == "S. johorensis"]
speciesPerformance$growthIntercepts[speciesPerformance$sp == "D. lanceolata"] <-
  speciesMeans$growthIntercepts[speciesMeans$species == "D. lanceolata"]
speciesPerformance$growthIntercepts[speciesPerformance$sp == "S. leprosula"] <-
  speciesMeans$growthIntercepts[speciesMeans$species == "S. leprosula"]
speciesPerformance$growthIntercepts[speciesPerformance$sp == "S. macrophylla"] <-
  speciesMeans$growthIntercepts[speciesMeans$species == "S. macrophylla"]
speciesPerformance$growthIntercepts[speciesPerformance$sp == "S. macroptera"] <-
```

```r
  speciesMeans$growthIntercepts[speciesMeans$species == "S. macroptera"]
speciesPerformance$growthIntercepts[speciesPerformance$sp == "P. malaanonan"] <-
  speciesMeans$growthIntercepts[speciesMeans$species == "P. malaanonan"]
speciesPerformance$growthIntercepts[speciesPerformance$sp == "S. ovalis"] <-
  speciesMeans$growthIntercepts[speciesMeans$species == "S. ovalis"]
speciesPerformance$growthIntercepts[speciesPerformance$sp == "S. parvifolia"] <-
  speciesMeans$growthIntercepts[speciesMeans$species == "S. parvifolia"]
speciesPerformance$growthIntercepts[speciesPerformance$sp == "H. sangal"] <-
  speciesMeans$growthIntercepts[speciesMeans$species == "H. sangal"]
speciesPerformance$growthIntercepts[speciesPerformance$sp == "P. tomentella"] <-
  speciesMeans$growthIntercepts[speciesMeans$species == "P. tomentella"]

speciesPerformance$survey.type <- as.character(speciesPerformance$survey.type)
speciesPerformance$survey.type[speciesPerformance$survey.type == "census1"] <-
  "Survey 1"
speciesPerformance$survey.type[speciesPerformance$survey.type == "census2"] <-
  "Survey 2"

## Labelling placements variables to manually move species code names
## in figure 2.
speciesPerformance$labelX <- speciesPerformance$growthSlopes
speciesPerformance$labelX[speciesPerformance$sp == "S. gibbosa" &
                            speciesPerformance$survey.type == "Survey 1"] <-
  speciesPerformance$labelX[speciesPerformance$sp == "S. gibbosa" &
                              speciesPerformance$survey.type == "Survey 1"]-0.0015

speciesPerformance$labelX[speciesPerformance$sp == "S. faguetiana" &
                            speciesPerformance$survey.type == "Survey 1"] <-
  speciesPerformance$labelX[speciesPerformance$sp == "S. faguetiana" &
                              speciesPerformance$survey.type == "Survey 1"]+0.0035

speciesPerformance$labelX[speciesPerformance$sp == "D. conformis" &
                            speciesPerformance$survey.type == "Survey 2"] <-
  speciesPerformance$labelX[speciesPerformance$sp == "D. conformis" &
                              speciesPerformance$survey.type == "Survey 2"]-0.005

speciesPerformance$labelX[speciesPerformance$sp == "S. macrophylla" &
                            speciesPerformance$survey.type == "Survey 2"] <-
  speciesPerformance$labelX[speciesPerformance$sp == "S. macrophylla" &
                              speciesPerformance$survey.type == "Survey 2"]-0.007

speciesPerformance$labelX[speciesPerformance$sp == "S. faguetiana" &
                            speciesPerformance$survey.type == "Survey 2"] <-
  speciesPerformance$labelX[speciesPerformance$sp == "S. faguetiana" &
                              speciesPerformance$survey.type == "Survey 2"]+0.003

speciesPerformance$labelX[speciesPerformance$sp == "S. leprosula" &
                            speciesPerformance$survey.type == "Survey 2"] <-
  speciesPerformance$labelX[speciesPerformance$sp == "S. leprosula" &
                              speciesPerformance$survey.type == "Survey 2"]-0.001
```

Survival is currently cumulative so that survey shows 0-2 years and survey 2 shows 0-10 years survival.

Create another variable where survival is split (survey 1 = 0-2 years, survey 2 = 2-10 years). Then we can more clearly see if there are any changes between surveys

```
speciesPerformance$surveySurvivals <- NA
speciesPerformance$surveySurvivals[speciesPerformance$survey.type == "Survey 1"] <- speciesPerfo
    "Survey 1"]
speciesPerformance$surveySurvivals[speciesPerformance$survey.type == "Survey 2"] <- speciesPerfo
    "Survey 2"]/speciesPerformance$modelPreds[speciesPerformance$survey.type ==
    "Survey 1"]
```

7 Further plotting

7.1 Correlation between growth slopes and intercepts

There is mostly a strong positive correlation between intercepts and slopes, as we suspected from exploratory plots.

```
xyplot(intercepts ~ slopes, groups = sp, data = growthCoefs,
       type = c("p","r")) #|sp, scales="free")
```

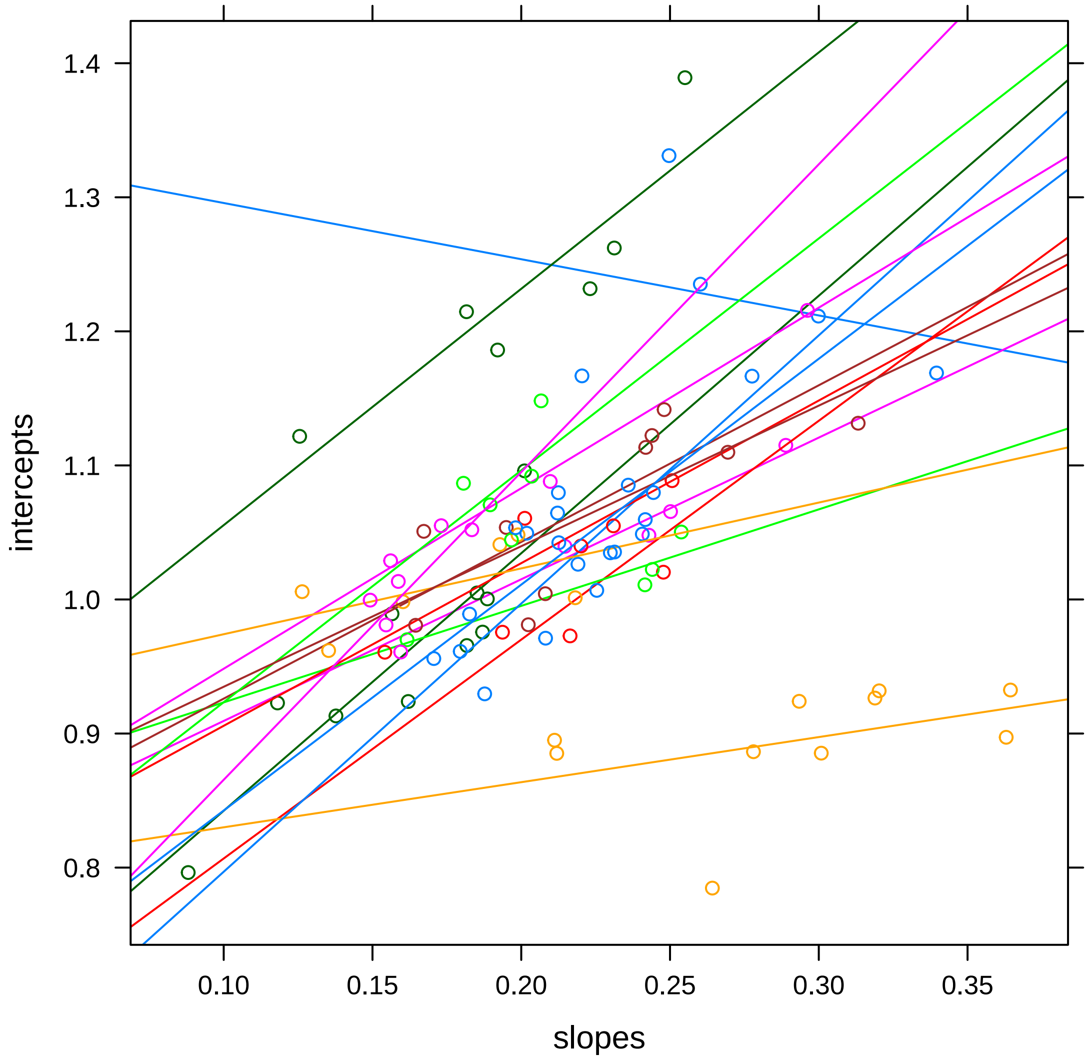

7.2 Species responses to composition treatments

Show each species response to composition treatments, in terms of growth and survival. Join species composition mean survival by points and lines within each species, and colour by species richness. Then show the average composition effect; producing 16 groups of points and lines, in survival vs growth space.

```r
ggplot(subset(coefs, survey == "census1"),
       aes(x = growthSlopes, y = survival, group = species,
           colour = richness)) +
  geom_point(size = 3) + geom_line(size = 1) + theme_bw() +
  labs(x = "Growth",
       y = "Proportion survival after 10 years",
       title = paste("Species growth and survival responses",
                     "to composition treatments"))
```

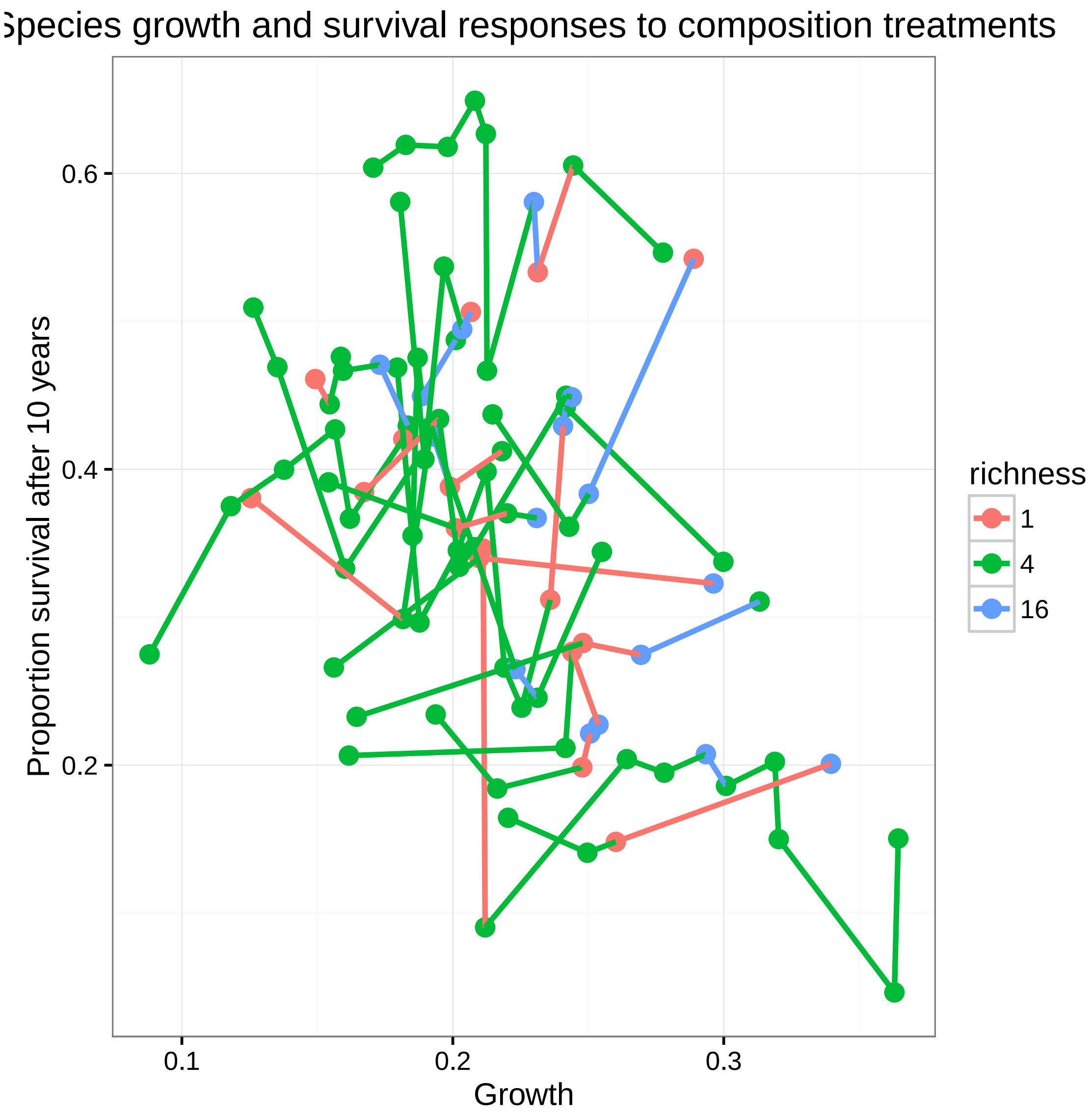

Survival shows no consistent trend with increasing species richness, in either survey. The plot level means, coloured by species richness treatment, show random scatter.

```r
qplot(data = coefs, x = survival, y = species, colour = richness, geom = "point")
```

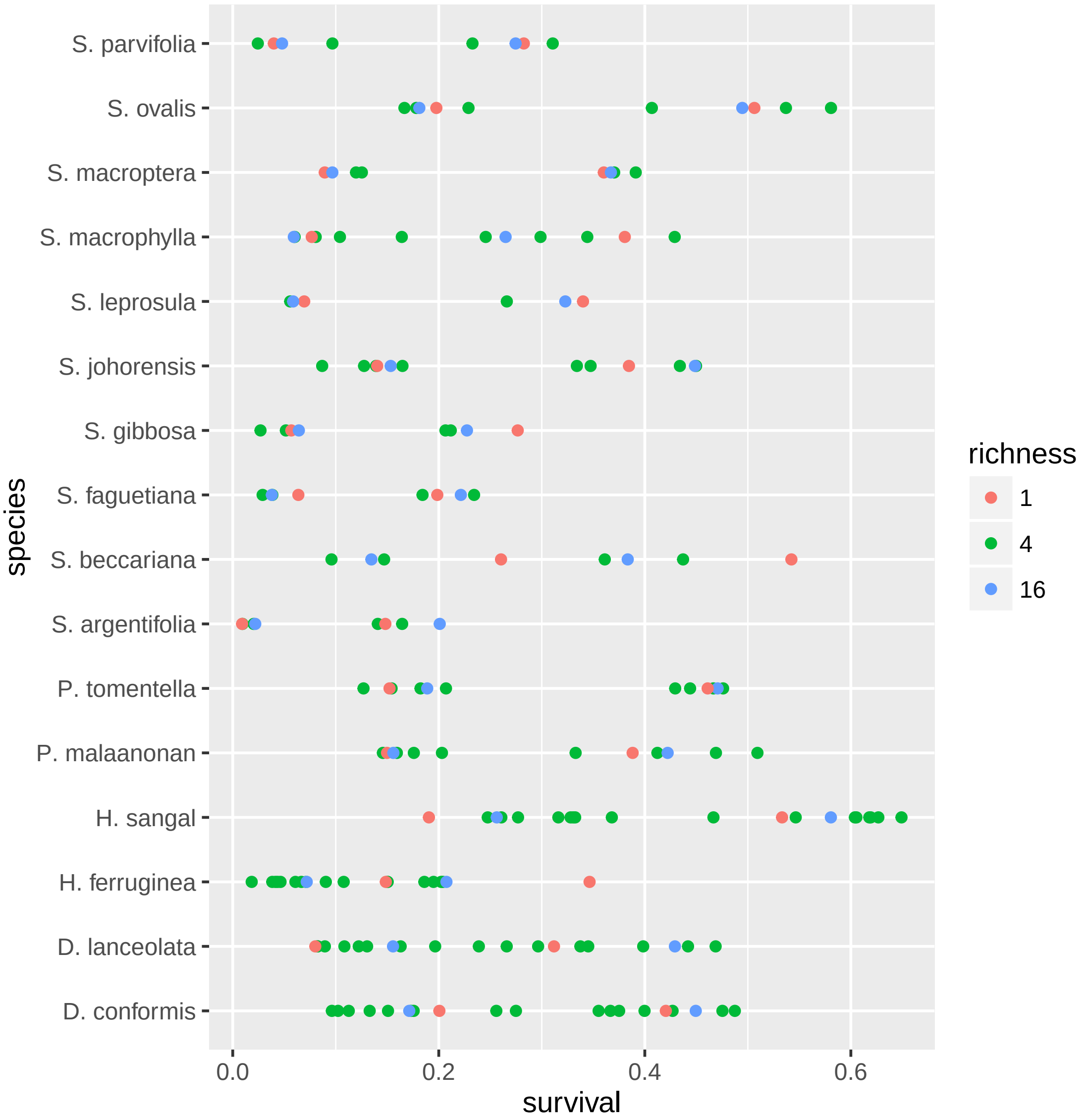

S. parvifolia
S. ovalis
S. macroptera
S. macrophylla
S. leprosula
S. johorensis
S. gibbosa
S. faguetiana
S. beccariana
S. argentifolia
P. tomentella
P. malaanonan
H. sangal
H. ferruginea
D. lanceolata
D. conformis
species
0.0
0.2
0.4
0.6
survival
richness
1
4
16

8 Manuscript figures

8.1 Figure 2

Figure 1 is a map of the experiment.

Show species averages of mortality (both surveys) vs growth. The plot will present the mortality and growth estimates for each species, show the changes in mortality between surveys and whether this relates to the magnitude of initial mortality or to growth performance, and whether mortality and growth seem correlated. Species is added on the x-axis in the absence of growth results.

Figure 2a: relationship between survival and growth estimates, showing survival after survey 1 (2 years) and survey 2 (10 years).

```
fig2a <- ggplot(speciesPerformance, aes(x = growthSlopes, y = modelPreds,
       colour = genus, label = codes)) + theme_bw() +
    geom_smooth(aes(group = 1), colour = "gray90",
              method = "lm", se = FALSE, size = 2) +
  geom_point(size = 4) + facet_grid(.~survey.type) +
  geom_text(aes(x = labelX, y = modelPreds+0.0225), show_guide = FALSE,
            fontface = "bold", size = 2.5) +
  labs(y = "Proportion survival", x = "Growth (log scale)") +
  scale_y_continuous(breaks = seq(from = 0.0, to = 0.6, by = 0.1)) +
  scale_x_continuous(breaks = seq(from = 0.1, to = 0.3, by = 0.02)) +
  scale_colour_manual(values = with(speciesPerformance, c(
    Shorea = unique(colour[genus == "Shorea"]),
    Parashorea = unique(colour[genus == "Parashorea"]),
    Hopea = unique(colour[genus == "Hopea"]),
    Dryobalanops = unique(colour[genus == "Dryobalanops"]),
    Dipterocarpus = unique(colour[genus == "Dipterocarpus"])))) +
  theme(legend.position = "none")

Warning: `show_guide` has been deprecated. Please use `show.legend` instead.

fig2a + theme(axis.text = element_text(size = 12),
              axis.title = element_text(size = 12),
              strip.text = element_text(size = 12))
```

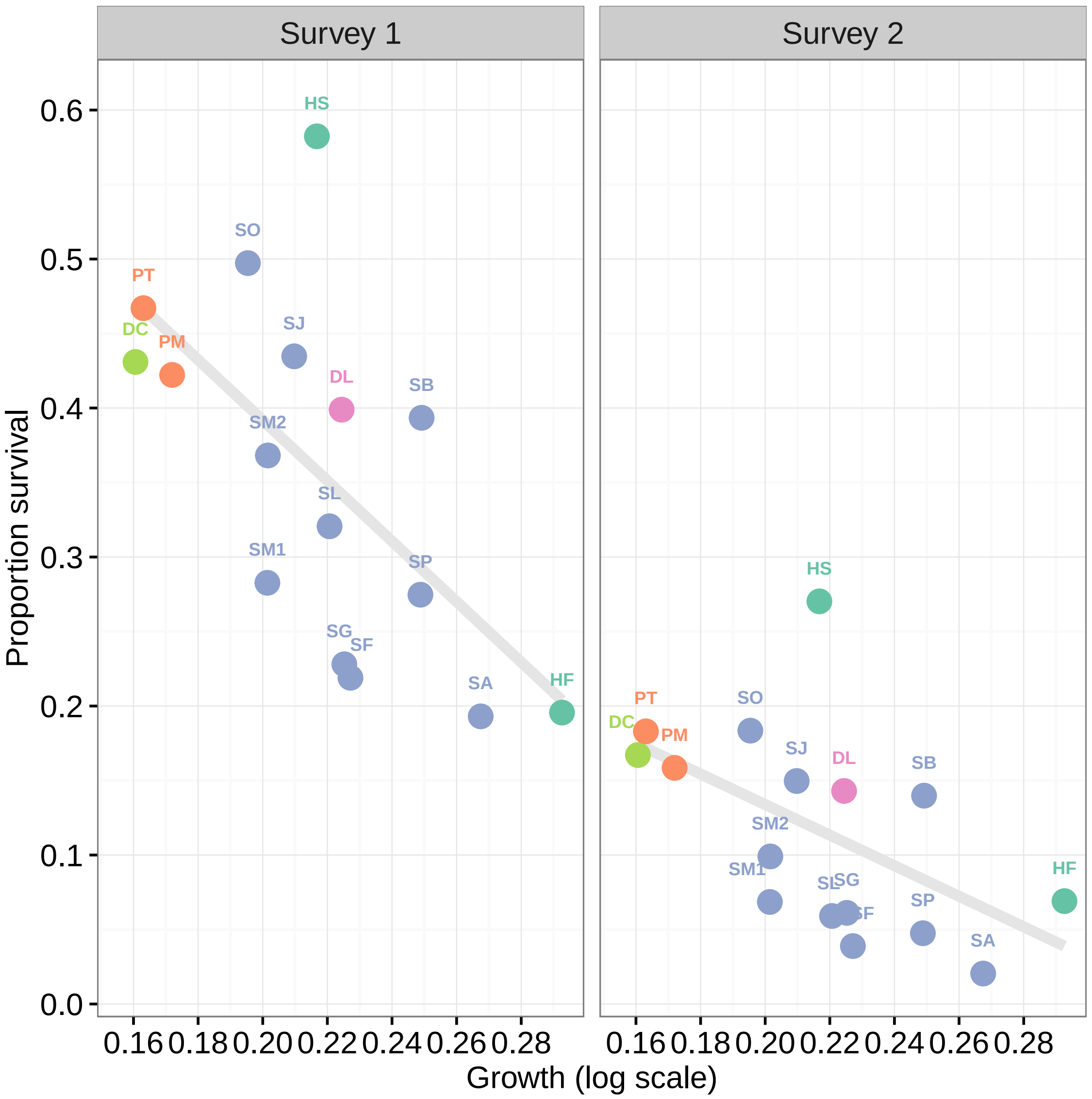


```
# ggsave("C:/Users/scro2435/Dropbox/PhD/_SBE/SBE_Project/Manuscript/Figures/sbeMsFig2a.png",
#        width = 8, height = 4, units = "in")
# pdf("C:/Users/scro2435/Dropbox/PhD/_SBE/SBE_Project/Manuscript/Figures/sbeMsFig2a.pdf",
# #      width = 8, height = 4)
# fig2a
# dev.off()
```

We can also show the survey 2 panel in the above figure, using just the survival between survey 1 and 2 (2-10 years).

```
fig2aSupp <- ggplot(speciesPerformance, aes(x = growthSlopes, y = surveySurvivals,
       colour = genus, label = codes)) + theme_bw() +
    geom_smooth(aes(group = 1), colour = "gray90",
                method = "lm", se = FALSE, size = 2) +
  geom_point(size = 4) + facet_grid(.~survey.type) +
  geom_text(aes(x = labelX, y = surveySurvivals+0.02), show_guide = FALSE,
              fontface = "bold", size = 3) +
  labs(y = "Proportion survival", x = "Growth (log scale)") +
  scale_y_continuous(breaks = seq(from = 0.0, to = 0.6, by = 0.1)) +
```

```
  scale_x_continuous(breaks = seq(from = 0.1, to = 0.3, by = 0.02)) +
  scale_colour_manual(values = with(speciesPerformance, c(
    Shorea = unique(colour[genus == "Shorea"]),
    Parashorea = unique(colour[genus == "Parashorea"]),
    Hopea = unique(colour[genus == "Hopea"]),
    Dryobalanops = unique(colour[genus == "Dryobalanops"]),
    Dipterocarpus = unique(colour[genus == "Dipterocarpus"])))) +
  theme(legend.position = "none")

Warning: `show_guide` has been deprecated. Please use `show.legend` instead.

fig2aSupp + theme(axis.text = element_text(size = 12),
              axis.title = element_text(size = 12),
              strip.text = element_text(size = 12))
```

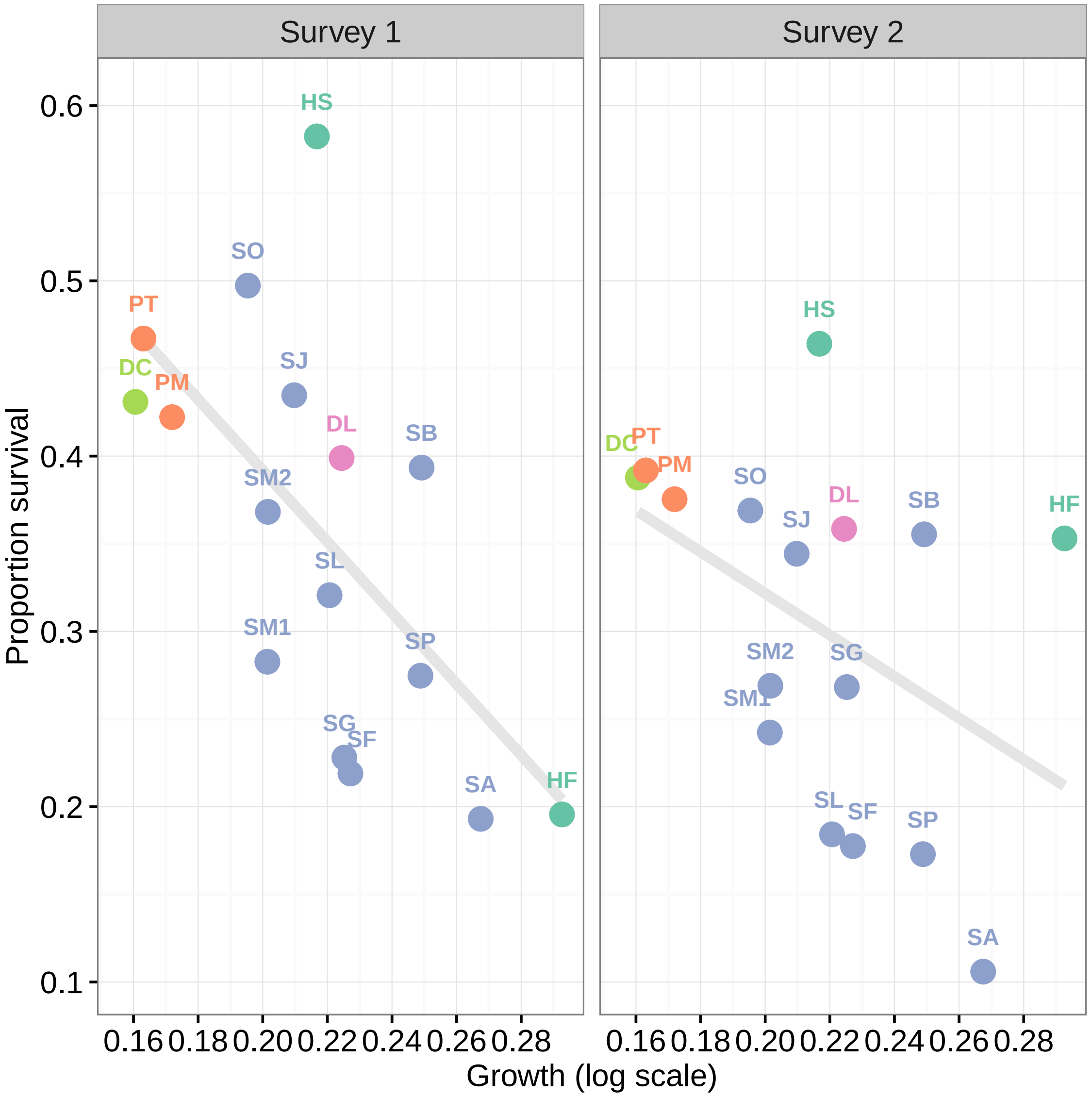


```
# ggsave("C:/Users/scro2435/Dropbox/PhD/_SBE/SBE_Project/Manuscript/Figures/sbeMsFig2a.png",
#        width = 8, height = 4, units = "in")
# pdf("C:/Users/scro2435/Dropbox/PhD/_SBE/SBE_Project/Manuscript/Figures/sbeMsFig2a.pdf",
#     width = 8, height = 4)
```

```
# fig2a
# dev.off()
```

Figure 2b shows the relationship between survival in the two inter-survey periods: survival in years 0-2 versus survival in years 2-10.

```
## Now plot correlation between survival in surveys 1 and 2
fig2bData <- reshape(speciesPerformance, direction = "wide",
                 idvar = "sp", timevar = "survey.type",
                 v.names = c("modelPreds","surveySurvivals","labelX"))
names(fig2bData)[8:13] <- c("modelPreds.1", "surveySurvivals.1", "labelX.1",
                        "modelPreds.2", "surveySurvivals.2", "labelX.2")
fig2bData$labelX.1 <- fig2bData$surveySurvivals.2
fig2bData$labelX.1[fig2bData$codes == "PM"] <-
  fig2bData$labelX.1[fig2bData$codes == "PM"] - 0.005
fig2bData$labelX.1[fig2bData$codes == "SB"] <-
  fig2bData$labelX.1[fig2bData$codes == "SB"] - 0.008
fig2bData$labelX.1[fig2bData$codes == "DL"] <-
  fig2bData$labelX.1[fig2bData$codes == "DL"] + 0.008

fig2b <- ggplot(fig2bData, aes(x = surveySurvivals.2, y = modelPreds.1,
       colour = genus, label = codes)) +
  geom_smooth(aes(group = 1), colour = "gray90",
             method = "lm", se = FALSE, size = 2) +
  geom_point(size = 4) + theme_bw() +
  geom_text(aes(x = labelX.1, y = modelPreds.1+0.015),
           show_guide = FALSE, fontface = "bold", size = 2.5) +
  labs(y = "Survival from planting to survey 1 (0-2 yr)",
       x = "Survival from survey 1 to survey 2 (2-10 yr)") +
  theme(axis.text = element_text(size = 12),
        axis.title.y = element_text(size = 12, vjust = 1),
        axis.title.x = element_text(size = 12, vjust = 0),
        strip.text = element_text(size = 12),
        legend.text = element_text(size = 12),
        legend.title = element_text(size = 12)) +
  guides(colour = guide_legend(title = "Genus")) +
    scale_y_continuous(breaks = seq(from = 0.0, to = 0.6, by = 0.05)) +
  scale_x_continuous(breaks = seq(from = 0.0, to = 0.6, by = 0.05)) +
  scale_colour_manual(values = with(speciesPerformance, c(
    Shorea = unique(colour[genus == "Shorea"]),
    Parashorea = unique(colour[genus == "Parashorea"]),
    Hopea = unique(colour[genus == "Hopea"]),
    Dryobalanops = unique(colour[genus == "Dryobalanops"]),
    Dipterocarpus = unique(colour[genus == "Dipterocarpus"]))))

Warning: `show_guide` has been deprecated. Please use `show.legend` instead.

fig2b
```

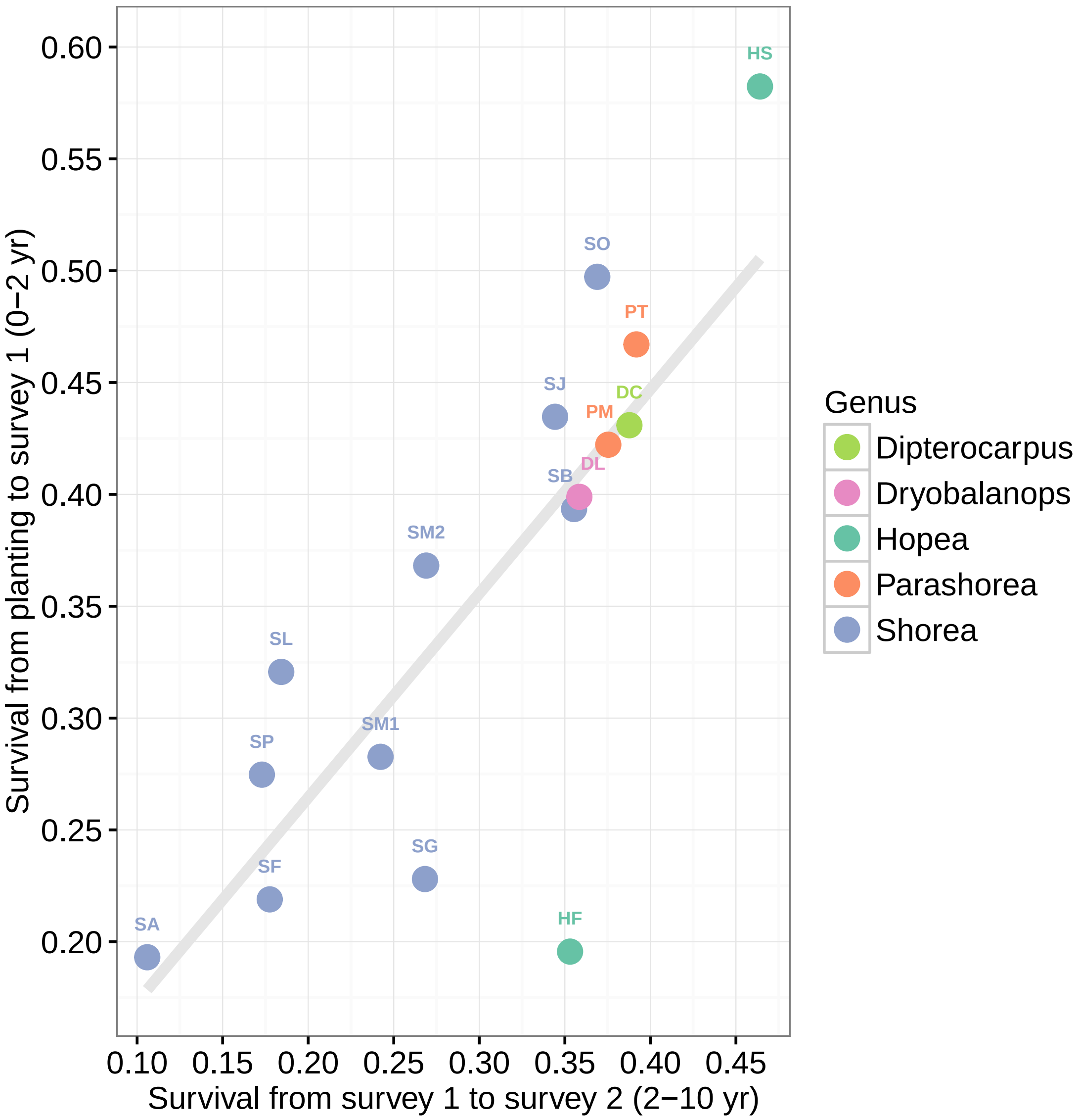


```
# ggsave("C:/Users/scro2435/Dropbox/PhD/_SBE/SBE_Project/Manuscript/Figures/sbeMsFig2b.png",
#        width = 8, height = 4, units = "in")
# pdf("C:/Users/scro2435/Dropbox/PhD/_SBE/SBE_Project/Manuscript/Figures/sbeMsFig2b.pdf",
#        width = 8, height = 4)
# fig2b
# dev.off()
```

To support these figures, and the growth-survival trade off they show, we will report Pearson correlation coefficients in the manuscript text. Showing slope estimates would not be very meaningful, because growth is on the log scale. And formal p-value inference for these estimates involves making many assumptions. Much better to support the clear relationships shown in figure 2 with simple correlations.

```
cor.test(speciesPerformance$growthSlopes[speciesPerformance$survey.type == "Survey 1"],
    speciesPerformance$surveySurvivals[speciesPerformance$survey.type == "Survey 1"])


Pearson's product-moment correlation
```

```
data:  speciesPerformance$growthSlopes[speciesPerformance$survey.type ==  and speciesPerformance
t = -3, df = 10, p-value = 0.009
alternative hypothesis: true correlation is not equal to 0
95 percent confidence interval:
 -0.86 -0.20
sample estimates:
  cor
-0.63
```

```
cor.test(speciesPerformance$growthSlopes[speciesPerformance$survey.type == "Survey 2"],
   speciesPerformance$surveySurvivals[speciesPerformance$survey.type == "Survey 2"])
```

```


Pearson's product-moment correlation

data:  speciesPerformance$growthSlopes[speciesPerformance$survey.type ==  and speciesPerformance
t = -2, df = 10, p-value = 0.1
alternative hypothesis: true correlation is not equal to 0
95 percent confidence interval:
 -0.761  0.088
sample estimates:
  cor
-0.43
```

```
cor.test(speciesPerformance$surveySurvivals[speciesPerformance$survey.type ==
   "Survey 1"], speciesPerformance$surveySurvivals[speciesPerformance$survey.type ==
    "Survey 2"])
```

```


Pearson's product-moment correlation

data:  speciesPerformance$surveySurvivals[speciesPerformance$survey.type ==  and speciesPerforma
t = 5, df = 10, p-value = 3e-04
alternative hypothesis: true correlation is not equal to 0
95 percent confidence interval:
 0.48 0.92
sample estimates:
 cor
0.79
```

Here are the growth and survival estimates:

```
speciesPerformance
```

```
                sp survey.type modelPreds codes        genus      section
1  S. argentifolia    Survey 1      0.193    SA       Shorea       Mutica
2    S. beccariana    Survey 1      0.393    SB       Shorea  Pachycarpae
3     D. conformis    Survey 1      0.431    DC Dipterocarpus    NotShorea
4    S. faguetiana    Survey 1      0.219    SF       Shorea Richetioides
5    H. ferruginea    Survey 1      0.196    HF        Hopea    NotShorea
6       S. gibbosa    Survey 1      0.228    SG       Shorea Richetioides
```

```
7    S. johorensis    Survey 1       0.435    SJ         Shorea Brachypterae
8    D. lanceolata    Survey 1       0.399    DL  Dryobalanops    NotShorea
9     S. leprosula    Survey 1       0.321    SL         Shorea       Mutica
10  S. macrophylla    Survey 1       0.283   SM1         Shorea  Pachycarpae
11   S. macroptera    Survey 1       0.368   SM2         Shorea  Auriculatae
12   P. malaanonan    Survey 1       0.422    PM     Parashorea    NotShorea
13       S. ovalis    Survey 1       0.497    SO         Shorea       Ovalis
14   S. parvifolia    Survey 1       0.275    SP         Shorea       Mutica
15       H. sangal    Survey 1       0.582    HS          Hopea    NotShorea
16   P. tomentella    Survey 1       0.467    PT     Parashorea    NotShorea
17 S. argentifolia    Survey 2       0.020    SA         Shorea       Mutica
18   S. beccariana    Survey 2       0.140    SB         Shorea  Pachycarpae
19    D. conformis    Survey 2       0.167    DC Dipterocarpus    NotShorea
20   S. faguetiana    Survey 2       0.039    SF         Shorea Richetioides
21   H. ferruginea    Survey 2       0.069    HF          Hopea    NotShorea
22      S. gibbosa    Survey 2       0.061    SG         Shorea Richetioides
23   S. johorensis    Survey 2       0.150    SJ         Shorea Brachypterae
24   D. lanceolata    Survey 2       0.143    DL  Dryobalanops    NotShorea
25    S. leprosula    Survey 2       0.059    SL         Shorea       Mutica
26  S. macrophylla    Survey 2       0.068   SM1         Shorea  Pachycarpae
27   S. macroptera    Survey 2       0.099   SM2         Shorea  Auriculatae
28   P. malaanonan    Survey 2       0.159    PM     Parashorea    NotShorea
29       S. ovalis    Survey 2       0.183    SO         Shorea       Ovalis
30   S. parvifolia    Survey 2       0.048    SP         Shorea       Mutica
31       H. sangal    Survey 2       0.270    HS          Hopea    NotShorea
32   P. tomentella    Survey 2       0.183    PT     Parashorea    NotShorea
    colour growthSlopes growthIntercepts labelX surveySurvivals
1  #8DA0CB         0.27             1.23   0.27            0.19
2  #8DA0CB         0.25             1.07   0.25            0.39
3  #A6D854         0.16             0.96   0.16            0.43
4  #8DA0CB         0.23             1.01   0.23            0.22
5  #66C2A5         0.29             0.89   0.29            0.20
6  #8DA0CB         0.23             1.01   0.22            0.23
7  #8DA0CB         0.21             1.05   0.21            0.43
8  #E78AC3         0.22             1.05   0.22            0.40
9  #8DA0CB         0.22             1.11   0.22            0.32
10 #8DA0CB         0.20             1.23   0.20            0.28
11 #8DA0CB         0.20             1.03   0.20            0.37
12 #FC8D62         0.17             1.01   0.17            0.42
13 #8DA0CB         0.20             1.09   0.20            0.50
14 #8DA0CB         0.25             1.09   0.25            0.27
15 #66C2A5         0.22             1.04   0.22            0.58
16 #FC8D62         0.16             1.01   0.16            0.47
17 #8DA0CB         0.27             1.23   0.27            0.11
18 #8DA0CB         0.25             1.07   0.25            0.36
19 #A6D854         0.16             0.96   0.16            0.39
20 #8DA0CB         0.23             1.01   0.23            0.18
21 #66C2A5         0.29             0.89   0.29            0.35
22 #8DA0CB         0.23             1.01   0.23            0.27
23 #8DA0CB         0.21             1.05   0.21            0.34
24 #E78AC3         0.22             1.05   0.22            0.36
25 #8DA0CB         0.22             1.11   0.22            0.18
```

```
26 #8DA0CB          0.20             1.23   0.19             0.24
27 #8DA0CB          0.20             1.03   0.20             0.27
28 #FC8D62          0.17             1.01   0.17             0.38
29 #8DA0CB          0.20             1.09   0.20             0.37
30 #8DA0CB          0.25             1.09   0.25             0.17
31 #66C2A5          0.22             1.04   0.22             0.46
32 #FC8D62          0.16             1.01   0.16             0.39
```

8.2 Main text results: correlations with traits

Trait correlations with survival and growth: use wood density and specific leaf area, and then try traits from initial planting seedlings.

```
data("sbeTraits")

sbeTraits$sp <- with(sbeTraits,
                     paste0(substr(species, 1, 1), ". ",
                            substr(species,
                                   regexpr("_", species)+1,
                                   nchar(as.character(species)))))

performance <- speciesPerformance[order(speciesPerformance$sp), ]

## Correlate survival after 10 years with wood density
cor(subset(performance, survey.type == "Survey 2")$modelPreds,
    sbeTraits$wood.density, use = "complete.obs")

[1] 0.78

## Correlate survival after ten years with SLA
cor(subset(performance, survey.type == "Survey 2")$modelPreds,
    sbeTraits$sla, use = "complete.obs")

[1] 0.063

## Correlate growth with wood density
cor(subset(performance, survey.type == "Survey 2")$growthSlopes,
    sbeTraits$wood.density, use = "complete.obs")

[1] -0.5

## Correlate growth with SLA
cor(subset(performance, survey.type == "Survey 2")$growthSlopes,
    sbeTraits$sla, use = "complete.obs")

[1] -0.14
```

8.3 Figure 3

Show the magnitude of plot-level variation within species, for all species, and connect plot with lines to show whether species respond to plot conditions in similar ways. Support this with a likelihood ratio test of nested model without sp:plot interaction in random effect.

```
plotFx <- ranef(survivalModel)$"sp.pl"
names(plotFx)[1] <- "plotEffect"
plotFx$sp <- sub("\\..*", "", rownames(plotFx))
plotFx$pl <- sub("^.*?\\.", "", rownames(plotFx))
plotFx$sd <- sapply(plotFx$pl,
                    function(x) unique(survival$sd[survival$pl == x]))
plotFx$plotLines <- ifelse(plotFx$sd == 16, plotFx$plotEffect, NA)

plotFx$sp <- as.character(plotFx$sp)
plotFx$sp[plotFx$sp == "argentifolia"] <- "S. argentifolia"
plotFx$sp[plotFx$sp == "beccariana"] <- "S. beccariana"
plotFx$sp[plotFx$sp == "conformis"] <- "D. conformis"
plotFx$sp[plotFx$sp == "faguetiana"] <- "S. faguetiana"
plotFx$sp[plotFx$sp == "ferruginea"] <- "H. ferruginea"
plotFx$sp[plotFx$sp == "gibbosa"] <- "S. gibbosa"
plotFx$sp[plotFx$sp == "johorensis"] <- "S. johorensis"
plotFx$sp[plotFx$sp == "lanceolata"] <- "D. lanceolata"
plotFx$sp[plotFx$sp == "leprosula"] <- "S. leprosula"
plotFx$sp[plotFx$sp == "macrophylla"] <- "S. macrophylla"
plotFx$sp[plotFx$sp == "macroptera"] <- "S. macroptera"
plotFx$sp[plotFx$sp == "malaanonan"] <- "P. malaanonan"
plotFx$sp[plotFx$sp == "ovalis"] <- "S. ovalis"
plotFx$sp[plotFx$sp == "parvifolia"] <- "S. parvifolia"
plotFx$sp[plotFx$sp == "sangal"] <- "H. sangal"
plotFx$sp[plotFx$sp == "tomentella"] <- "P. tomentella"

## Order species by spatial variability.
plotFx$sp <- factor(plotFx$sp,
                    levels = unique(plotFx$sp)[order(sapply(
                      unique(plotFx$sp), function(x)
                        var(plotFx$plotEffect[plotFx$sp == x])
                      ))]
                    )

plotFx$lineCol <- with(plotFx,
                       ifelse(pl == pl[which.max(plotEffect)],
                       "red",
                       "gray60"))
plotFx$lineWidth <- with(plotFx,
                         ifelse(pl == pl[which.max(plotEffect)], 2, 1))
plotFx$pointCol <- with(plotFx,
                        ifelse(pl == pl[which.max(plotEffect)],
                        "#CC6666",
                        "gray10"))
```

8.3.1 Figure 3a

```
ggplot(plotFx, aes(x = sp, y = plotEffect, group = pl)) +
  geom_line(aes(y = plotLines, group = pl), colour = plotFx$lineCol,
            size = plotFx$lineWidth, alpha = 0.2) +
  geom_point(size = 3, colour = plotFx$pointCol) + theme_bw() +
  labs(x = "Species",
       y = "Plot-level relative effect on proportion survival") +
  coord_flip() +
  theme(axis.title = element_text(size = 16),
        axis.text = element_text(size = 16))
```

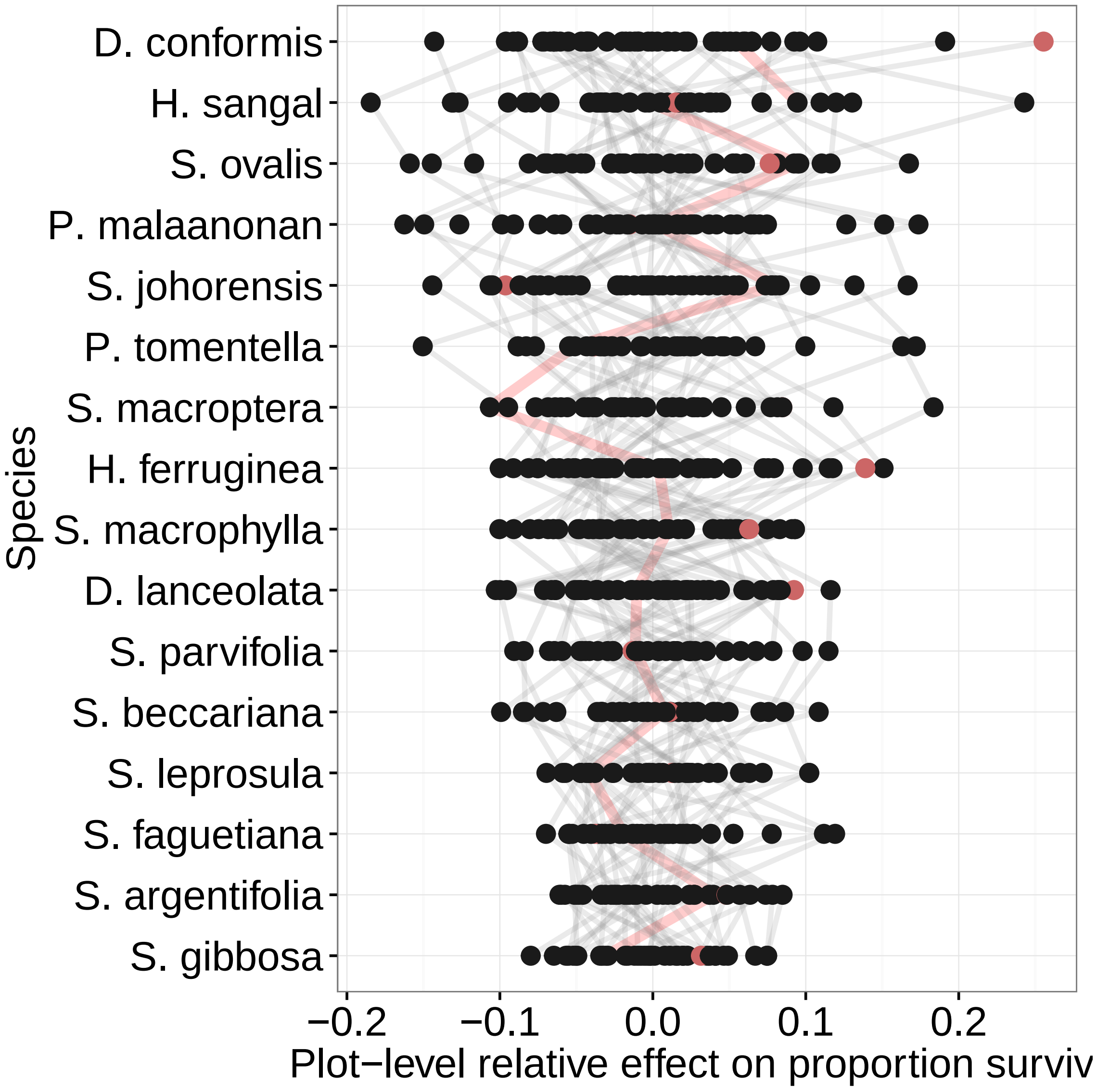


```
growthRanef <- ranef(growthModel)[[1]]

growthRanef$sp <- sub("\\..*", "", rownames(growthRanef))
growthRanef$pl <- sub("^.*?\\.", "", rownames(growthRanef))

names(growthRanef)[1] <- "r.int"
growthRanef$sp.pl <- rownames(growthRanef)
```

```r
rownames(growthRanef) <- NULL

growthRanef$sd <- sapply(growthRanef$pl,
                        function(x) unique(survival$sd[survival$pl == x]))
growthRanef$plotLines <- ifelse(growthRanef$sd == 16, growthRanef$r.int, NA)

growthRanef$sp <- as.character(growthRanef$sp)
growthRanef$sp[growthRanef$sp == "argentifolia"] <- "S. argentifolia"
growthRanef$sp[growthRanef$sp == "beccariana"] <- "S. beccariana"
growthRanef$sp[growthRanef$sp == "conformis"] <- "D. conformis"
growthRanef$sp[growthRanef$sp == "faguetiana"] <- "S. faguetiana"
growthRanef$sp[growthRanef$sp == "ferruginea"] <- "H. ferruginea"
growthRanef$sp[growthRanef$sp == "gibbosa"] <- "S. gibbosa"
growthRanef$sp[growthRanef$sp == "johorensis"] <- "S. johorensis"
growthRanef$sp[growthRanef$sp == "lanceolata"] <- "D. lanceolata"
growthRanef$sp[growthRanef$sp == "leprosula"] <- "S. leprosula"
growthRanef$sp[growthRanef$sp == "macrophylla"] <- "S. macrophylla"
growthRanef$sp[growthRanef$sp == "macroptera"] <- "S. macroptera"
growthRanef$sp[growthRanef$sp == "malaanonan"] <- "P. malaanonan"
growthRanef$sp[growthRanef$sp == "ovalis"] <- "S. ovalis"
growthRanef$sp[growthRanef$sp == "parvifolia"] <- "S. parvifolia"
growthRanef$sp[growthRanef$sp == "sangal"] <- "H. sangal"
growthRanef$sp[growthRanef$sp == "tomentella"] <- "P. tomentella"

## Order species by spatial variability.
growthRanef$sp <- factor(growthRanef$sp,
  levels = unique(growthRanef$sp)[order(
    sapply(unique(growthRanef$sp),function(x)
      var(growthRanef$r.int[growthRanef$sp == x]))
    )])

growthRanef$lineCol <- ifelse(
  growthRanef$pl == growthRanef$pl[which.max(growthRanef$r.int)],
  "red",
  "gray60")

growthRanef$lineWidth <- ifelse(
  growthRanef$pl == growthRanef$pl[which.max(growthRanef$r.int)],
  2,
  1)

growthRanef$pointCol <- ifelse(
  growthRanef$pl == growthRanef$pl[which.max(growthRanef$r.int)],
  "#CC6666",
  "gray10")
```

8.3.2 Figure 3b Supplementary

```
ggplot(growthRanef, aes(x = sp, y = r.int, group = pl)) +
  geom_line(aes(y = plotLines, group = pl), colour = growthRanef$lineCol,
            size = growthRanef$lineWidth, alpha = 0.2) +
  geom_point(size = 3, colour = growthRanef$pointCol) + theme_bw() +
  labs(x = "Species",
       y = "Plot-level relative effect on growth (log scale)") +
  coord_flip() +
  theme(axis.title = element_text(size = 16),
        axis.text = element_text(size = 16))
```

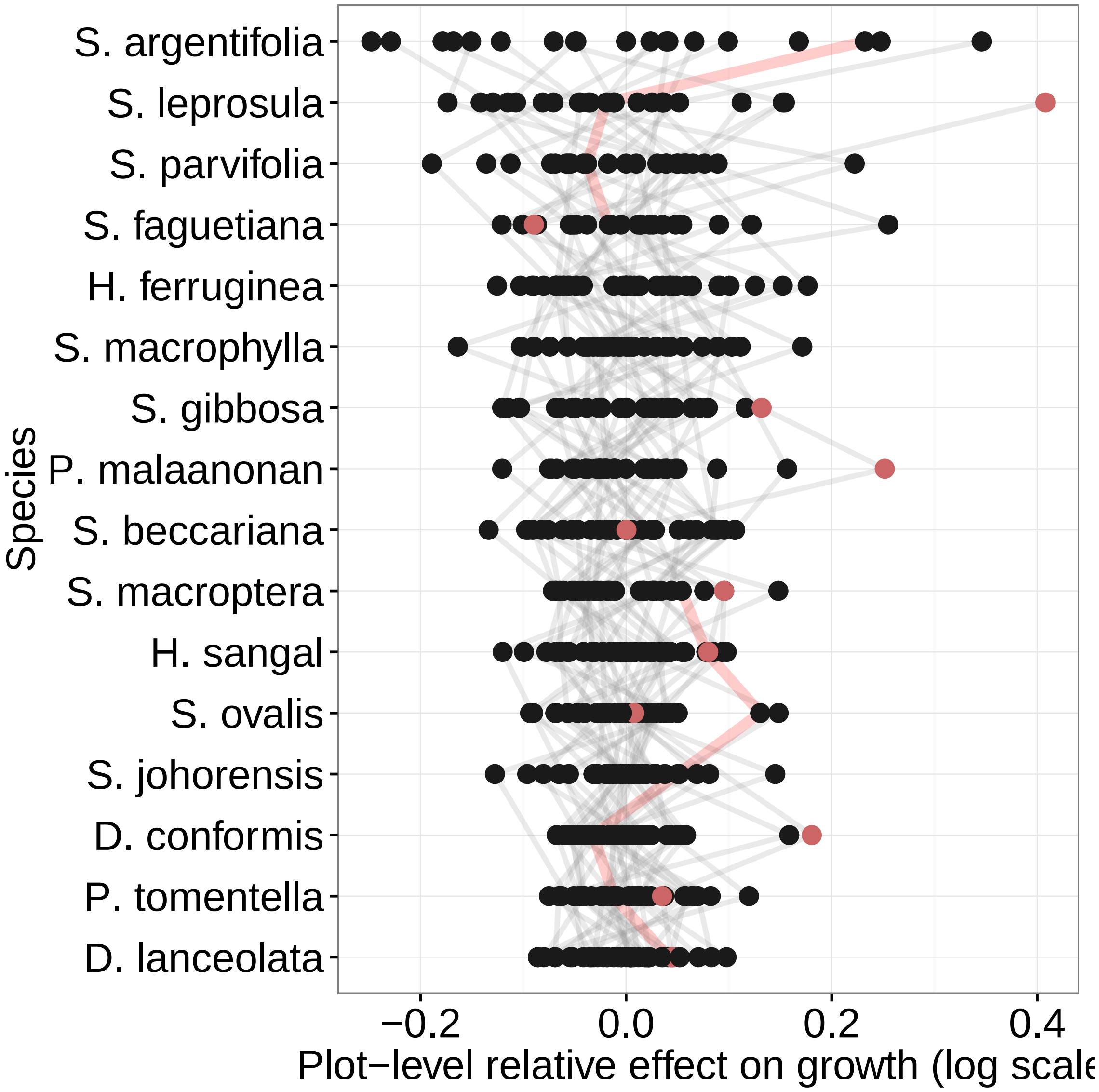


```
## Test the species specificity on spatial variation with a nested model
## where species respond to plot conditions in the same way.
survivalModelAic <- lmer(survival ~ spBySurveyByComp + (1|pl),
                         data = survival)
anova(survivalModel, survivalModelAic)

refitting model(s) with ML (instead of REML)
```

```
Data: survival
Models:
survivalModel: survival ~ spBySurveyByComp + (1 | sp.pl)
survivalModelAic: survival ~ spBySurveyByComp + (1 | pl)
                  Df   AIC   BIC logLik deviance Chisq Chi Df Pr(>Chisq)
survivalModel    194 -2418 -1410   1403    -2806
survivalModelAic 194 -2399 -1391   1394    -2787     0      0          1

growthModelAic <- lmer(mean.log.diam ~ SP.sp.comp * mdsp.sc + (1|pl),
                       data = growth)
anova(growthModel, growthModelAic)

refitting model(s) with ML (instead of REML)

Data: growth
Models:
growthModel: mean.log.diam ~ SP.sp.comp * mdsp.sc + (1 | sp.pl)
growthModelAic: mean.log.diam ~ SP.sp.comp * mdsp.sc + (1 | pl)
                Df  AIC   BIC logLik deviance Chisq Chi Df Pr(>Chisq)
growthModel    194 -977 -2.15    682    -1365
growthModelAic 194 -960 14.34    674    -1348     0      0          1
```

The lines that link a specific (16-species mixture) plot from species to species show a large amount of crossing, suggesting that species respond to plot conditions independently - a plot that is bad for one species may not be bad for another. This is demonstrated by one plot highlighted in red, that shows the highest survival.

8.4 Figure 4

Show evidence for an insurance effect of increasing species richness on mortality and growth by showing the plot-level responses as a function of species richness. If there is any evidence for an insurance effect, the mean growth/survival at higher richness will be lower than the maximum but higher than the minimum at lower richness and, crucially, variation around that mean will be lower at higher richness.

```
sdPlotSummary <- summaryBy(survival ~ pl + survey.type,
                           data = msSurv, id = ~ sd + sp.comp,
                           FUN = c(perHectare = function(x)
                                      sum(x, na.rm=T) / 4,
                                   proportion = function(x)
                                      sum(x, na.rm=T) / sum(x<=1, na.rm=T),
                                   successes = function(x)
                                      sum(x, na.rm=T),
                                   trials = function(x)
                                      length(x)),
                           fun.names = c("perHectare", "proportion",
                                         "successes", "trials"))



mf_labeller <- function(var, value){
    value <- as.character(value)
```

```
    if (var == "survey.type" | var == "surv") {
        value[value=="census1"] <- "Survey 1"
        value[value=="census2"] <- "Survey 2"
    }
    return(value)
}
```

8.4.1 Figure 4a

```
ggplot(sdPlotSummary,
       aes(x=factor(sd), y=survival.perHectare)) +
  geom_boxplot(colour="gray40", fill="gray70", size=0.8, outlier.size=3) +
  theme_bw() +
  labs(x="Species richness",
       y="Number of surviving trees per hectare") +
  theme(axis.text=element_text(size=20),
        axis.title=element_text(size=20),
        strip.text=element_text(size=20)) +
  facet_grid(.~survey.type, labeller=mf_labeller)

Error in labeller(label_df): argument "value" is missing, with no default
```

```r
# ggsave(paste0("C:/Users/scro2435/Dropbox/PhD/_SBE/",
#               "SBE_Project/Manuscript/Figures/sbeMsFig4.png"))
```

```r
########################
ggplot(subset(sdPlotSummary, survey.type == "census1"),
       aes(x = factor(sd), y = survival.perHectare)) +
  geom_hline(yintercept = 15, colour = "#CC6666", lwd = 1.5) +
  geom_boxplot(colour = "gray40", fill = "gray70") + theme_bw() +
  geom_text(y = 12, x = 2, label = "Minimum density for canopy closure",
            hjust = 0, colour = "#CC6666", size = 6) +
  xlab("Species richness") + ylab("Number of surviving trees per hectare") +
  theme(axis.text = element_text(size = 16),
        axis.title = element_text(size = 18))
```

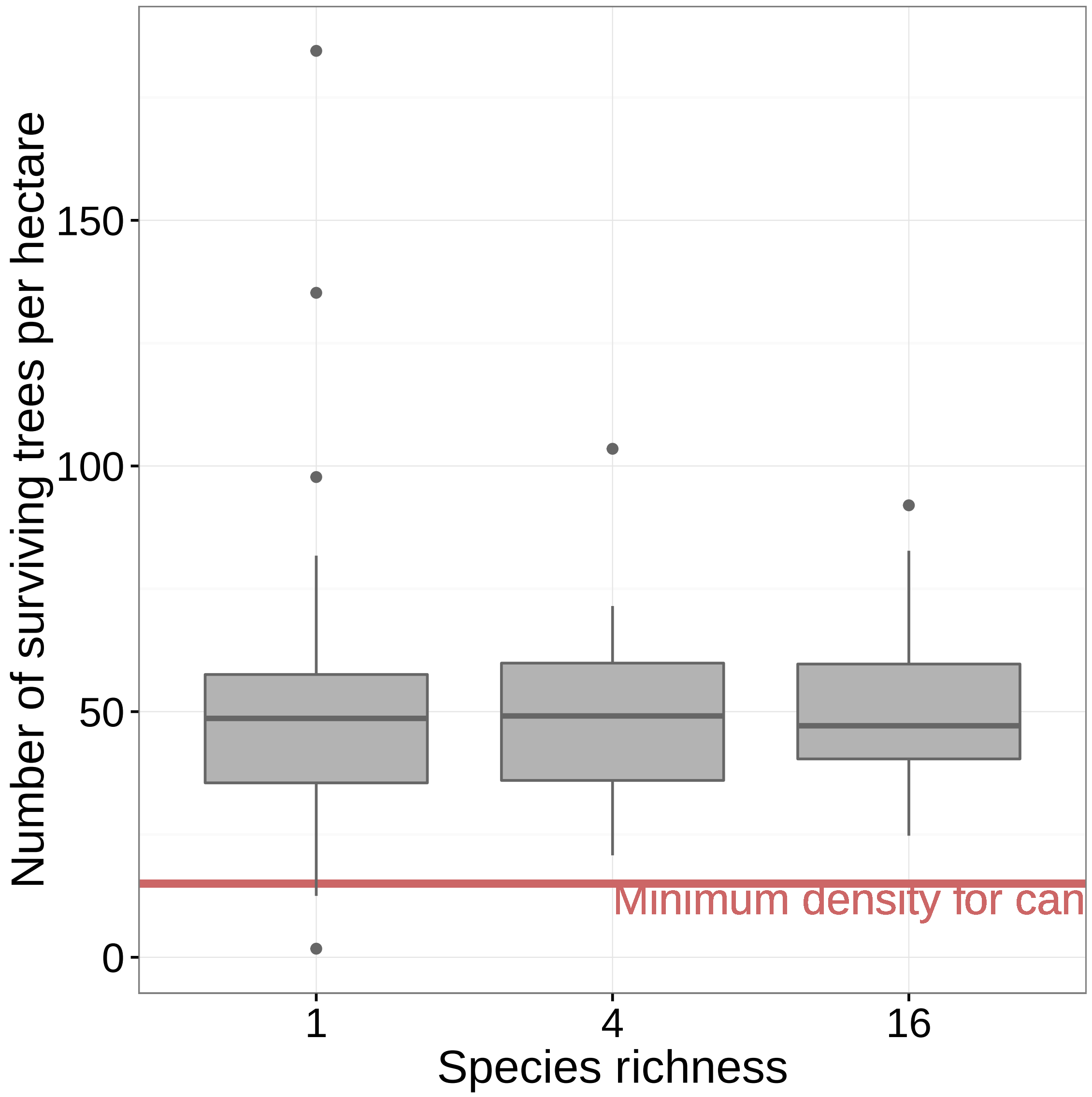


```
##########################

growthSummary <- aggregate(
  x = list(stemAreaPerHectare=((pi * (msGrowth$d.base/2)^2) / 4)),
  by = list(pl=msGrowth$pl, surv=msGrowth$survey.type, sd=msGrowth$sd,
            sp.comp=msGrowth$sp.comp),
  FUN = sum)
## Get total stem area mm^2 per hectare by calculating
## sum(pi * (msGrowth$d.base / 2)^2) / 4
## can log transform for plotting reasons if necessary.
```

8.4.2 Figure 4b

```
ggplot(growthSummary,
       aes(x = factor(sd), y = log(stemAreaPerHectare / 100))) +
  geom_boxplot(colour = "gray40", fill = "gray70") + theme_bw() +
  xlab("Species richness") +
  ylab(substitute(paste(s1, log[10](cm^2 / s2)),
```

```
                  list(s1 = "Total stem area: ", s2 = " hectare"))) +
  theme(axis.text = element_text(size = 10),
        axis.title = element_text(size = 10)) +
  facet_grid(.~surv, labeller = mf_labeller)

Error in labeller(label_df): argument "value" is missing, with no default
```

8.5 Figure 5

Now show the survival and stem area per hectare broken down into species compositions, with their average effects, to see if any particular species/compositions are faring especially poorly.

```
sdPlotSummary$sp.comp <- factor(sdPlotSummary$sp.comp,
    levels = rev(c("sixteen","4.1","4.2","4.3","4.4","4.5","4.6","4.7","4.8",
               "4.9","4.10","4.11","4.12","4.13","4.14","4.15","4.16",
               "argentifolia","beccariana","conformis","faguetiana",
               "ferruginea","gibbosa","johorensis","lanceolata",
               "leprosula","macrophylla","macroptera","malaanonan",
```

```
                "ovalis","parvifolia","sangal","tomentella")))
levels(sdPlotSummary$sp.comp)[levels(sdPlotSummary$sp.comp)=="sixteen"] <- "16"

spCompMeans <- summaryBy(
 survival.perHectare + survival.proportion + survival.successes + survival.trials ~ sp.comp + sur
  data = sdPlotSummary, FUN = mean, keep.names = TRUE)
sdPlotSummary <- rbind(
  data.frame(sdPlotSummary, pointType = "NotMean"),
  with(spCompMeans,
       data.frame(pl = NA, survey.type, survival.perHectare,
                  survival.proportion, survival.successes, survival.trials,
                  sd, sp.comp, pointType = "Mean"))
  )

# sixteenStdv <- sd(with(sdPlotSummary,
#  survival.perHectare[sp.comp=="16" & survey.type=="census2" & pointType=="NotMean"]))
# sixteenMean <- mean(with(sdPlotSummary,
#  survival.perHectare[sp.comp=="16" & survey.type=="census2" & pointType=="NotMean"]))
# ribbon <- data.frame(x = 0:18,
#                      y = rep(sixteenMean, 19),
#                      ymax = rep(sixteenMean + sixteenStdv, 19),
#                      ymin = rep(sixteenMean - sixteenStdv, 19))

ribbonConfint <- with(
 subset(sdPlotSummary, sp.comp=="16" & survey.type=="census2" & pointType=="NotMean"),
 binom.confint(x=mean(survival.successes), n=mean(survival.trials), conf.level=.95, methods="wi
  )
numHectaresPerPlot <- 4
ribbon <- data.frame(
  x = 0:18,
  y = (ribbonConfint$mean * ribbonConfint$n) / numHectaresPerPlot,
  ymax = (ribbonConfint$upper * ribbonConfint$n) / numHectaresPerPlot,
  ymin = (ribbonConfint$lower * ribbonConfint$n) / numHectaresPerPlot
  )

fig5 <- ggplot(subset(sdPlotSummary, sd != 4 & survey.type == "census2"),
       aes(y = survival.perHectare, x = sp.comp)) +
  geom_blank() +
  geom_ribbon(data = ribbon, aes(x = x, y = y, ymax = ymax, ymin = ymin),
              fill = "grey90") +
  geom_point(aes(size = pointType)) + theme_bw() +
  labs(y = "Number of trees per hectare",
       x = "Species compositions") +
  theme(legend.position = "none",
        axis.title.y = element_text(size = 12, vjust = 1),
        axis.title.x = element_text(size = 12, vjust = 0),
        axis.text = element_text(size = 12),
        strip.text = element_text(size = 12)) + coord_flip() +
  scale_size_manual(values = c(3,7))
fig5
```

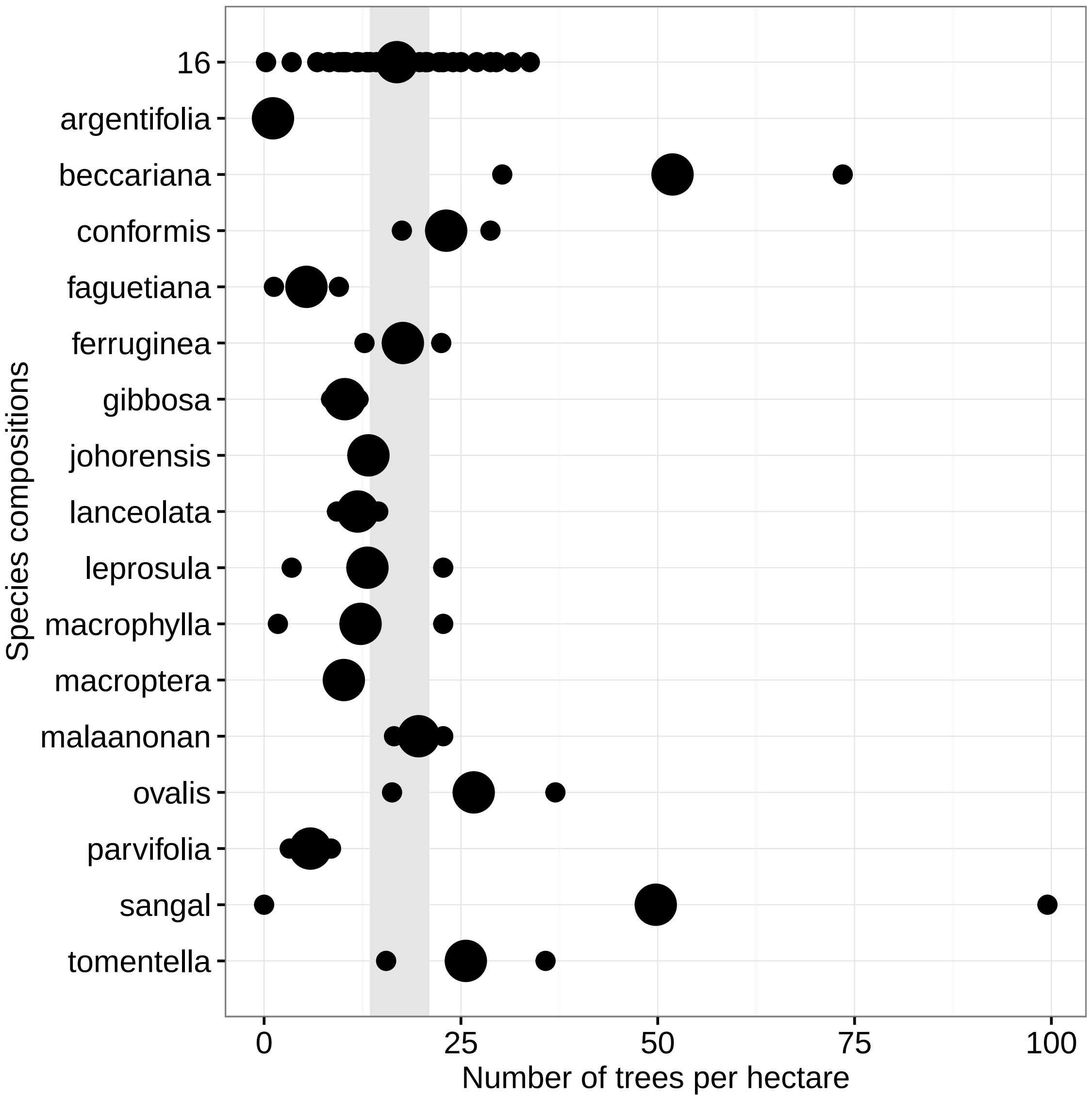


```
# ggsave("C:/Users/scro2435/Dropbox/PhD/_SBE/SBE_Project/Manuscript/Figures/sbeMsFig5.png",
#        width = 7, height = 7, units="in")
# pdf("C:/Users/scro2435/Dropbox/PhD/_SBE/SBE_Project/Manuscript/Figures/sbeMsFig5.pdf",
#        width = 7, height = 7)
# fig5
# dev.off()
```

9 Supplementary figures

```
growthSummary$sp.comp <- factor(growthSummary$sp.comp,
  levels = c("argentifolia","beccariana","conformis","faguetiana",
             "ferruginea","gibbosa","johorensis","lanceolata","leprosula",
             "macrophylla","macroptera","malaanonan","ovalis","parvifolia",
             "sangal","tomentella","4.1","4.2","4.3","4.4","4.5","4.6",
             "4.7","4.8","4.9","4.10","4.11","4.12","4.13","4.14","4.15",
             "4.16","sixteen"))
levels(growthSummary$sp.comp)[levels(growthSummary$sp.comp)=="sixteen"] <- "16"
```

```
growthMeans <- summaryBy(stemAreaPerHectare ~ sp.comp + surv + sd,
                         data = growthSummary, FUN = mean, keep.names = TRUE)
growthSummary <- rbind(
  data.frame(growthSummary, pointType = "NotMean"),
  with(growthMeans, data.frame(pl = NA, surv, sd, sp.comp,
                               stemAreaPerHectare, pointType = "Mean")))

ggplot(growthSummary, aes(x = stemAreaPerHectare, y = sp.comp,
       alpha = pointType, size = 4)) + geom_point() + theme_bw() +
  facet_grid(.~surv, labeller = mf_labeller, scales = "free") +
  labs(x = substitute(paste(s1, log[10](cm^2 / s2)),
                    list(s1 = "Total stem area: ", s2 = " hectare")),
       y = "Species compositions") +
  theme(legend.position = "none",
        axis.title.y = element_text(size = 14, vjust = 1),
        axis.title.x = element_text(size = 14),
        axis.text = element_text(size = 12),
        strip.text = element_text(size = 12))

Error in labeller(label_df): argument "value" is missing, with no default
```

```
compositionMeans <- summaryBy(survival.perHectare ~ sp.comp + survey.type +
    sd, data = subset(sdPlotSummary, pointType == "NotMean"), FUN = c(mean = mean,
    SE = function(x) sd(x, na.rm = TRUE)/sqrt(length(x))), fun.names = c("mean",
    "SE"))
compositionMeans <- subset(compositionMeans, sd != 4)

ggplot(subset(compositionMeans, survey.type == "census1"), aes(y = survival.perHectare.mean,
   x = sp.comp)) + geom_pointrange(aes(ymax = survival.perHectare.mean + survival.perHectare.SE,
    ymin = survival.perHectare.mean - survival.perHectare.SE), size = 1, width = 1) +
   coord_flip() + theme_bw() + labs(x = "Species compositions", y = "Average surviving trees per he
    theme(axis.text = element_text(size = 14), axis.title = element_text(size = 16),
        legend.position = "none")

Error: Unknown parameters: width

diversityMeans <- summaryBy(survival.perHectare ~ survey.type + sd, data = subset(sdPlotSummary,
   pointType == "NotMean"), FUN = c(mean = mean, SE = function(x) sd(x, na.rm = TRUE)/sqrt(length(x
    fun.names = c("mean", "SE"))
```

```
ggplot(subset(diversityMeans, survey.type == "census1"), aes(y = survival.perHectare.mean,
    x = factor(sd))) + geom_pointrange(aes(ymax = survival.perHectare.mean +
   survival.perHectare.SE, ymin = survival.perHectare.mean - survival.perHectare.SE),
   size = 1, width = 1) + theme_bw() + labs(x = "Species richness", y = "Average surviving trees per
    theme(axis.text = element_text(size = 14), axis.title = element_text(size = 16),
        legend.position = "none")

Error: Unknown parameters: width
```

Add the second cohort trees to calculations of density.

```
data(sbeData)

cohort2 <- data.frame(
  survival = c(subset(msSurv, survey.type=="census2")$survival,
               sbeData$survival_census2[grepl("N", sbeData$tree.id)]),
  pl = c(subset(msSurv, survey.type=="census2")$pl,
         sbeData$pl[grepl("N", sbeData$tree.id)]),
  sd = c(as.character(subset(msSurv, survey.type=="census2")$sd),
         as.character(sbeData$sd[grepl("N", sbeData$tree.id)])),
  sp.comp = c(as.character(subset(msSurv, survey.type=="census2")$sp.comp),
              as.character(sbeData$sp.comp[grepl("N", sbeData$tree.id)])))

cohort2 <- subset(cohort2, sp.comp != "sixteen+CC")

cohortSummary <- summaryBy(survival ~ pl,
                     data = cohort2,
                     id = ~ sd + sp.comp,
                     FUN = c(
                       perHectare = function(x)
                         sum(x, na.rm = TRUE) / 4,
                       proportion = function(x)
                         sum(x, na.rm = TRUE) / sum(x <= 1, na.rm=TRUE)),
                     fun.names = c("perHectare", "proportion"))

cohortSummary$sp.comp <- factor(cohortSummary$sp.comp,
    levels = rev(
      c("sixteen","4.1","4.2","4.3","4.4","4.5","4.6","4.7","4.8","4.9",
        "4.10","4.11","4.12","4.13","4.14","4.15","4.16","argentifolia",
        "beccariana","conformis","faguetiana","ferruginea","gibbosa",
        "johorensis","lanceolata","leprosula","macrophylla","macroptera",
        "malaanonan","ovalis","parvifolia","sangal","tomentella")))
levels(cohortSummary$sp.comp)[levels(cohortSummary$sp.comp)=="sixteen"] <-
  "16"

cohortMeans <- summaryBy(
  survival.perHectare + survival.proportion ~ sp.comp + sd,
  data = cohortSummary, FUN = mean, keep.names = TRUE)
cohortSummary <- rbind(
  data.frame(cohortSummary, pointType = "NotMean"),
  with(cohortMeans,
       data.frame(pl = NA, survival.perHectare, survival.proportion,
```

```
                  sd, sp.comp, pointType = "Mean"))
  )

cohort16Stdv <- sd(with(cohortSummary,
        survival.perHectare[sp.comp == "16" & pointType == "NotMean"]))
cohort16Mean <- mean(with(cohortSummary,
        survival.perHectare[sp.comp == "16" & pointType == "NotMean"]))

cohortRibbon <- data.frame(
  x = 0:18,
  y = rep(cohort16Mean, 19),
  ymax = rep(cohort16Mean + cohort16Stdv, 19),
  ymin = rep(cohort16Mean - cohort16Stdv, 19))

ggplot(subset(cohortSummary, sd != 4),
       aes(y = survival.perHectare, x = sp.comp)) +
  geom_blank() +
  geom_ribbon(data = cohortRibbon, fill = "grey90",
              aes(x = x, y = y, ymax = ymax,ymin = ymin)) +
  geom_point(aes(size = pointType)) + theme_bw() +
  labs(y = "Number of trees per hectare",
       x = "Species compositions") +
  theme(legend.position = "none",
        axis.title.y = element_text(size=16, vjust=1),
        axis.title.x = element_text(size=16, vjust=0),
        axis.text.y = element_text(size=16),
        axis.text.x = element_text(size=16, angle=45, vjust=1, hjust=1),
        strip.text = element_text(size=16)) + coord_flip() +
  scale_size_manual(values=c(3,7))
```

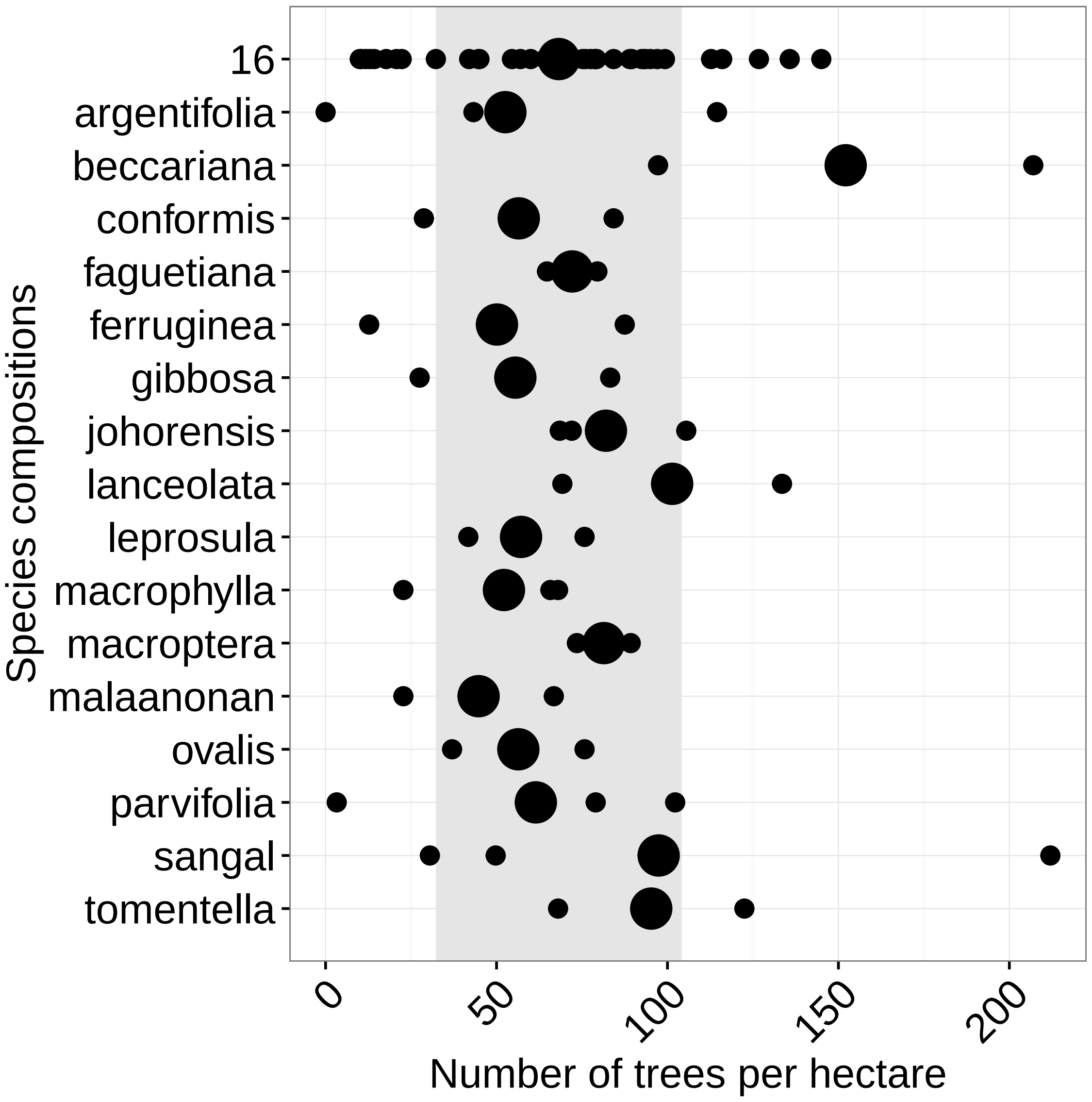
16
argentifolia
beccariana
conformis
faguetiana
ferruginea
gibbosa
johorensis
lanceolata
leprosula
macrophylla
macroptera
malaanonan
ovalis
parvifolia
sangal
tomentella
Species compositions
0
50
100
150
200
Number of trees per hectare
